# Exploring Regional Reduction Pathways for Human Exposure to Fine Particulate Matter (PM_2.5_) Using a Traffic Assignment Model

**DOI:** 10.1021/acs.est.3c05594

**Published:** 2023-11-13

**Authors:** Ahmad Bin Thaneya, Arpad Horvath

**Affiliations:** Department of Civil and Environmental Engineering, University of California, Berkeley, California 94720, United States

**Keywords:** transportation, traffic assignment, air quality, human health, exposure

## Abstract

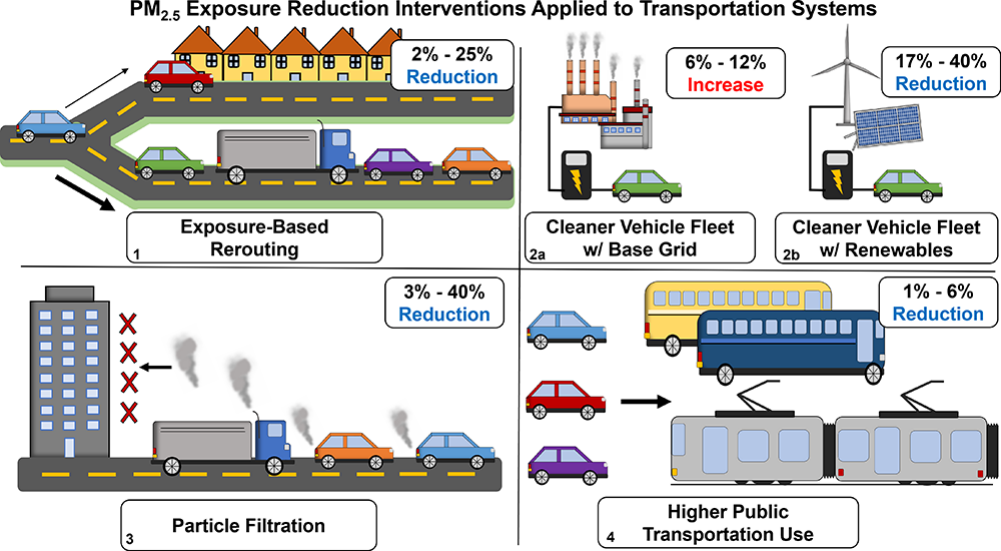

An exposure-based traffic assignment (TA) model is used to quantify primary and secondary fine particulate matter (PM_2.5_) exposure from on-road vehicle flow on the Chicago Metropolitan Area regional network. PM_2.5_ exposure due to emissions from light-duty vehicles, heavy-duty trucks, public transportation, and electricity generation for electric vehicle charging and light-rail transportation is considered. The model uses travel demand data disaggregated by time-of-day period and vehicle user class to compare the exposure impacts of two TA optimization scenarios: a baseline user equilibrium with respect to travel time (UET) and a system optimal with respect to pollutant intake (SOI). Estimated baseline PM_2.5_ exposure damages are $3.7B–$8.3B/year. The SOI uses exposure-based vehicle rerouting to reduce total damages by 8.2%, with high-impacted populations benefiting from 10% to 20% reductions. However, the SOI’s rerouting principle leads to a 66% increase in travel time. The model is then used to quantify the mitigation potential of different exposure reduction strategies, including a bi-objective optimization formulation that minimizes travel time and PM_2.5_ exposure concurrently, adoption of a cleaner vehicle fleet, higher public transportation use, particle filtration, and exposure-based truck routing. Exposure reductions range between 1% and 40%, but collective adoption of all strategies would lead to reductions upward of 50%.

## Abstract 2

Human exposure to fine particulate matter (PM_2.5_) from traffic emissions is a global health problem. This study uses a traffic assignment model to explore different ways in which transportation networks can be managed to mitigate PM_2.5_ exposure without severely impacting system performance.

## Introduction

Exposure to fine particulate matter (PM_2.5_) is a major environmental health risk globally, resulting in approximately 4.1 million deaths annually.^1^ About 75% of all attributable gross external PM_2.5_ damages^2^ occurs in just four sectors, 21% of which is due to the transportation sector. Despite a decline in recent years, the transportation sector’s total gross external damages still range between $52B and $120B/year in the United States^3^ and is the second most significant sector contributing to PM_2.5_ exposure-related deaths in the United States (30,000 deaths annually).^4,5^ Major improvements in the design and management of transportation systems are required to mitigate exposure from vehicle-based emissions,^6^ especially given the high intake fraction (*iF*) of transportation sources relative to other systems.^7^ The *iF* of an emission source represents its exposure efficiency, which quantifies the total inhalation intake of a pollutant that would take place per unit of emissions.^8^ We have previously established how exposure to primary and secondary PM_2.5_ from modeled vehicle flow emissions can be quantified and mitigated through exposure-based vehicle rerouting using a traffic assignment (TA) model,^9^ which is a model used to estimate traffic flow distribution on a network based on a desired objective function measure to be optimized.^10^ This work builds upon Bin Thaneya et al.^9^ by further investigating exposure-based routing trends on a larger network with more comprehensive modeling inputs, as well as utilizing the model for exploring other strategies to mitigate PM_2.5_ exposure from on-road vehicle emissions.

The contributions of this work can be better highlighted by presenting a summary of related past works, which can be split into two main themes: (1) environmental TA models and (2) PM_2.5_ exposure assessments of transportation systems. Various environmental TA models with objectives ranging from minimizing fuel use, greenhouse gas (GHG) emissions, and other pollutant emissions have been developed in the literature.^11^ To the authors’ knowledge, few TA models account for pollutant concentration and none have accounted for pollutant exposure. There is growing evidence suggesting that trade-offs exist between travel time and certain environmental objectives.^11−13^ TAs can be used to assess the degree of this trade-off for different networks and determine what feasible interventions would be most beneficial in each specific case. Past studies have focused on developing the mathematical formulations of TAs, assessing practical ways for their implementation, and analyzing the network effects of environmental TA models that aim to minimize fuel consumption,^14−16^ criteria air pollutant emissions^15,17−20^ or concentrations,^21^ GHG emissions,^15,18^ or a combination of these objectives.^16,22,23^ The TA model formulated in Bin Thaneya et al.^9^ and used in this work builds upon previous models by developing a novel TA objective function that routes traffic based on primary and secondary PM_2.5_ exposure.

The literature on traffic-related pollutant exposure is extensive and spans a multitude of analysis levels ranging from local to global exposure scales. A significant portion of the literature uses *iF* and intake metrics to study the exposure efficiency and exposure burden of traffic emissions.^7,24−34^ These studies show the importance of developing highly spatially resolved air quality models or high-resolution monitoring networks that can capture the intraurban variation of exposure concentrations and reduce uncertainties in exposures required for epidemiology studies.^34,35^ Studies also address different aspects that can impact an exposure assessment, including accounting for daily mobility in exposure studies^36^ or self-exposure from vehicles during daily travel,^37^ assessing the effects of urban population characteristics (e.g., population density and urban form) and land area on exposure to vehicle emissions,^38^ exploring different combinations of vehicle fuel technologies for climate and health effects,^39−42^ and assessing the potential of operational control strategies (i.e., vehicle scheduling and route assignment optimization) as a complement to capital control strategies (i.e., investing in a new vehicle fleet) for reducing climate and health impacts from emissions of public transportation.^43^ The equity and environmental justice implications of PM_2.5_ and other pollutant exposure have also been a major topic of interest, where studies have shown that a large exposure burden is carried by minority and low-income groups from transportation system emissions.^33,44−47^

The main contributions of this work build upon Bin Thaneya et al.^9^ by further investigating how transportation network systems affect overall baseline exposure trends. Specifically, the effects of network congestion (by modeling peak and off-peak traffic flow), different vehicle user classes, public transportation, and electricity generation for transportation purposes are explored for two model runs: (1) a user-equilibrium for travel time (UET) scenario and (2) a system optimal for PM_2.5_ intake (SOI) scenario. The UET represents the baseline scenario where network travelers minimize their own individual travel time, while the SOI represents the exposure-based routing scenario that minimizes overall PM_2.5_ damages. The other major contribution is quantifying and comparing the mitigation potential of different transportation-based exposure reduction strategies. The novelty of this approach is using emission inventories based on modeled traffic flows rooted in realistic system behavior as opposed to measured or observed emission inventories. This allows the exposure mitigation potential of different strategies to be systematically quantified relative to baseline levels, and a portfolio of effective exposure interventions can therefore be identified and adopted by transportation system planners and policymakers. The different transportation-based PM_2.5_ reduction strategies explored include: (1) assuming future vehicle fleet mixes in present-day conditions, (2) reducing personal PM_2.5_ exposure through particle filtration, (3) increasing public transportation use, (4) controlling the time and travel path of heavy-duty truck (HT) trips to minimize their exposure contributions, and (5) developing a bi-objective optimization framework that balances travel time with PM_2.5_ exposure.

## Materials and Methods

The exposure-based TA modeling framework described in Bin Thaneya et al.^9^ is used to inform the transportation-related exposure trends and strategies explored herein. A more comprehensive set of inputs is introduced such that the model can be run with higher fidelity and obtain more representative results and trends that can help design effective exposure reduction strategies. The network case study models traffic flow in the Chicago Metropolitan Area (CMA). The input sets include (1) a larger exposure domain, which spans Illinois and its 10 nearby states, (2) a higher-resolution network with roadway-specific volume-delay functions, (3) an expanded origin-destination (O–D) matrix disaggregated by trip purpose, vehicle class, and time-of-day (TOD) period, (4) public transportation use including bus and light-rail travel, and (5) electricity generation related to plug-in electric vehicle (EV) charging and light-rail use. The spatially distributed exposure impacts due to traffic flow emissions from the CMA are estimated using results from a baseline UET run and an SOI run, which represents the first PM_2.5_ exposure reduction strategy. The mathematical formulations of the UET and SOI follow those in Bin Thaneya et al.,^9^ with the only major difference being the travel time functions used. Once baseline trends of the expanded network are obtained, the aforementioned PM_2.5_ mitigation strategies are introduced at different stages of the modeling framework.

### Network Description and Inputs

The expanded CMA network is adapted from the Chicago Metropolitan Agency for Planning (CMAP) data hub.^48^ A summary of network data and properties as well as the trip demand data will be presented next. Details can be found in the Supporting Information (SI). Data and assumptions for the upcoming subsections follow the methodology outlined in CMAP Travel Demand Model Documentation.^49^

#### Roadway and Public Transit Network Description

**Figure 1:**
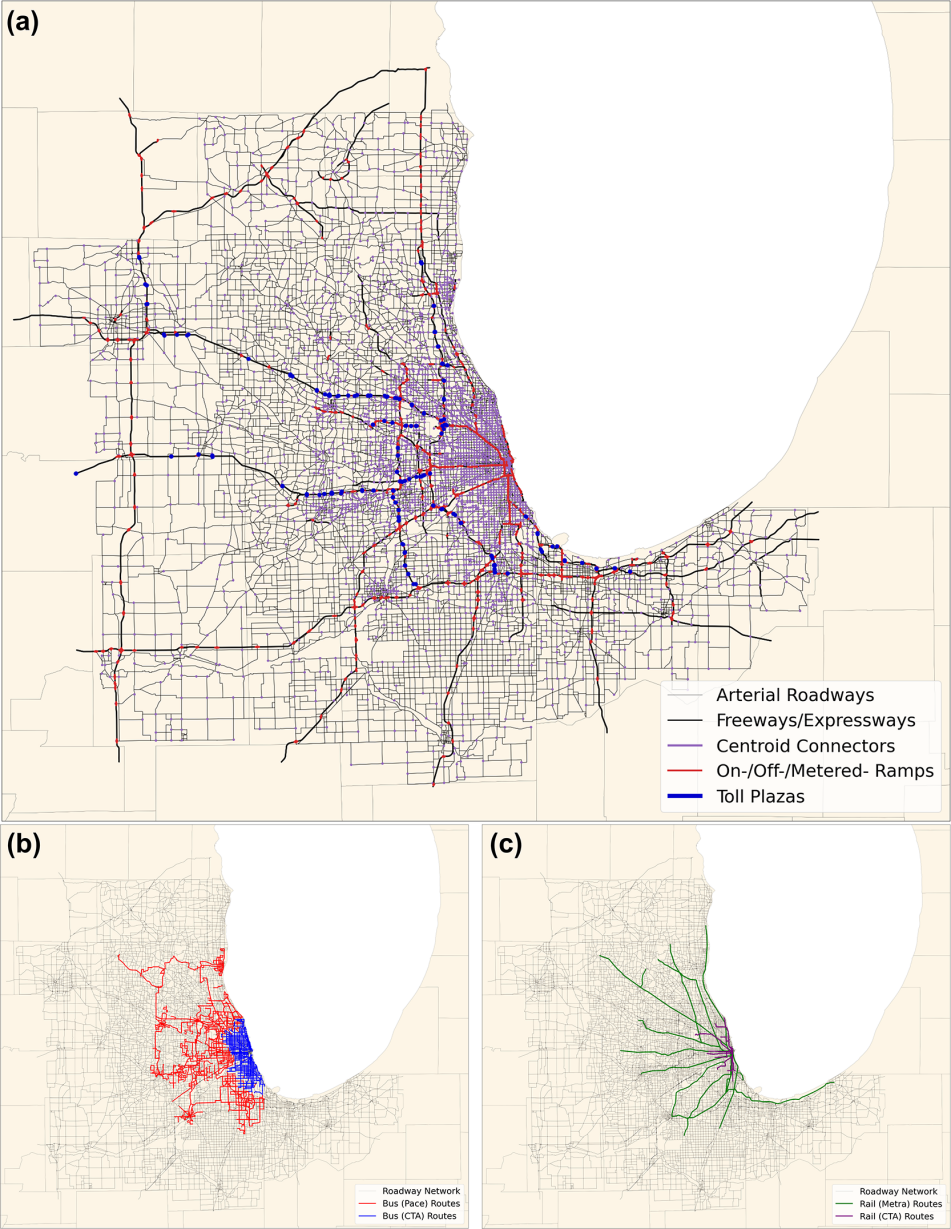
(a) Map showing the Chicago Metropolitan Agency for Planning (CMAP) transportation network. The network is made up of ∼55,000 links representing different roadway types including arterial roadways ending in signalized intersections, freeways/expressways, zone centroid connectors, freeway ramps, and toll plazas. Zone centroids are nodes placed throughout the network that represent an aggregated trip generation zone. Zone centroid connectors are artificial links that connect the zones to the rest of the network. For the purposes of this study, they are assumed to represent arterial streets. (b) Map showing the different bus routes in the CMAP transportation network. (c) Map showing the different light-rail routes in the Chicago Metropolitan Area (CMA). The CMA’s public transportation systems are provided by three public operating agencies which include: the Chicago Transit Authority (CTA), Metra commuter rail, and Pace suburban bus with extended services through various parts of the CMA.

Figure 1 displays the expanded CMA network composed of 55,000 links and 20,000 nodes. It shows the different roadways represented within the network. These include arterial streets, freeways/expressways, zone centroid connectors, toll plazas, and various ramps connecting the different road segments. The general attributes provided for all roadways include link free-flow speed, free-flow travel time, link length, link capacity, and time-varying toll costs (if present). Arterial roadways also have additional parameters (green time and green time-to-cycle length ratios) for estimating idle time due to delay at signalized intersections. Detailed volume-delay functions for each roadway type and tolling estimate calculations can be found in the SI.

Bus and light-rail travel are the two public transit modes modeled within the study. The bus and light-rail routes are shown in Figure 1b and Figure 1c, respectively. Unlike other on-road vehicle trip paths, which are determined using the TA model, the bus and rail routes are scheduled and fixed. Bus and rail flow on network links is calculated based on the average headway for that specific route. Per Figure 1b, the bus system uses the same network links; therefore, bus routes are pre-coded in the TA model and will affect the assignment process by increasing vehicle flow on the links on their routes. The TA algorithm loads the bus flow on the used link routes for that time period prior to the assignment process for the remaining trips, using the same volume-delay functions prescribed for the other on-road vehicles. Light-rail trips occur on a separate rail network independent of the vehicle network and do not affect vehicle travel. Data from Chester and Horvath^50^ are used to determine light-rail travel speed and electricity consumption, which are used to calculate overall rail travel time and associated emissions.

#### Trip Demand Data

The expanded CMAP data also provide a variety of trip types disaggregated by trip purpose and vehicle user type, including light-duty (LDV) and heavy-duty (HDV) vehicles. CMAP data further provide TOD factors for each trip purpose which allow the disaggregation of vehicle trips by 8 TOD periods. The 8 TOD periods are defined as follows:

- TOD 1: The ten-hour late evening-early morning off-peak period (8:00 p.m. to 6:00 a.m.);
- TOD 2: The shoulder hour preceding the AM peak hour (6:00 to 7:00 a.m.);
- TOD 3: The two-hour peak AM period (7:00 to 9:00 a.m.);
- TOD 4: The shoulder hour following the AM peak period (9:00 to 10:00 a.m.);
- TOD 5: The four-hour midday period (10:00 a.m. to 2:00 p.m.);
- TOD 6: The two-hour shoulder period preceding the PM peak period (2:00 to 4:00 p.m.);
- TOD 7: The two-hour peak PM period (4:00 to 6:00 p.m.);
- TOD 8: The two-hour shoulder period following the PM peak period (6:00 to 8:00 p.m.).

This finer temporal resolution allows for better capturing of congestion trends during peak hours. Being able to model congestion periods during peak hours also allows for modeling higher peak hour emissions and obtaining more representative PM_2.5_ exposure profiles. Given the 8 TOD periods, the TA model would need to be run 8 times to model a single day of vehicle travel on the network. Overall, the trip demand data represent around ∼25 million daily vehicle trips within the region (∼30 million including public transit trips). Other details regarding trip demand data can be found in the SI.

### Emissions and Exposure Modeling

The vehicle emissions framework follows the methodology described in Bin Thaneya et al.,^9^ which accounts for both primary and secondary PM_2.5_ emissions from precursor emissions including nitrogen oxides (NO_*x*_), sulfur oxides (SO_*x*_), volatile organic compounds (VOCs), and ammonia (NH_3_). Vehicle emission rates are estimated using the EMission FACtor (EMFAC) average-speed, static-emissions model.^51^ The expanded vehicle trip data and network allow for developing emission functions that are specific to the different vehicle user classes and roadways modeled. Idle emissions due to delay at signalized intersections are also captured using EMFAC data. The vehicle emissions inventory, which is input into the air quality model, as described later, is quantified using link vehicle flows, average speeds, and congestion rates that are derived from the TA runs. Details regarding vehicle emissions modeling can be found in the SI.

Emissions from electricity generation due to EV charging and light-rail use are also accounted for. For EV use, it is assumed that a proportion of the vehicle fleet is composed of plug-in EVs. EV fleet proportions are based on EMFAC data.^51^ Charging habit data (i.e., typical charging session time and duration) are then used to calculate charging energy requirements per each hour of the day.^52^ Remaining details regarding how the electricity demand from EV charging is quantified and accounted for in the TA model can be found in the SI. Energy consumption due to light-rail use for public transport is quantified based on daily light-rail route data. Data regarding light-rail energy consumption per vehicle miles traveled are sourced from Chester and Horvath.^50^ Once the electricity requirements of EV charging and light-rail use are quantified, the increase in electricity demand is added to the daily baseline demand for the region. The exposure-based optimal power flow (OPF) model, described in detail in Bin Thaneya and Horvath,^53^ is then used to estimate emissions and exposure resulting from electricity generation unit (EGU) activity required to meet electricity demand in the CMA region.

PM_2.5_ concentrations from vehicle and EGU emissions are estimated using the Intervention Model for Air Pollution (InMAP)^54^ Source-Receptor Matrix (ISRM),^55^ which holds linearized emission-concentration relationships developed from InMAP, a reduced-complexity air quality model. The ISRM is adopted since running a TA model and a chemical transport model (CTM) concurrently would be computationally expensive. An added benefit of the ISRM is its variable grid resolution, which becomes progressively finer in high-density areas, making it ideal for exposure assessment studies. The modeling of finer pollutant concentration gradients in areas with large population numbers, while being computationally efficient in capturing long-range transport and secondary formation of PM_2.5_, makes the ISRM ideal for exposure assessment and analyzing mitigation scenarios. The ISRM is augmented with population^56^ and breathing rate^57^ data to transform the ISRM from linearized emission-concentration relationships to emission–intake relationships that account for population distribution effects in relation to exposure. PM_2.5_ exposure and exposure damages modeling are then calculated using concentration–response functions^45,58^ in addition to the value of statistical life (VSL) metric.^59^ The damages modeling methods are summarized in the SI and closely follow the methods outlined in detail in Bin Thaneya et al.^9^ and Bin Thaneya and Horvath.^53^ All exposure damages and cost estimates are adjusted to 2019 dollars.

### PM_2.5_ Exposure Mitigation Strategies

Table 1 shows a summary of all PM_2.5_ strategies analyzed in this study. All PM_2.5_ exposure reductions are compared against the baseline UET scenario. The first strategy involves the SOI optimization that assigns traffic routes in the network in a manner that minimizes human PM_2.5_ intake. The first of the remaining five strategies assumes accelerating the adoption of future vehicle fleet mixes into the current CMA fleet. Projections of the potential future fleet mixes were obtained from EMFAC.^51^ Three different scenarios are modeled assuming mixes for the years 2030, 2040, and 2050. The fleets are adopted while holding all other present-day baseline conditions, meaning that flow assignments for the UET are unaltered. The OPF model is run assuming both current base grid (BG) conditions with the present EGU network and a future grid (FG) scenario corresponding to each of the three future vehicle fleet years. FG scenarios assume a higher adoption of renewable generation, which are based on Illinois’ Renewable Portfolio Standards (RPS) that assume 25% and 50% renewable generation by 2026 and 2040, respectively.^60^ We develop a third hypothetical scenario that assumes 75% renewable generation by 2050 to model the effects of aggressive renewable adoption. The FG scenarios are described in detail in Bin Thaneya and Horvath.^53^ The second strategy assesses PM_2.5_ personal exposure reductions using high-efficiency particulate arrestance (HEPA) filtration within households in high-exposure census tracts. Exposure reductions are applied to census tracts in the top 50th, 75th, and 90th percentile of damages in the baseline run using commercially available high-efficiency (HE: true-HEPA) or low-efficiency (LE: HEPA-type) filtration devices with exposure reduction potentials based on Maestas et al.^61^

**Table 1:** List of All Strategies Analyzed and Their Corresponding Descriptions

strategy code	description
UET	Baseline user-equilibrium for travel time minimizing individual travel time.
Pareto (90:10)	Bi-objective strategy minimizing both individual travel time (90% weight) and PM_2.5_ exposure (10% weight).
Pareto (75:25)	Bi-objective strategy minimizing both individual travel time (75% weight) and PM_2.5_ exposure (25% weight).
Pareto (50:50)	B-iobjective strategy minimizing both individual travel time (50% weight) and PM_2.5_ exposure (50% weight).
Pareto (25:75)	Bi-objective strategy minimizing both individual travel time (25% weight) and PM_2.5_ exposure (75% weight).
Pareto (10:90)	Bi-objective strategy minimizing both individual travel time (10% weight) and PM_2.5_ exposure (90% weight).
SOI	System optimal for intake minimizing PM_2.5_ exposure.
FLT30 (BG)	2030 vehicle fleet scenario assuming base grid conditions.
FLT30 (FG)	2030 vehicle fleet scenario assuming future 2030 grid conditions.
FLT40 (BG)	2040 vehicle fleet scenario assuming base grid conditions.
FLT40 (FG)	2040 vehicle fleet scenario assuming future 2040 grid conditions.
FLT50 (BG)	2050 vehicle fleet scenario assuming base grid conditions.
FLT50 (FG)	2050 vehicle fleet scenario assuming future 2050 grid conditions.
LE FILT90	Particle filtration scenario assuming low-efficiency HEPA filtration adoption in census tracts in the top 90th percentile of damages.
HE FILT90	Particle filtration scenario assuming high-efficiency HEPA filtration adoption in census tracts in the top 90th percentile of damages.
LE FILT75	Particle filtration scenario assuming low-efficiency HEPA filtration adoption in census tracts in the top 75th percentile of damages.
HE FILT75	Particle filtration scenario assuming high-efficiency HEPA filtration adoption in census tracts in the top 75th percentile of damages.
LE FILT50	Particle filtration scenario assuming low-efficiency HEPA filtration adoption in census tracts in the top 50th percentile of damages.
HE FILT50	Particle filtration scenario assuming high-efficiency HEPA filtration adoption in census tracts in the top 50th percentile of damages.
PUB05	5% increase in public transportation use.
PUB10	10% increase in public transportation use.
PUB20	20% increase in public transportation use.
PUB40	40% increase in public transportation use.
Rerouting Trucks	Moving 75% of all truck trips to the low-congestion overnight period (TOD 1) and applying PM_2.5_ exposure-based routing.

The next strategy involves changes in the baseline mode split share, where an increase in public transportation for daily trips is assumed using both bus and light-rail transport. A 5%, 10%, 20%, and 40% increase in public transportation use is analyzed, replacing individual LDV trips in all cases.

The final two strategies involve applying a modified version of the SOI. The base SOI optimization assumes that all vehicle trips can be controlled, and vehicles can be routed in a manner that minimizes PM_2.5_ intake. Applying vehicle routing at such a level may not be entirely feasible for personal vehicles, especially since most travelers favor routes with minimal travel time. However, targeting truck-based trips with SOI-based routing control could be more efficacious, especially through policy mechanisms aimed at reducing the overall pollution impacts of trucks. The highest SOI reductions are achieved when congestion conditions are low, allowing more opportunity to reroute traffic to low-*iF* links without any overload that leads to high emissions. The lowest congestion conditions occurred during the overnight period (TOD 1). Thus, this strategy assumes that truck-based trips are moved from other TODs to TOD 1 such that 75% of all truck-based trips take place during this time frame where they follow an SOI-based routing principle.

Trade-offs between travel time and PM_2.5_ exposure exist as exhibited by the UET and SOI assignment results.^9^ However, the UET and SOI objective functions can be combined to generate a set of trade-off optimal solutions known as Pareto-optimal solutions. This new formulation can be solved using a weighted-sum method, where both objective functions are merged into a single formulation by multiplying each by a weight, forming a convex combination of objectives.^62−65^ Weights are parametrically varied to obtain a Pareto front. Since a large magnitude difference exists between the travel costs and exposure damages (travel costs are valued 10 times higher in this instance, as shown later), each objective is normalized by the interval of its variation over the Pareto-optimal set. Using a bi-objective approach can help reduce flow on the most damaging links without incurring excessively large travel times. This can be practically implemented using a first-best pricing scheme where road tolls can be set such that they reflect the external exposure cost generated by each traveler.^19^ The derivation of the bi-objective formulation as well as details regarding the other strategies can be found in the SI.

## Results

### General Trends from the UET and SOI Assignments

#### Travel Time and Network Congestion

**Figure 2:**
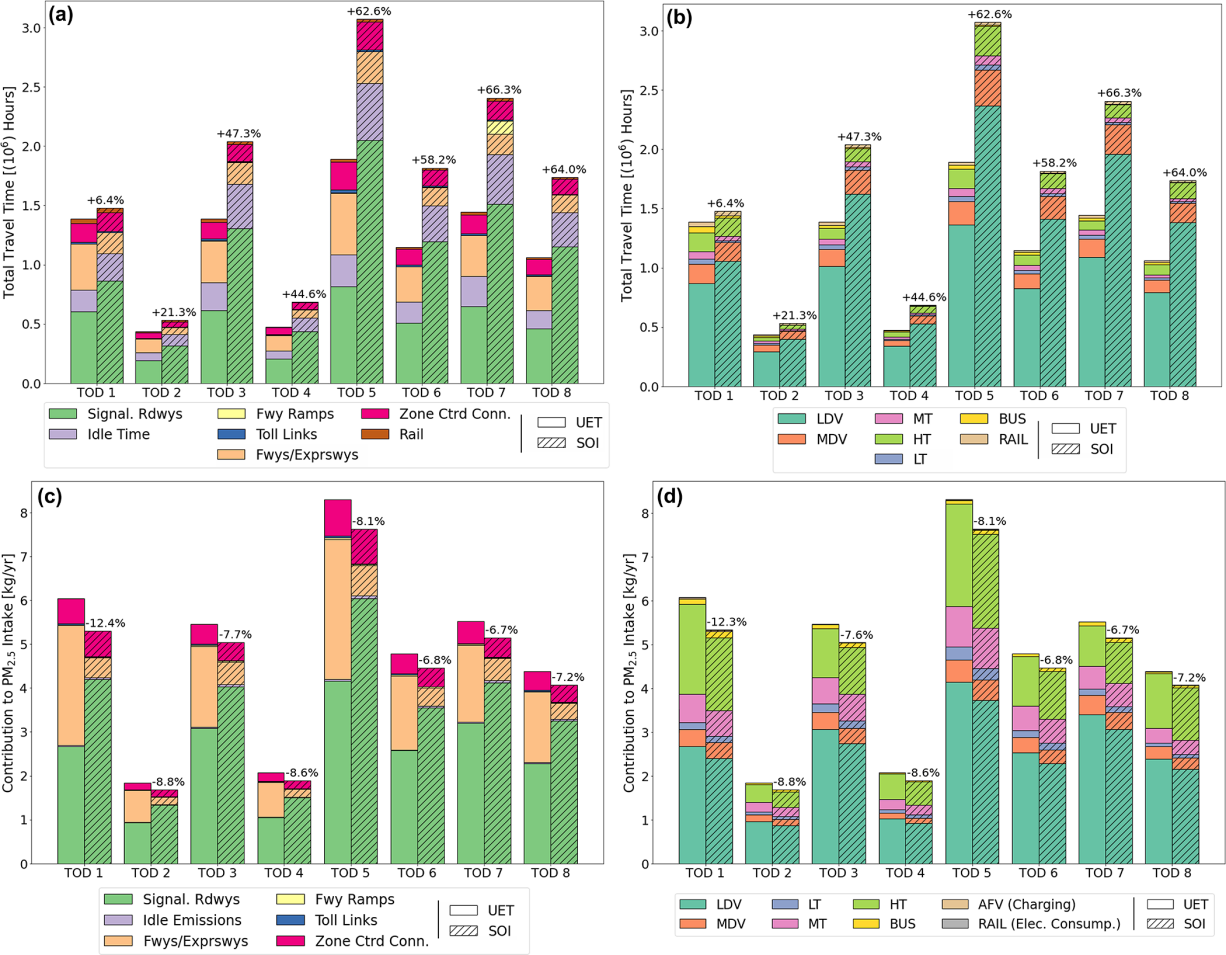
(a) User-equilibrium for time (UET) and system optimal for intake (SOI) passenger travel time hours disaggregated by time-of-day (TOD) period and roadways utilized. (b) UET and SOI passenger travel time hours disaggregated by TOD period and vehicle user class. (c) UET and SOI PM_2.5_ intake disaggregated by TOD period and roadways utilized. (d) UET and SOI PM_2.5_ intake disaggregated by TOD period and vehicle user class. Percent changes show differences in values in the SOI relative to the UET. (LDV: light-duty vehicles; MDV: medium-duty vehicles; LT: light-duty trucks; MT: medium-duty trucks; HT: heavy-duty trucks).

The SOI leads to higher vehicle delay and overall travel time, especially during peak hours where the magnitude of delay in the SOI assignment can be 2–3 times that of the UET. The increase in network congestion and delay caused by the SOI assignment is largely due to differences in network flow between both assignments, which are dictated by their respective routing principles. The SOI overloads links with low *iF*s to reduce overall PM_2.5_ exposure without any congestion considerations unless that leads to increased exposure. Figures S13–S20 in the SI plot links with increased vehicle flow in each of the UET (subplot (a)) and SOI (subplot (b)) relative to the other assignment for all TOD periods. They show that the SOI assignment reroutes flow away from the high-*iF* links (especially freeways/expressways) located in the higher-population-density Chicago urban center and onto the low-*iF* links (mostly arterial roadways) on the CMA outskirts. Figure 2a shows travel time disaggregated by TOD and link type utilized for both assignments. High utilization of local roadways also leads to a large increase in idle time in the SOI, which is 65% higher than idle time in UET. The UET leads to 3.0 billion travel hours per year, while the SOI leads to 5.0 billion travel hours per year (+66%). An alternative measure for understanding travel time differences between both scenarios is vehicle delay, which is the difference between actual travel time and free-flow travel time (i.e., travel time when no congestion is present) on network links. When analyzing delay times for the most utilized links for both scenarios, the 50th percentile UET link delay times are around 15 (off-peak)–30 (peak) s, but 99th percentile link delay times can be as high as 1.5 (off-peak)–3 (peak) min for highly congested links. The most extreme cases in the UET show link delay times as high as 20–40 min during peak hours. The SOI shows much larger delay times, with link delay times at the 50th and 99th being 25 (off-peak)–55 (peak) s and 5.0 (off-peak)–13 (peak) min, respectively. Extreme cases in the SOI show link delay times of 45 min–2 h, which is due to high vehicle rerouting to links with extremely low *iFs.* When accounting for the value of time^49,66^ of the different user classes (shown in Table S2 in the SI), the monetized travel time costs of the UET and SOI are $53B and $88B/year, respectively. Figure 2a also shows that the largest increase in travel time occurs during peak hours (∼60%), while the smallest increase occurs during the overnight off-peak period, TOD 1 (∼6%). Figure 2b plots travel time by vehicle type and transportation mode. Across the UET and SOI assignments, LDVs make up the majority of travel time (71% and 78%, respectively), followed by MDVs and trucks (24% and 20%, respectively), and last by public transport (4.0% and 1.5%, respectively).

#### PM_2.5_ Exposure

The SOI assignment leads to higher overall emissions for each of the five pollutants, to varying degrees, relative to the UET due to the longer trips created by its rerouting principle. Emissions become especially high during peak hours. Despite the higher emissions, the SOI reduces overall PM_2.5_ exposure due to its higher utilization of low-*iF* links. The mechanism in which the SOI reduces overall exposure through choosing low-*iF* links is explained in detail in Bin Thaneya et al.^9^ and summarized in the SI. PM_2.5_ intake disaggregated by type of roadways traveled on is plotted in Figure 2c. Most intake is attributed to travel on arterial roadways in the UET (50%) and SOI (80%) assignments, closely followed by travel on freeways and expressways (40% and 10%, respectively). The remaining 10% is split among travel ramps, centroid connectors, and idle emissions.

Figure 2d plots intake contribution disaggregated by vehicle type as well as EGUs due to EV charging and rail use. The split in intake distribution varies by TOD period but is similar for both assignments. LDVs (77% of all trips) lead to the most PM_2.5_ intake (52%) which is highest during peak hours and is lowest during the overnight period. Trucks are the next largest contributors to intake. Their induced intake is the highest during the overnight period (TOD 1) and the midday period (TOD 5) when most truck trips occur. Due to their higher relative emissions, they contribute to 38% of all intake despite comprising 11% of all trips. Public transportation is attributed with 1.5% of PM_2.5_ intake mostly due to bus travel (1.4%). This is because vehicle *iF*s are higher than those of EGUs. Intake contributions from EGUs due to EV charging and rail use are about 0.2%.

In terms of congestion effects on intake reduction, the highest reduction in intake is achieved during off-peak periods (8%–12%). Reductions are smaller during peak hours (∼7%), which shows that higher congestion and link saturation levels limit the SOI’s ability to further reduce PM_2.5_ intake through strategic rerouting.

The UET and SOI assignments lead to a total PM_2.5_ intake of 39 and 35 kgPM_2.5_/year (−8.3%), respectively, which reduces to 36 and 33 kgPM_2.5_/year (−9.6%) when analyzing the CMA only. Total exposure damages range between $3.7B–$8.3B/year and $3.4B–$7.6B/year (−8.2%), for the UET and SOI, respectively. Approximately 10% of exposure due to vehicle flow within the CMA occurs beyond the CMA’s domain.

**Figure 3:**
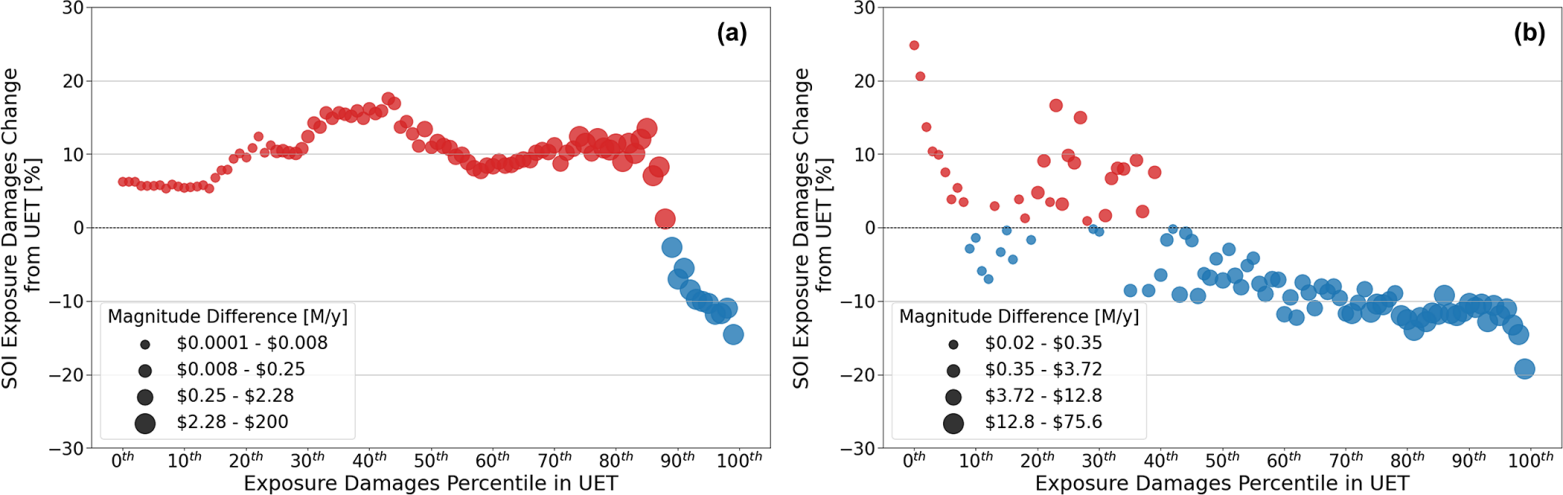
Relative and absolute change in PM_2.5_ exposure damages [$M/year] between the user-equilibrium for time (UET) and system optimal for intake (SOI) for different percentile rankings of population subgroups in the (a) entire exposure domain and (b) Chicago Metropolitan Area (CMA). Percentiles are based on baseline exposure damages in the UET.

Figure 3 plots the relative and absolute changes in exposure reduction between the UET and SOI for different population subgroups. Populations are grouped according to their baseline damages percentile in the UET. Figure 3a plots damages for the whole exposure domain, while Figure 3b shows exposure damages for the CMA only. While most of the percentile groups in Figure 3a see an increase in damages due to the SOI rerouting, the magnitude of damages is small relative to the reductions achieved. The percentile groups that experience the largest damage differential are those that reside within the CMA (plotted in Figure 3b). In the CMA, the amount and magnitude of exposure increase are smaller than the reduction achieved. The SOI reduces exposure damages the most for the populations that experience the highest level of damages in the baseline scenario. While the overall reduction in damages is about 8.2%, the most impacted groups see reductions on the order of 10%–20%, with the highest damages group (∼260,000 people) benefiting from a $76M reduction in damages annually.

### Other PM_2.5_ Reduction Strategies

**Figure 4:**
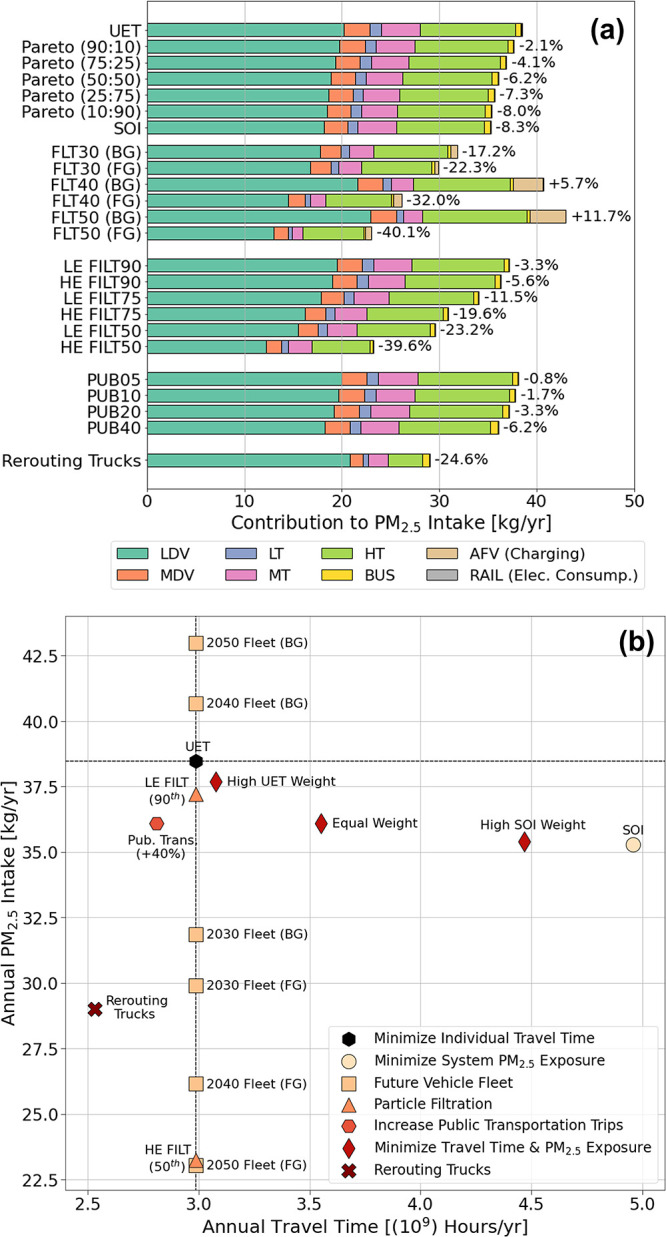
(a) PM_2.5_ intake disaggregated by vehicle user class for the different strategies and scenarios assessed. Percent changes show differences in intake for the strategies relative to the user-equilibrium for time (UET). (b) Scatter plot showing PM_2.5_ intake and annual passenger travel time hours for the different scenarios and strategies assessed. Only select representative cases of the strategies were plotted. Dotted vertical and horizontal lines intersect the UET baseline scenario. (LDV: Light-duty vehicles; MDV: Medium-duty vehicles; LT: Light-duty trucks; MT: Medium-duty trucks; HT: Heavy-duty trucks).

Figure 4a plots the annual PM_2.5_ intake for all strategies explored disaggregated by vehicle user class. Summary results for all strategies are also tabulated in Table S4 in the SI. The relative differences in PM_2.5_ intake are compared against the baseline UET scenario. The future vehicle fleet scenarios are designated as *FLTxx (BG/FG)* in Figure 4a, where *xx* corresponds to the year of the future fleet mix (*30:2030; 40:2040; 50:2050*). Each year is analyzed assuming electric power under BG or FG conditions. Electricity generation increases by 25 TWh (+15%), 80 TWh (+45%), and 100 TWh (+60%) due to additional EV charging for the 2030, 2040, and 2050 scenarios, respectively. PM_2.5_ intake increases or decreases, relative to baseline conditions, highly dependent on the grid mix used for charging EVs. Assuming present BG conditions, the 2030 scenario leads to a reduction in PM_2.5_ intake (17%), whereas 2040 and 2050 scenarios lead to a 5.7% and 12% increase in PM_2.5_ intake, respectively. This is due to the increase in EGU SO_*x*_ (2040: +590% and 2050: +760%) and primary PM_2.5_ (2040: +110% and 2050: +150%) emissions, from baseline combined EGU and vehicle emissions, which do not offset the reduction in intake brought by forgone vehicle-based emissions (mostly NO_*x*_). EGU emission increases are due to the additional electricity demand being met by fossil-fuel-based EGUs (i.e., coal, oil, and some natural gas) since the current grid is not set up to handle such an increase through renewables. Assuming FG conditions, as outlined in Bin Thaneya and Horvath,^53^ renewable generation will increase to 25%, 50%, and 75% in the three respective scenarios, leading to a 22%, 32%, and 40% reduction in PM_2.5_ intake, respectively, which is among the highest reductions achieved by all strategies.

The particle filtration strategies are designated as *(LE/HE) FILTxx* in Figure 4a, where *xx* corresponds to the damage percentile of population subgroups in the base scenario that are provided with LE or HE HEPA filters. Only including the 90th percentile of the highest damaged tracts reduces intake by 3.3% and 5.6% for LE and HE filtration, respectively. If the inclusion criterion is expanded and set to the 75th and 50th percentiles, then intake reductions are approximately 12% (LE) and 20% (HE); and 23% (LE) and 40% (HE), respectively. Switching from LE filtration to HE filtration almost doubles the magnitude of intake reduction. Using census housing data^56^ as well as information on how the filters were utilized within each household outlined in Maestas et al.,^61^ the capital costs required to purchase and supply the filters were estimated. The capital costs for the scenarios range between $1.0B and $16B, but the total reduction potential in damages only ranges between $0.27B and $3.2B/year. Therefore, if only considered for transportation-based PM_2.5_, the benefits of the filters may not outweigh their overall costs.

The public transportation strategies are designated as *PUBxx* in Figure 4a, where *xx* represents the increase in public transportation use relative to baseline conditions. PM_2.5_ intake is reduced by 0.80%–6.2% when public transportation trips increase by 5%–40%. Reductions are limited because public transportation makes up a small percentage of all vehicle trips within the network (1%). A major increase in public transportation use would be required to reduce the intake further. The mean *iF*s of the links traveled by replaced LDVs for the five pollutants are 35%–70% higher than the mean *iF*s of the remaining network links. Thus, a reduction in flow within that general area would lead to considerable reductions if enough vehicles were replaced, showing the potential that this strategy could have.

The truck-routing strategy assumes that 75% of all truck trips are moved to TOD 1, where they have pre-planned exposure-reduction routes. Baseline results showed that trucks contribute to 38% of all intake while only comprising 11% of all trips due to their high emissions. The trips were chosen to take place during TOD 1 since that is the period of day in which the SOI was able to reduce intake the most due to a lack of network congestion. By utilizing this routing strategy, truck contributions to intake increase by ∼15% during TOD 1; however, the eliminated truck trips in the remaining TODs reduce truck contributions to intake by ∼70%. The reduced congestion from eliminated truck trips also reduces other vehicle intake contributions by an additional ∼5%. Applying SOI-based rerouting to trucks leads to an overall emissions reduction for all pollutants by 13%–26% and PM_2.5_ intake by 25%.

The bi-objective optimization strategy is designated as *Pareto(xx:yy)*, where *xx* and *yy* correspond to the relative weight given to the UET and SOI normalized objective functions, respectively. The weights enable policymakers to choose the relative importance of each objective. The largest increase in reductions is obtained as the SOI weights are initially increased, but reductions diminish as the SOI assignment is approached. This is because the initial reductions reduce flow from the highest-*iF* links and yield the highest reductions in exposure impacts. A practical implementation of this bi-objective-based routing can be established through road tolls that shift traffic away from high-*iF* links. The relative weighting given to the SOI can be used to determine both the magnitude of the tolls and the links to which they are applied to. Figures S38–S42 in the SI show how tolls can be applied to different vehicle categories, assuming the full exposure externality is to be internalized by the vehicle users. Tolls increase for higher polluting vehicle categories and are the highest for HTs. The tolls also reflect congestion effects that increase toll costs due to the higher induced emissions. HT tolls range between $0.22 and $4.0 during off-peak hours but can be as high as $1.4 −$30 during congestion periods. Figures S38–S42 show that there is a correlation between links with no tolls and links to which much of the traffic flow was rerouted to in the SOI (as seen in Figures S13–S20). Conversely, the high-toll links were the ones from which the SOI rerouted traffic flow away from.

Figure 4b plots annual travel time against PM_2.5_ intake of the UET and mitigation strategies. The future vehicle fleet and particle filtration strategies lead to no flow changes relative to the UET. Optimizing for PM_2.5_ exposure leads to the highest increase in network travel time (+66%). The degree of travel time increase in the bi-objective formulation is tied to the relative weights of both objective functions. When the UET objective is weighted higher, the increase in travel time for the two weighting schemes chosen is between 3.1% and 8.3%. Their corresponding reduction in PM_2.5_ exposure is 2.1% and 4.1%. Weighting both objective functions equally leads to a 19% increase in travel time and a 6.2% reduction in PM_2.5_ exposure. When the SOI objective function is weighted higher, the increase in travel time is 33% and 50% for the two weighting schemes assessed, while their corresponding reduction in exposure is 7.3% and 8.0%. As the weights became higher for the SOI objective function, the rate at which exposure is reduced diminishes, while the rate at which travel time increases accelerates. Increasing public transportation use reduces both travel time and PM_2.5_ exposure. Travel time is reduced by relieving congestion due to forgone LDV trips. The overall reduction in travel time ranges between 0.77% and 5.9% for a 5–40% increase in public transportation trips. Moving most truck trips to TOD 1 and applying exposure-based routing not only reduces PM_2.5_ exposure but also relieves network congestion. Given that trucks are modeled as 2- and 3-vehicle equivalents, their absence from the network allows for much faster travel for the remaining LDVs. Overall, this strategy leads to a 15% reduction in network travel time.

## Discussion

The baseline UET scenario shows that PM_2.5_ exposure damages from transportation use are on the order of $3.7B–$8.3B/year, with LDVs causing about 50% of all damages. Trucks are attributed with 38% of all exposure damages, despite only making up 11% of all trips, due to their relatively high emissions.^67,68^ Public transportation exposure contributions are small (1.4%) due to low ridership compared to other vehicle trip types. Similarly, baseline EGU-based exposure from EV vehicle charging and light-rail use is low (0.2%).

The first strategy explored is exposure-based rerouting, which yields considerable reductions in exposure by shifting traffic flow away from high-*iF* links and high-population-density areas. The use of the expanded network showed how the SOI utilizes low-*iF* arterial roadways on the CMA outskirts, as opposed to freeways/expressways located near high-population-density tracts, for most traffic flow. Despite the small increase in idling emissions and exposure from utilizing these types of roadways, overall exposure impacts are reduced by about 8.2%, which is in line with performances of TA optimization models that minimize other environmental objectives.^11^ The use of the expanded trip demand table also helped reveal how network congestion and travel demand in the Chicago urban center inhibit further reductions to exposure using the SOI. Exposure reductions were smaller during peak traffic hours (∼7%) relative to those during off-peak hours (∼12%). The SOI can take advantage of underutilized roadway capacity during off-peak hours to shift traffic flow onto low-*iF* links without creating excess congestion and emissions (or other unintended consequences such as rapid pavement deterioration^69^). The high amount of traffic flow during peak hours saturates roadways at a faster rate, which leads to higher congestion-related emissions. Despite the SOI’s efforts to reroute traffic away from the Chicago urban center, demand constraints will still cause some flow on high-*iF* roadways in those areas, especially during peak traffic hours when much of the travel demand begins (p.m.) and/or ends (a.m.) in the Chicago urban center. SOI routing trends found here are in line with other environmental-based TA studies. Although the environmental objectives were different, Ahn and Rakha^23^ also found that shifting routes through arterial roadways over freeways/expressways helped reduce different pollutant emissions and fuel consumption. Benedek and Rilett^20^ also showed that system optimal (SO) assignments that minimize emissions achieve low reductions when networks are near saturation and limited alternative routes are available. Despite the comparatively lower overall reduction in exposure, the benefits of the SOI are highlighted when looking at the most impacted populations which see the highest exposure damage reductions (10%–20%). The population subgroup in the 99th percentile of damages (∼260,000 people) obtain an exposure damages reduction of $76M/year.

Despite the benefits of the SOI, challenges remain in its implementation. Enforcing vehicles to follow exposure-based routing willingly at the expense of their travel time is practically difficult to apply. Furthermore, the SOI does not account for travel time when assigning vehicle flows, which results in the 66% increase in travel costs observed. Due to the large difference in magnitude between the monetized travel costs and exposure damages (travel costs are valued 10 times higher in this instance), attempting to develop an objective function that concurrently minimizes monetized travel costs and exposure damages would heavily weigh the travel costs. This approach would also assume that monetized travel costs and health damages are equivalent. Instead, a normalized weighted-sum approach of both objective functions can help policymakers weigh each respective goal based on their own valuation. The bi-objective optimization formulation resulted in a Pareto frontier that balances travel costs and exposure damages, and initial reductions in the damages from the UET come with small increases in travel time (3.1%–8.3% increase in travel time for a 2.1%–4.1% reduction in exposure damages). The initial exposure reductions target only the heaviest damaging links, which is why travel time does not drastically increase. Flow shifts away from high damaging links can be achieved using exposure-based tolling that can disincentivize network users from traveling on these links. Exposure tolls have been developed for different vehicle classes and roadway congestion conditions since emissions and the induced exposures from both of those factors heavily influence the value of the tolls, which as shown can be up to $4 and $30 during off-peak and peak hours, respectively. The use of roadway tolls has been shown to be effective in reducing congestion in high-traffic areas, especially during peak traffic hours, which can effectively lower measured PM_2.5_ and ultrafine particle concentrations in downwind locations.^70^ Thus, real-world application of exposure-based routing either through a full SOI-like implementation or simply targeting high-exposure-inducing roadways could be achieved with exposure-based tolling.

Another more applicable use of exposure-based routing from a policy perspective is targeting truck-based trips. It is a more efficient approach given that trucks form a smaller proportion of all trips (11%) but are responsible for a high proportion of all exposure damages (38%). Shifting truck trips to the overnight period also takes advantage of low-congestion conditions, which gives the TA more options for routing when reducing exposure. Overall, this policy not only reduces overall exposure by 25% but also relieves congestion in the remaining TODs, achieving a 15% reduction in network travel time. With the rise in autonomous truck driving and platooning, applying exposure-based routing systems to trucks is an implementable goal.^71,72^

Another strategy that leads to reductions in both travel time and exposure is the increase in public transportation use. However, both reductions are relatively small and will not be substantial unless a large increase in public transit use is achieved (a 40% increase in transit trips leads to a 6% reduction in exposure and travel time). This is mainly because transit trips do not form a large fraction of the overall network trips (∼1%). The relatively modest increases in public transportation use modeled are based on declining public transit ridership in the United States, where bus and light-rail ridership declined by 15% and 3%, respectively, between 2012 and 2018.^73^ Even increases in public transportation ridership in different transit markets, such as those in Seattle and New York, only saw a 20%–30% increase between 2006 and 2016.^74^ A more ambitious increase in public transportation use such as a doubling in transit ridership would lead to 14% and 13% reduction in PM_2.5_ exposure and systemwide travel time, respectively. An added benefit of this strategy is that most public transit trips are concentrated within high-population-density urban city centers, so replaced vehicle trips and emissions occur in areas with higher exposure reduction potential.^75^ However, an issue with bus emissions is that even though each bus is assumed to replace ∼60 vehicle trips, bus NO_*x*_ and primary PM_2.5_ emissions are approximately 100 and 25 times larger than emissions from LDVs.^51^ Therefore, even higher reductions could be achieved if alternative-fuel (e.g., CNG or hydrogen fuel cell) buses were used.

An analyzed “end-use” strategy is reducing personal PM_2.5_ exposure through particle filtration. Results show that considerable reductions in exposure (up to 40%) can be achieved even when only targeting the subset of census tracts in the most impacted areas. However, this strategy incurs high capital costs (up to $16B) compared to the potential magnitude of reductions from transportation exposure that can be obtained ($1.9B–$3.2B/year). This does not account for the exposure damages reductions from other polluting sources that these filters can help target. Average PM_2.5_ concentrations due to all emission sources would need an average ∼5 μg/m^3^ to result in damages high enough to offset the capital costs of the filters. While a particle filtration strategy could help offer an immediate response to high PM_2.5_ exposure, it only masks the actual pollution without eliminating it. Thus, promoting such a strategy could discourage emissions control and PM_2.5_ concentration reduction.

Another critical finding of this work is showing how accounting for EGU-based emissions resulting from electric energy use in transportation systems is essential for capturing the full impact of the sector. Although baseline exposure due to electricity generation is small (<1%) compared to vehicle-induced exposure, the increased penetration of EVs in future fleet mixes would make EGU-based exposure of high importance. Estimates show that EV electric energy demand can add 16%, 47%, and 61% to baseline energy demand when assuming a 2030, 2040, and 2050 vehicle fleet, respectively. The status of the electricity grid mix determines whether exposure is reduced (up to 40%) or increased (up to 12%) due to high EV penetration. High electrification with energy supplied from the current Illinois grid mix would lead to an increase in exposure due to the high presence of fossil-based electricity generation. This is in agreement with past studies which showed that vehicle electrification powered by high shares of coal within the electricity mix could increase health damages 1.8–6.3 times.^39,40^ Furthermore, full electrification does not eliminate all emissions from vehicles as primary PM_2.5_ will still be emitted from brake wear and tire wear.^47^ Primary PM_2.5_ intake from brake wear and tire wear make up approximately 20% of all vehicle PM_2.5_ exposure in the baseline scenario, meaning that some vehicle exposure would remain even if full electrification were achieved. While vehicle electrification may seem the best solution in the long term, the turnover rate for EV adoption,^76^ grid upgrades needed for handling the increase in electricity demand from EV adoption,^77,78^ and the requirement that the electricity grid mirrors the elimination of combustion-based sources for this strategy to be effective^39,40^ may signal that time is needed for electrification’s reduction in exposure to become fully effective.

The other aforementioned strategy limitations show that no one strategy alone is the solution to transportation-based PM_2.5_ exposure. Thus, from a policy perspective, some combination of different strategies would be the best path forward. A combined approach when running multiple strategies collectively achieves reductions upwards of 50%. It should also be noted that interactions between the different strategies could either reinforce or dampen their overall mitigation effects. An example of a reinforcing interaction is that higher toll costs for individual travelers may prompt higher public transportation use instead of choosing a low-exposure toll-free travel path, thus increasing the mitigation potential of both strategies. On the other hand, a high penetration of EVs would mean that exposure-based tolls would not be as effective given that a high proportion of vehicles would be exempt from being tolled. Interactions between different mitigation policies require further assessment and should be prioritized in future work. The effects of these strategies on the formation of other potential pollutants should also be noted. Given that the modeled strategies would cause a large reduction in vehicle NO_*x*_ emissions, an increase in ozone (O_3_) concentrations may result due to O_3_ formation in urban areas being VOC-limited.^79^

The data sources and modeling framework introduce several sources of uncertainty into the study. Uncertainty in emissions and concentration modeling as well as the underlying limitations of the TA model have been discussed extensively in Bin Thaneya et al.,^9^ including those related to the linearization of emission–concentration relationships in the ISRM. Excluding nonlinear relationships of NO_*x*_ and NH_3_ transformations to particulate nitrate and ammonium may overestimate PM_2.5_ concentration contributions from these species, leading to an overestimation of overall exposure damages.^80^ Similarly, limitations of the exposure-based OPF are discussed extensively in Bin Thaneya and Horvath.^53^ One of the TA limitations included not capturing the effects of off-peak and peak traffic conditions as well as vehicle idling effects on exposure. While an aspect of those limitations was handled using the TOD travel demand disaggregation, not all features of a full dynamic TA are captured. These include more comprehensive vehicle emissions due to dynamics such as acceleration and deceleration as well as the additional congestion effects created by link spillover and queuing.^81^ The TA also only models one representative day of traffic flow and extrapolates those results for the whole year. On average, this may be sufficient for the study goals since chronic exposure trends are being modeled; however, capturing weekday/weekend traffic effects and days of extremely high and low traffic could be of interest to future exposure assessments, especially if such a framework were to be applied to other pollutants such as O_3_, which is more subject to weekday/weekend effects.^82^ Another limitation of the model is that no restrictions are placed on the types of roadways that different vehicle classes are typically allowed to travel on in both the UET and SOI scenarios. This means that the different truck classes may be traveling on restricted arterial roadways in order to reduce their individual travel time or induced exposure, which may affect both the baseline travel time and exposure results as well as the overall reductions achieved from the different rerouting strategies.

There is uncertainty in the analyzed strategies as well. The future fleet strategy projects a certain vehicle fleet mix to be present within future years; however, any projections regarding future vehicle technology are never entirely accurate. The assumptions of personal exposure reduction established by Maestas et al.^61^ may not be representative of all subjects within this exposure domain. Another limitation of this approach is that the ISRM measures outdoor exposure concentrations while the use of particle filtration devices reduces indoor personal exposure concentrations. The findings in Maestas et al.^61^ were chosen to be applied to the filtration strategies herein because the study directly measures reductions to personal PM_2.5_ exposure concentrations (by equipping study participants with battery-powered particulate monitors) as opposed to indoor and/or outdoor concentrations and using that as a proxy for personal exposure concentrations. Given that the reported reductions are applied directly to exposure concentrations, and exposure concentration (and subsequently PM_2.5_ inhalation intake) is the main factor leading to exposure health damages, it is assumed that the exposure reductions Maestas et al.^61^ find from using particle filters translate directly to reductions in PM_2.5_ intake and exposure damages. Furthermore, a major limitation of the exposure assessment is that it does not account for people’s daily mobility patterns and assumes that all exposure takes place in their residence, an element that was shown to introduce inaccuracies in exposure assessments.^36^ Average bus and rail ridership data were used to develop the public transit replacement rate for LDVs. Ridership values span a large range, especially for peak and off-peak hours; therefore, the replacement rates assumed may not be fully representative and may overestimate the reductions achieved.^50^ The modeling framework does not account for diurnal pollutant dynamics and seasonal differences in pollutant emission trends and meteorology. Sathaye et al.^83^ show that nighttime freight trips may lead to higher pollutant concentrations relative to daytime trips due to the atmospheric boundary layer being more stable during the night. Thus, vehicle nighttime emissions may increase daily average pollutant concentrations. As such, the nighttime truck rerouting strategy may be overestimating the actual reductions in exposure. However, since the rerouting strategy also employs exposure-based routing, the concentration increases due to the nighttime shift should occur in regions with low-exposure potential. Overall, despite the modeling limitations from the uncertainty sources, the exposure results are still within previously published figures. Specifically, the range of PM_2.5_ exposure concentrations for the UET scenario within the CMA (0.01–2.0 μg/m^3^) is comparable to the population-weighted PM_2.5_ concentration due to all California on-road mobile sources (∼1.6 μg/m^3^) estimated in Apte et al.^84^ The UET’s per-capita induced mortality rate in the CMA ranges between 2.6 and 6.9 deaths per 10^5^ people per year, which is within the same order of magnitude as the normalized deaths reported by Thakrar et al.^4^ (∼6 deaths per 10^5^ people per year) and Apte et al.^58^ (∼33 deaths per 10^5^ people per year) attributable to transportation- and all-source-related PM_2.5_ exposure, respectively, in the United States.

Results show how the modeling framework can be used to develop insights regarding transportation systems management and PM_2.5_ exposure mitigation. It further showed the importance of quantifying the effects of internal transportation system factors such as congestion, idling, and public transportation, as well as external factors such as exposure due to electricity generation. Overall, exposure trends should be derived from realistic system behavior, and mitigation estimates must be based on a systematic quantification of exposure reductions from baseline levels. Future work should also focus on integrating transportation infrastructure contributions to PM_2.5_ intake in this type of framework. Greer et al.^47^ showed that infrastructure emissions from pavement maintenance and resurfacing activities can contribute up to 1.5 kgPM_2.5_ intake per year for a given transportation network, which is about 4% of the total annual intake found in this study. An added application of this modeling framework that is yet to be utilized is to inform the design of future transportation networks and systems. Reductions to PM_2.5_ exposure were limited due to network constraints and travel demand requirements. Accounting for exposure reductions and link *iF* values in the future design of networks could help limit exposure greatly. Furthermore, exposure-based routing may have implications beyond PM_2.5_ exposure such as noise,^85,86^ driver and pedestrian safety,^87,88^ and roadway infrastructure^89−92^ impacts, especially if vehicles (more so HDVs) are routed to roadways with low *iF*s that may not be equipped to handle high-traffic volumes. The development of multicriteria objective functions that account for impacts beyond exposure and travel time and can be integrated into these types of modeling frameworks is essential. Finally, the implication of vehicle flow rerouting on the exposure of different demographic subgroups is an aspect that requires further assessment. Rerouting measures or any other strategy implementations should not come at the expense of increasing the exposure disparity among marginalized population subgroups. Upon assessing the exposure impacts of the SOI relative to the UET of different demographic and income groups within this study, results show that the population-weighted PM_2.5_ concentrations are reduced for all demographic (White: −8.0%; Black: −16%; Asian: −13%; non-White Hispanic: −10%; Other: −11%) and income groups (Q1 [lowest income quintile]: −17%; Q2: −11%; Q3: −9.1%; Q4: −7.5%, Q5 [highest income quintile]: −9.9%) to varying degrees without disadvantaging one group over the other. The SOI also reduces the exposure disparity of some subgroups upon comparing their population-weighted concentrations to the mean population-weighted concentration (e.g., the Black subgroup population-weighted concentration is 6.2% higher than the mean in the UET, but 0.85% lower than the mean in the SOI; the Asian population-weighted concentration is 39% higher than the mean in the UET and 34% higher than the mean in the SOI). However, it does increase the exposure difference slightly for some subgroups (e.g., the White subgroup goes from being 14% lower than the mean in UET to being 12% lower than the mean in the SOI). The demographic exposure results are specific to this case, with no guarantee that future applications of this model to other domains will yield these types of exposure benefits among different subgroups. Therefore, a future iteration of this model should incorporate an equity component that aims to reduce the exposure disparities between different population subgroups in addition to overall PM_2.5_ exposure.

## Supporting Information Available

Network volume delay functions, additional trip demand data, additional emissions and exposure modeling data description, detailed PM_2.5_ reductions strategies description, supplementary travel time and congestion results, supplementary emissions and exposure results, and tabulated summary results (PDF)
