# Supplementary material for: Exploring Regional Reduction Pathways for Human Exposure to Fine Particulate Matter (PM2.5) Using a Traffic Assignment Model
Source: Environ Sci Technol. 2023 Nov 13;57(48):19649–62. doi: 10.1021/acs.est.3c05594 (PMC10702527; doi:10.1021/acs.est.3c05594)
Supplement: Supplementary file 1 — es3c05594_si_001.pdf [file es3c05594_si_001.pdf]

Exploring Regional Reduction Pathways for Human Exposure to Fine Particulate Matter ($PM_{2.5}$) using a Traffic Assignment Model

(Supporting Information)

Ahmad Bin Thaneya[†*] *and Arpad Horvath*[†]

[†]Department of Civil and Environmental Engineering, University of California, Berkeley, California, 94720, United States

Pages: 75

Figures: 42

Tables: 4

Table S1 List of abbreviations used in the main text and SI and their corresponding descriptions.

ABBREVIATION	DESCRIPTION
**BG**	Base Grid
**CMA**	Chicago Metropolitan Area
**CMAP**	Chicago Metropolitan Agency for Planning
**CTA**	Chicago Transit Authority
**EGU**	Electricity Generation Unit
**EMFAC**	EMission FACtor Model
**EV**	Electric Vehicle
**FG**	Future Grid
**GHG**	Greenhouse Gas
**HE**	true-HEPA
**HEPA**	High-Efficiency Particulate Arrestance
**HT**	Heavy-Duty Trucks
**IF**	Intake Fraction
**INMAP**	Intervention Model for Air Pollution
ISRM	InMAP Source-Receptor Matrix
**LDV**	Light-Duty Vehicles
**LE**	HEPA-type
**LT**	Light-Duty Trucks
**MDV**	Medium-Duty Vehicles
**MT**	Medium-Duty Trucks
**NH3**	Ammonia
**NOX**	Nitrogen Oxides
**O-D**	Origin-Destination
**OPF**	Optimal Power Flow
**PM2.5**	Fine Particulate Matter
**RPS**	Renewable Portfolio Standards
**SOI**	System Optimal for Intake
**SOX**	Sulfur Oxides
**TA**	Traffic Assignment
**TOD**	Time-of-Day
**UET**	User-Equilibrium for Travel Time
**VOCS**	Volatile Organic Compounds

1. Supplementary Material and Methods

1.1 Network Volume Delay Functions

The following data, assumptions, and network equations are adapted from the methodology outlined in CMAP Travel Demand Model Documentation.[1] As opposed to the simplified network in Bin Thaneya et al.[2], roadway specific volume-delay functions are used to model travel time on the different roadway types. **(1.1)** shows the general Bureau of Public Roads (BPR) function that is used to estimate travel time between intersections in arterial roadways as well as freeway exit ramps that lead into arterial roadways. $\boldsymbol{t_{ij}}$ and $\boldsymbol{t_{ij}^0}$ are the total travel time and free-flow travel time on $\boldsymbol{link}$ $(\boldsymbol{i},\boldsymbol{j})$ in units of hours. $\boldsymbol{x_{ij}}$ is the optimization variable which represents total vehicle flow within a given analysis period, and $\boldsymbol{u_{ij}}$ is the capacity of the link. The general link capacity is assumed to be at level of service C, which is 75% of the capacity at level of service E. $\boldsymbol{\alpha_{art}}$ and $\boldsymbol{\beta_{art}}$ are unitless calibration parameters, which are fixed at 0.15 and 4, respectively, for arterial roadways and ramps. Functions for estimating uniform and incremental intersection delay time (resulting in idle time) are shown in **(1.2)** and **(1.3)**, respectively. They are regression-based equations used for calculating signal delay for different signal cycle length and green time-to-cycle length ratios.[3] $\boldsymbol{UD_{ij}}$ and $\boldsymbol{ID_{ij}}$ are the average uniform intersection delay and the incremental intersection delay at the $\boldsymbol{j}$ node of $\boldsymbol{link}(\boldsymbol{i},\boldsymbol{j})$, respectively. Both delay times are restricted to positive values and converted to hours to match the travel time in the rest of the network. $\boldsymbol{g_j}$ represents the green time allowed at the link's $\boldsymbol{j}$-node intersection, while $\boldsymbol{c_j}$ is the cycle length ratio at the intersection. The combined maximum intersection delay length is restricted to one cycle length. Travel time on toll plazas and centroid connectors assume the typical volume-delay function shown in **(1.1)**.

$$t_{ij}(\boldsymbol{x_{ij}}) = t_{ij}^{0}\left(1 + \alpha_{art}\left(\frac{\boldsymbol{x_{ij}}}{u_{ij}}\right)^{\beta_{art}}\right) \qquad (\mathbf{1.1})$$

$$UD_{ij}(\boldsymbol{x_{ij}}) = 6.0\left(\frac{\boldsymbol{x_{ij}}}{u_{ij}}\right) - 0.39g_j + 0.35c_j - 4.5 \qquad (\mathbf{1.2})$$

$$ID_{ij}(\boldsymbol{x_{ij}}) = 2.7\left(\frac{\boldsymbol{x_{ij}}}{u_{ij}}\right)^{8} - 7.3\left(\frac{g_j}{c_j}\right) + 3.4 \qquad (\mathbf{1.3})$$

The next set of volume-delay functions, shown in **(2.1)** and **(2.2)** represent travel time on freeways, expressways, and freeway-freeway ramps. BPR functions for freeways/expressways and their associated ramps are adjusted to reflect how the marginal travel time on these types of links rapidly increases once their practical capacity is exceeded (i.e., once volume-capacity ratios become greater than one). This is property is reflected in the functional form of the conditional volume-delay equations used and by setting $\boldsymbol{\beta_{fwy}}$ to a higher power (8), which exponentiates the travel time at a higher rate once capacity is exceeded. $\boldsymbol{\alpha_{fwy}}$ remain at 0.15. Furthermore, a 15% reduction in travel time is applied to uncongested freeway/expressway links to reflect drivers' tendencies to exceed speed limits when traffic volumes are low.

$$t_{ij}(\boldsymbol{x_{ij}}) = \frac{t_{ij}^{0}}{1.15}\left(1 + \alpha_{fwy}\left(\frac{\boldsymbol{x_{ij}}}{u_{ij}}\right)\right)\left(1 + \alpha_{fwy}\left(\frac{\boldsymbol{x_{ij}}}{u_{ij}}\right)^{\beta_{fwy}}\right) \quad ,for \quad \left(\frac{\boldsymbol{x_{ij}}}{u_{ij}}\right) \leq 1. \qquad (\mathbf{2.1})$$

$$t_{ij}(\boldsymbol{x_{ij}}) = t_{ij}^{0}\left(1 + \alpha_{fwy}\left(\frac{\boldsymbol{x_{ij}}}{u_{ij}}\right)^{\beta_{fwy}}\right) \quad ,for \quad \left(\frac{\boldsymbol{x_{ij}}}{u_{ij}}\right) > 1. \qquad (\mathbf{2.2})$$

Another issue with using a generic BPR volume-delay function for all roadway types is the inability to restrict flow on freeway on-ramps during peak-traffic hours, which end up unrealistically over-assigning freeways/expressways during congested peak periods. The modified freeway/express volume-delay functions restrict some of this over-assignment. Additionally, metered on-ramps are assigned a higher power degree ($\boldsymbol{\beta_{ramp}} = 10$) and given a

maximum flow rate capacity ($\boldsymbol{u_{meter}}$ = 720 vehicles per lane per hour) as shown in **(3)** to deter from over-assigning to freeways/expressways.

$$t_{ij}(\boldsymbol{x_{ij}}) = t_{ij}^{0}\left(1 + \alpha_{ramp}\left(\frac{\boldsymbol{x_{ij}}}{u_{meter}}\right)^{\beta_{ramp}}\right) \qquad (\mathbf{3})$$

As mentioned, travel time at tolling plazas is calculated using the base volume-delay function shown in **(1.1)**. However, toll plaza links within the network are coded as 200 feet long, meaning that the actual travel time on them is negligible within the context of the rest of the network. Instead, a travel-cost tolling scheme is developed to capture a users' willingness to travel through tolling plazas given the toll charge. The toll cost is translated to a travel time to be minimized using three factors: (1) the monetary toll cost on the toll plaza, (2) the user classes' value of time, and (3) the user classes' perception factor. Perception factors modify the user's value of time to include other decision-making considerations that may influence a user's decision to travel through the tolling plaza. **(4)** shows how a toll amount on a toll plaza link is converted into an equivalent travel time ($\boldsymbol{t_{ij-y}}$) for a specific user class ($\boldsymbol{y}$) on toll plaza ($\boldsymbol{i,j}$). $\boldsymbol{toll_{ij-y}}$ represents the dollar cost of passing through toll plaza ($\boldsymbol{i,j}$) for user class ($\boldsymbol{y}$). $\boldsymbol{VOT_y}$ and $\boldsymbol{PF_y}$ represent the value of time [$/h] and perception factor [-] of user class ($\boldsymbol{y}$), respectively. A higher perception factor lowers the users' perceived toll costs and impedance of travelling through the plaza. The value of time and perception factors of different user classes is shown in **Table S2.** Value of time figures and perception factors are all sourced from CMAP Travel Demand Model Documentation.[1] The values of time used are comparable to the recommended value of travel time savings (~$25/hr)[4] used by the Department of Transportation for all cost-benefit analysis related to transportation-related projects or infrastructure planning.

Table S2 Value of time [$/h] and perception factors of different vehicle user classes.

User Class	Value of Time [$/h]	Perception Factor
**HWB low income (SOV)**	12	9
**HWB high income (SOV)**	20	9
**HWB low income (HOV2)**	18	9
**HWB high income (HOV2)**	30	9
**HWB low income (HOV3+)**	27	9
**HWB high income (HOV3+)**	45	9
**HBO (SOV)**	12	8
**HBO (HOV2)**	18	8
**HBO (HOV3+)**	27	8
**NHB (SOV)**	12	8
**NHB (HOV2)**	18	8
**NHB (HOV3+)**	27	8
**B-Plate truck**	20	2
**Light truck**	25	2
**Medium truck**	25	2
**Heavy truck**	40	2
**External Auto**	18	3
**Air Traveler**	30	3

$$t_{ij-y} = \frac{toll_{ij-y}}{VOT_y \times PF_y} \qquad (\mathbf{4})$$

1.2 Trip Demand Data

The different types of trips and vehicle types that are accounted for include:

1. Light-duty vehicle trips which are further disaggregated by trip purpose:
    a. Home-based work trips (HBW) traveling as single occupancy vehicles or high occupancy vehicles with 2 or 3 occupants;
    b. Home-based other trips (HBO) traveling as single occupancy vehicles or high occupancy vehicles with 2 or 3 occupants;
    c. Non-home-based other trips (NHB) traveling as single occupancy vehicles or high occupancy vehicles with 2 or 3 occupants;
2. B-plate truck trips;
3. Light-duty truck trips;
4. Medium-duty truck trips;
5. Heavy-duty truck trips;
6. Auto point-of-entry (POE) trips which are external light-duty vehicle trips that are entering the CMA on major expressways;
7. Truck point-of-entry (POE) trips which are external heavy-duty vehicle trips that are entering the CMA on major expressways; and
8. Airport trips

To better model the effects of buses and trucks on travel time on links, medium-duty trucks are assumed to be 2 vehicle-equivalents, while buses and heavy-duty trucks are assumed to represent 3 vehicle-equivalents. Light-duty vehicle trips are given in the form of Production-Attraction (P-A) matrices (i.e., trips have no directional meaning), while the TA requires all trip demand data to be in O-D matrices (i.e., trips have directional meaning). Thus, some data handling is required to transform CMAP trip demand data into a form that can be input into the TA model. **Figure S1(a)** shows vehicle trips disaggregated by trip purpose and TOD. **Figure S1(b)** and **(c)** show vehicle trips disaggregated by trip purpose and TOD, respectively.

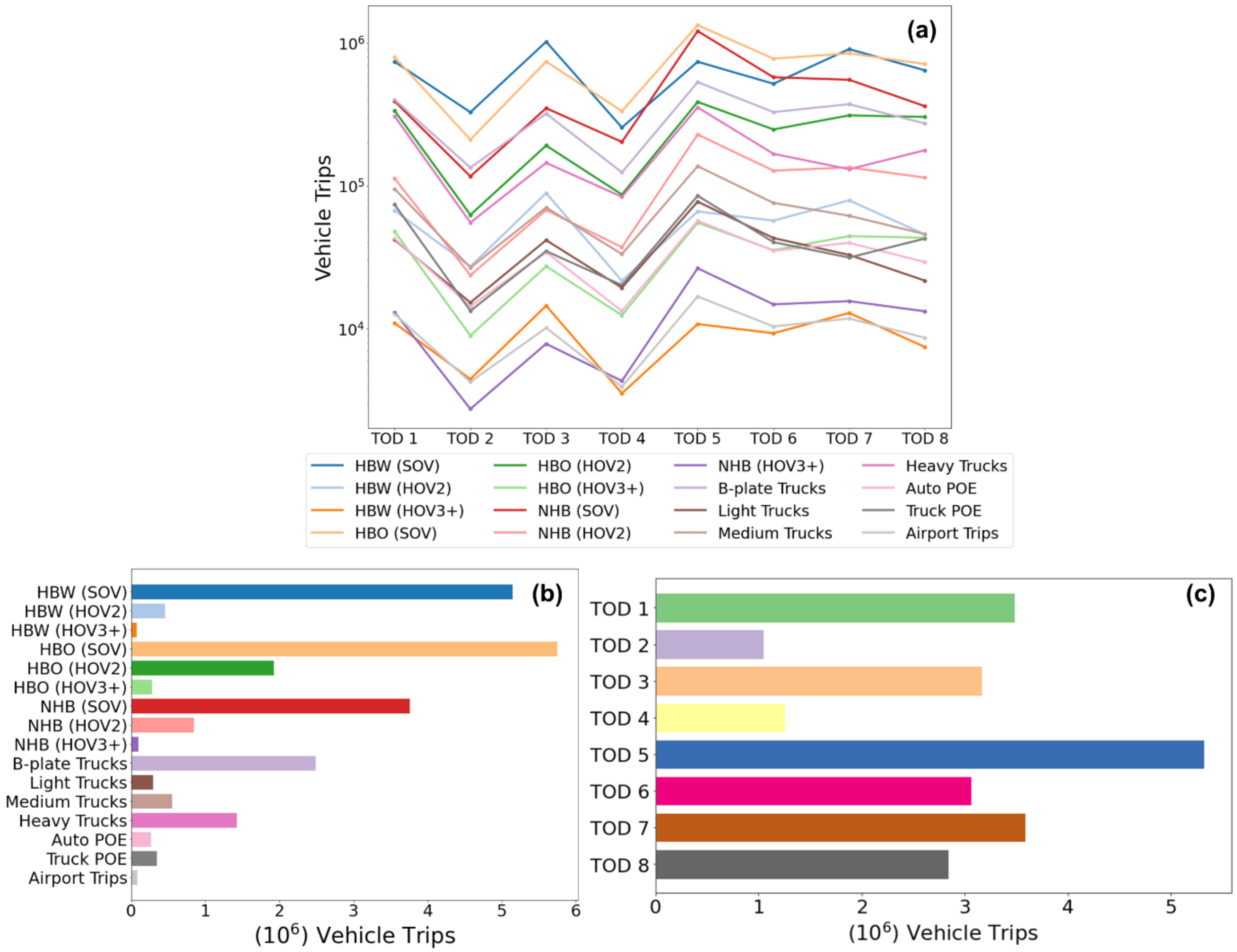

Figure S1: (a) Trip demand disaggregated by time-of-day (TOD) period and trip purpose. (b) Trip demand disaggregated by trip purpose only. (c) Trip demand disaggregated by TOD period. (HWB: Home-based work; HBO: Home-based other; NHB: Non-home based; SOV: Single-occupancy vehicle; HOV: High-occupancy vehicle; POE: Point-of-entry). (TOD 1: The ten-hour late evening-early morning off-peak period (8:00 p.m. to 6:00 a.m.); TOD 2: The shoulder hour preceding the AM peak hour (6:00 to 7:00 a.m.); TOD 3: The AM peak two hours (7:00 to 9:00 a.m.); TOD 4: The shoulder hour following the AM peak period (9:00 to 10:00 a.m.); TOD 5: A four-hour midday period (10:00 a.m. to 2:00 p.m.); TOD 6: The two-hour shoulder period

preceding the PM peak period (2:00 to 4:00 p.m.); TOD 7: The PM peak two hours (4:00 to 6:00 p.m.); TOD 8: The two-hour shoulder period following the PM peak period (6:00 to 8:00 p.m.)).

1.3 Emissions and Exposure Modeling

EMFAC offers emission rates of various vehicle classes which were linked to the different vehicle trips described in the previous subsection. **Table S3** shows vehicle user classes and their corresponding EMFAC classifications. The developed emission functions also account for the type of roadway the vehicles are traveling on, specifically with respect to their volume-delay functions since vehicle speed affects the amount of emissions that would occur. This leads to some slight modification of the emission functional form used in the SOI, but the rest of the formulation remains the same. Emission rate curves and EMFAC data points for primary $PM_{2.5}$ (tailpipe emissions as well as brake ware and tire ware emissions), NO_x, VOCs, SO_x, and NH_3 for the different vehicle classes driving on arterial roadways are plotted in **Figures S2 – S6**. The emission rates were plotted as functions of the link flow-to-capacity ratio for varying link FFS. As expected, higher emissions are found for the larger trucks. Generating NH_3 emission functions for the different vehicle classes did not yield a smooth continuous function for all, so a smoothed weighted average NH_3 emission function was assumed for all vehicle classes. In addition to moving emission, idle emissions due to delay at signalized intersections were modeled using EMFAC data for the different vehicle classes. Idle emissions in units of [g/min] for the different vehicle types is plotted in **Figure S7**. Idle emissions for NH_3 were not provided by EMFAC. Heavy-duty trucks emit the largest amount of pollutants when idling. Idle emissions are most significant for NO_x and VOCs.

Table S3 Vehicle user classes and their corresponding EMFAC class.

User Class	EMFAC Class	EMFAC Description
**LDV**	LDA	Passenger Cars
	LDT1	Light-Duty Trucks (GVWR* <6000 lbs and ETW** <= 3750 lbs)
	LDT2	Light-Duty Trucks (GVWR <6000 lbs and ETW 3751-5750 lbs)
**MDV (B-Plate)**	MDV	Medium-Duty Trucks (GVWR 5751-8500 lbs)
**LT**	LHD1	Light-Heavy-Duty Trucks (GVWR 8501-10000 lbs)
	LHD2	Light-Heavy-Duty Trucks (GVWR 10001-14000 lbs)
	T6 Public Class 4	Medium-Heavy Duty Public Fleet Truck (GVWR 14001-16000 lbs)
	T6 Public Class 5	Medium-Heavy Duty Public Fleet Truck (GVWR 16001-19500 lbs)
	T6 Public Class 6	Medium-Heavy Duty Public Fleet Truck (GVWR 19501-26000 lbs)
	T6 Utility Class 5	Medium-Heavy Duty Utility Fleet Truck (GVWR 16001-19500 lbs)
	T6 Utility Class 6	Medium-Heavy Duty Utility Fleet Truck (GVWR 19501-26000 lbs)
	T6 Instate Tractor Class 6	Medium-Heavy Duty Tractor Truck (GVWR 19501-26000 lbs)
	T6 Instate Delivery Class 4	Medium-Heavy Duty Delivery Truck (GVWR 14001-16000 lbs)
	T6 Instate Delivery Class 5	Medium-Heavy Duty Delivery Truck (GVWR 16001-19500 lbs)
	T6 Instate Delivery Class 6	Medium-Heavy Duty Delivery Truck (GVWR 19501-26000 lbs)
	T6 Instate Other Class 4	Medium-Heavy Duty Other Truck (GVWR 14001-16000 lbs)
	T6 Instate Other Class 5	Medium-Heavy Duty Other Truck (GVWR 16001-19500 lbs)
	T6 Instate Other Class 6	Medium-Heavy Duty Other Truck (GVWR 19501-26000 lbs)
	T6 CAIRP Class 4	Medium-Heavy Duty CA International Registration Plan Truck (GVWR 1400116000 lbs)
	T6 CAIRP Class 5	Medium-Heavy Duty CA International Registration Plan Truck (GVWR 1600119500 lbs)

	T6 CAIRP Class 6	Medium-Heavy Duty CA International Registration Plan Truck (GVWR 1950126000 lbs)
	T6 OOS Class 4	Medium-Heavy Duty Out-of-state Truck (GVWR 14001-16000 lbs)
	T6 OOS Class 5	Medium-Heavy Duty Out-of-state Truck (GVWR 16001-19500 lbs)
	T6 OOS Class 6	Medium-Heavy Duty Out-of-state Truck (GVWR 19501-26000 lbs)
	T6 OOS Class 7	Medium-Heavy Duty Out-of-state Truck (GVWR 26001-33000 lbs)
	T6TS	Medium-Heavy Duty Truck
**MT**	T6 Public Class 7	Medium-Heavy Duty Public Fleet Truck (GVWR 26001-33000 lbs)
	T6 Utility Class 7	Medium-Heavy Duty Utility Fleet Truck (GVWR 26001-33000 lbs)
	T6 Instate Tractor Class 7	Medium-Heavy Duty Tractor Truck (GVWR 26001-33000 lbs)
	T6 Instate Delivery Class 7	Medium-Heavy Duty Delivery Truck (GVWR 26001-33000 lbs)
	T6 Instate Other Class 7	Medium-Heavy Duty Other Truck (GVWR 26001-33000 lbs)
	T6 CAIRP Class 7	Medium-Heavy Duty CA International Registration Plan Truck (GVWR 2600133000 lbs)
	T6 OOS Class 7	Medium-Heavy Duty Out-of-state Truck (GVWR 26001-33000 lbs)
	T7 Single Concrete/Transit Mix Class 8	Heavy-Heavy Duty Single Unit Concrete/Transit Mix Truck (GVWR 33001 lbs and over)
	T7 Single Dump Class 8	Heavy-Heavy Duty Single Unit Dump Truck (GVWR 33001 lbs and over
	T7 Single Other Class 8	Heavy-Heavy Duty Single Unit Other Truck (GVWR 33001 lbs and over)
	T7 SWCV Class 8	Heavy-Heavy Duty Solid Waste Collection Truck (GVWR 33001 lbs and over)
**HT**	T6 Public Class 7	Medium-Heavy Duty Public Fleet Truck (GVWR 26001-33000 lbs)
	T6 Utility Class 7	Medium-Heavy Duty Utility Fleet Truck (GVWR 26001-33000 lbs)
	T6 Instate Tractor Class 7	Medium-Heavy Duty Tractor Truck (GVWR 26001-33000 lbs)
	T6 Instate Delivery Class 7	Medium-Heavy Duty Delivery Truck (GVWR 26001-33000 lbs)

T6 Instate Other Class 7	Medium-Heavy Duty Other Truck (GVWR 26001-33000 lbs)
T6 CAIRP Class 7	Medium-Heavy Duty CA International Registration Plan Truck (GVWR 2600133000 lbs)
T6 OOS Class 7	Medium-Heavy Duty Out-of-state Truck (GVWR 26001-33000 lbs)
T7 Single Concrete/Transit Mix Class 8	Heavy-Heavy Duty Single Unit Concrete/Transit Mix Truck (GVWR 33001 lbs and over)
T7 Single Dump Class 8	Heavy-Heavy Duty Single Unit Dump Truck (GVWR 33001 lbs and over)
T7 Single Other Class 8	Heavy-Heavy Duty Single Unit Other Truck (GVWR 33001 lbs and over)
T7 SWCV Class 8	Heavy-Heavy Duty Solid Waste Collection Truck (GVWR 33001 lbs and over)

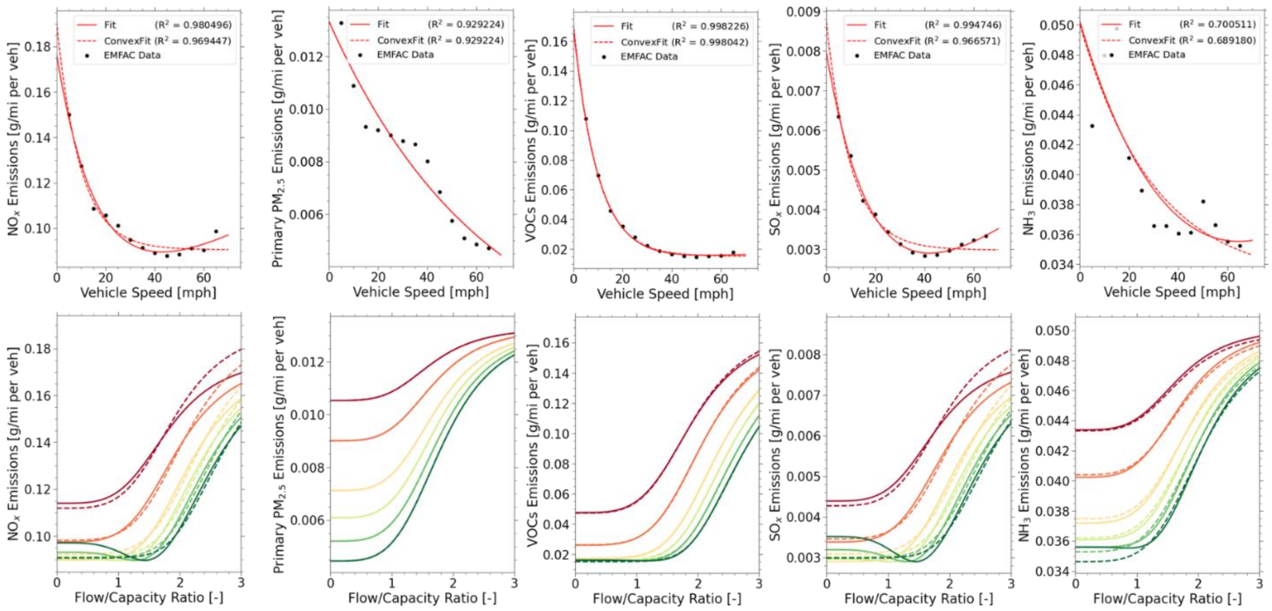

Figure S1: Primary $PM_{2.5}$, NO_x, VOC, SO_x, and NH_3 light-duty vehicle (LDV) emission rates calculated in [g/mi per vehicle] as a function of vehicle speed. Original emission data points are shown as black dots and are derived from the California Air Resources Board (ARB) EMission FACtor (EMFAC) average-speed, static-emissions model. Emission function curves and convex-adjusted curves are generated for each pollutant and are shown in solid red and dashed red, respectively. Emission rates are also plotted as a function of the optimization objective function in the form of flow-to-capacity ratio for six different free-flow speeds (FFS). Solid lines show the original emission curves while the dashed lines show the convex-adjusted emission curves.

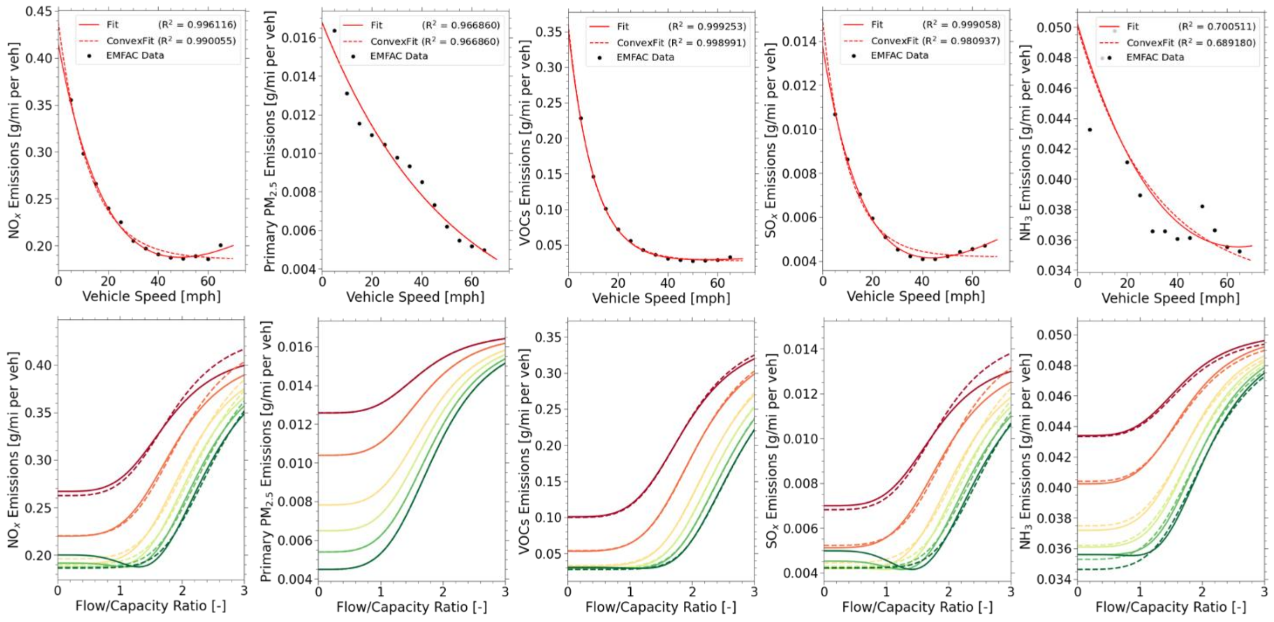

Figure S2: Primary $PM_{2.5}$, NO_x, VOC, SO_x, and NH_3 medium-duty vehicle (MDV) emission rates calculated in [g/mi per vehicle] as a function of vehicle speed. Original emission data points are shown as black dots and are derived from the California Air Resources Board (ARB) EMission FACtor (EMFAC) average-speed, static-emissions model. Emission function curves and convex-adjusted curves are generated for each pollutant and are shown in solid red and dashed red, respectively. Emission rates are also plotted as a function of the optimization objective function in the form of flow-to-capacity ratio for six different free-flow speeds (FFS). Solid lines show the original emission curves while the dashed lines show the convex-adjusted emission curves.

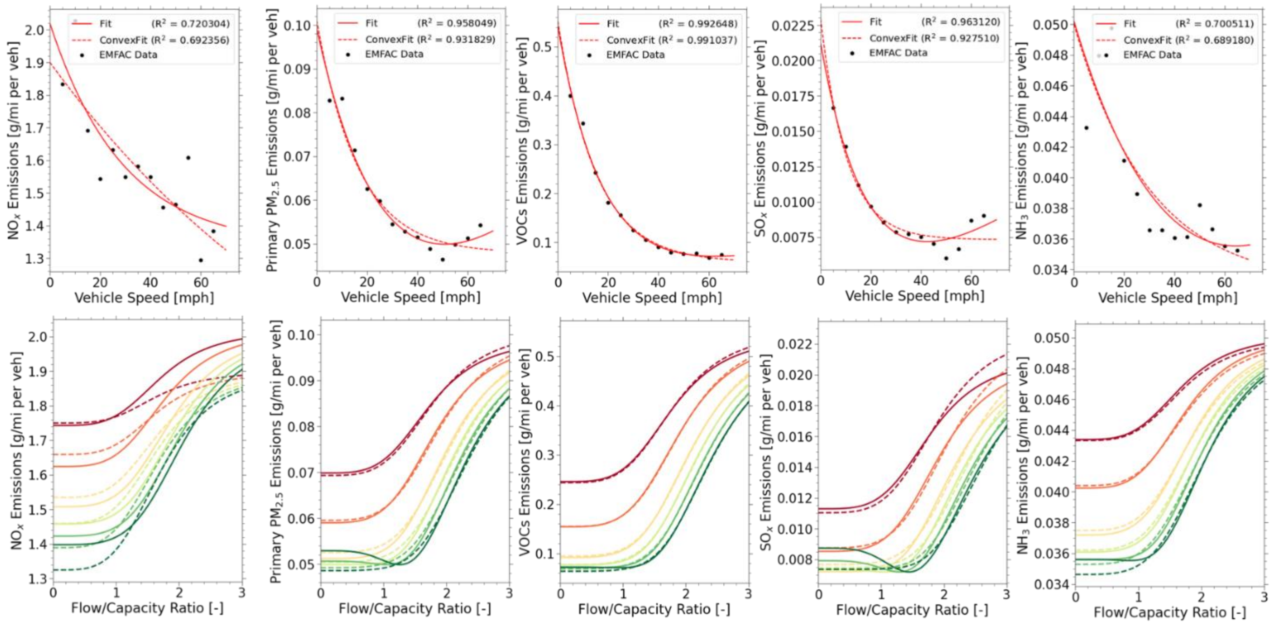

Figure S3: Primary $PM_{2.5}$, NO_x, VOC, SO_x, and NH_3 light-duty trucks (LT) emission rates calculated in [g/mi per vehicle] as a function of vehicle speed. Original emission data points are shown as black dots and are derived from the California Air Resources Board (ARB) EMission FACtor (EMFAC) average-speed, static-emissions model. Emission function curves and convex-adjusted curves are generated for each pollutant and are shown in solid red and dashed red, respectively. Emission rates are also plotted as a function of the optimization objective function in the form of flow-to-capacity ratio for six different free-flow speeds (FFS). Solid lines show the original emission curves while the dashed lines show the convex-adjusted emission curves.

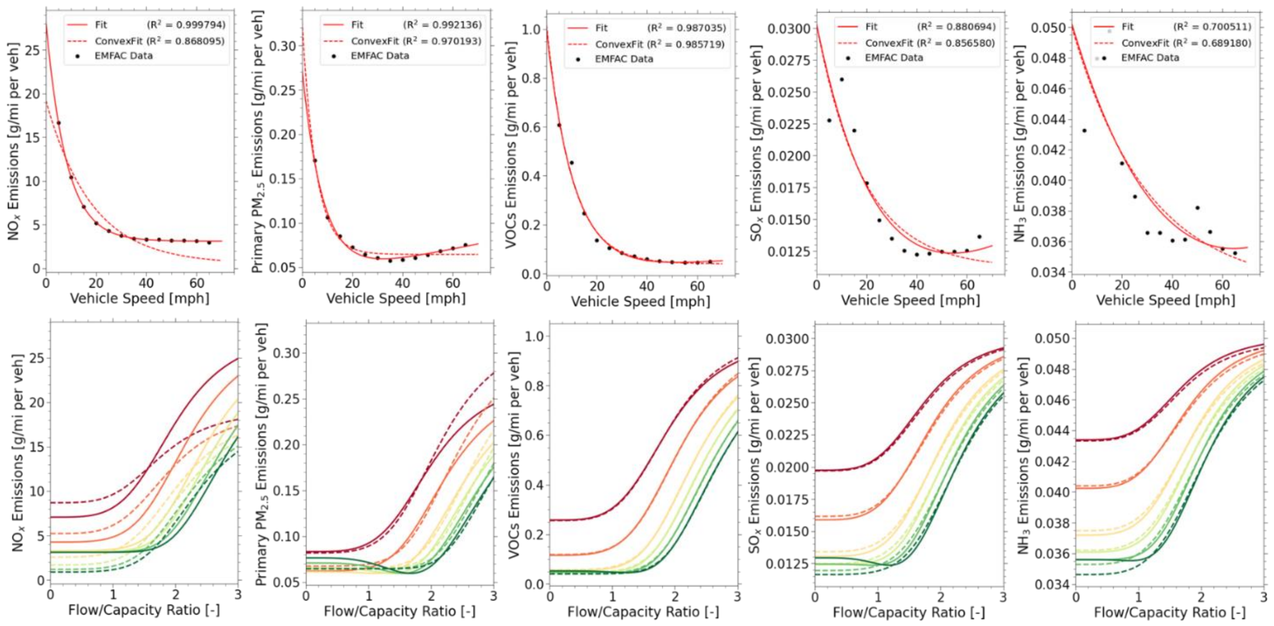

Figure S4: Primary $PM_{2.5}$, NO_x, VOC, SO_x, and NH_3 medium-duty trucks (MT) emission rates calculated in [g/mi per vehicle] as a function of vehicle speed. Original emission data points are shown as black dots and are derived from the California Air Resources Board (ARB) EMission FACtor (EMFAC) average-speed, static-emissions model. Emission function curves and convex-adjusted curves are generated for each pollutant and are shown in solid red and dashed red, respectively. Emission rates are also plotted as a function of the optimization objective function in the form of flow-to-capacity ratio for six different free-flow speeds (FFS). Solid lines show the original emission curves while the dashed lines show the convex-adjusted emission curves.

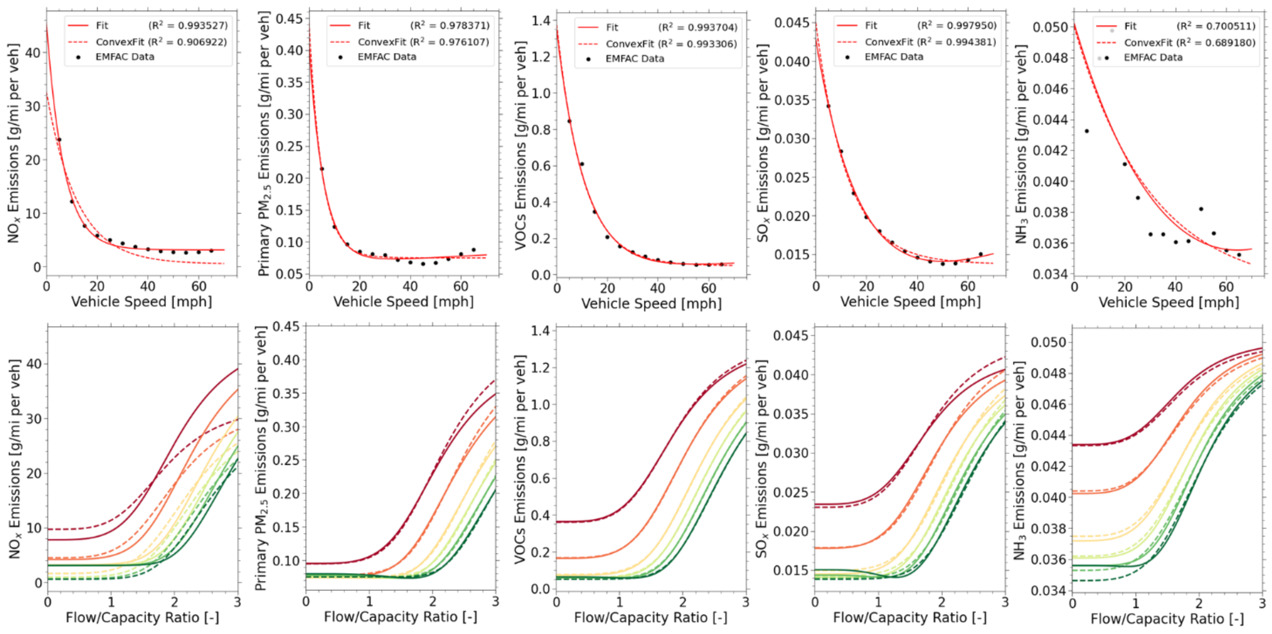

Figure S5: $PM_{2.5}$, NO_x, VOC, SO_x, and NH_3 heavy-duty trucks (HT) emission rates calculated in [g/mi per vehicle] as a function of vehicle speed. Original emission data points are shown as black dots and are derived from the California Air Resources Board (ARB) EMission FACtor (EMFAC) average-speed, static-emissions model. Emission function curves and convex-adjusted curves are generated for each pollutant and are shown in solid red and dashed red, respectively. Emission rates are also plotted as a function of the optimization objective function in the form of flow-to-capacity ratio for six different free-flow speeds (FFS). Solid lines show the original emission curves while the dashed lines show the convex-adjusted emission curves.

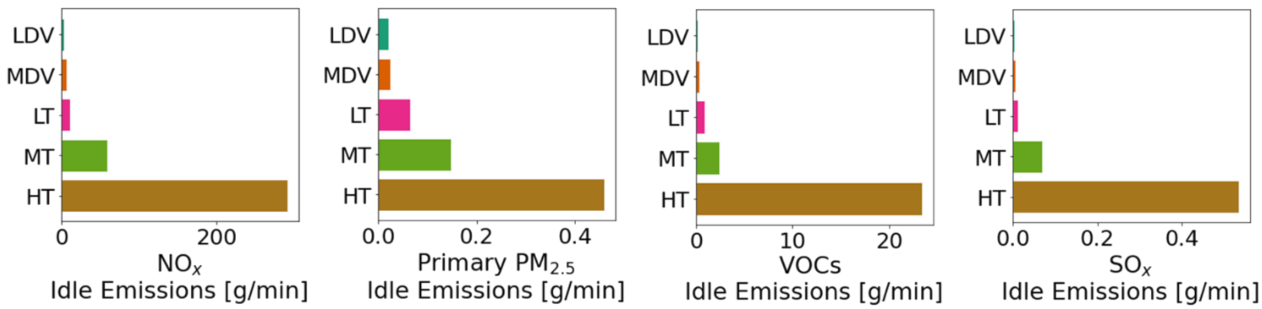

Figure S6: Primary $PM_{2.5}$, NO_x, VOC, and SO_x, idling emission factors [g per idling mins] for different vehicle classes

EMFAC provides data regarding the proportion of vehicle miles travelled that are completed using electric energy for both battery electric vehicles (BEVs) and plug-in hybrid electric vehicles (PHEVs), as well as electric energy consumed per miles travelled traveled disaggregated by vehicle speed [kWh/mi]. Electric vehicles miles traveled make up 3% of LDVs and less than 0.5% of truck-based trips in the baseline run. EMFAC also provides data regarding about how much energy is consumed per miles traveled disaggregated by vehicle speed [kWh/mi], which is shown in **Figure S7** for BEVs and PHEVs. The reduction in tailpipe emissions due to the presence of EVs within the fleet is factored into the base emission functions used in the TA model. However, electricity-based emissions are estimated once the assignment procedure is completed and the flow results are obtained. The total energy consumed by EVs within the network is calculated for all TOD periods. Total kWh consumed per hour of day is then calculated using charging habit data (i.e., typical charging session time and duration).[5] Next, energy consumption due to light-rail use for public transport is also quantified based on daily light-rail route. Data regarding light-rail energy consumption per vehicle miles traveled is sourced from Chester and Horvath.[6]

A scenario with the added energy requirements from EV and light-rail use is run in addition to a baseline run where those energy requirements are omitted. The difference in emissions and exposure between both scenarios is allocated to the EVs and light-rail use. Energy requirements for the EVs and light-rail are also grouped by TOD period to allow for comparison against the vehicle emissions. Energy requirements by TOD for both EV charging and light-rail use is shown in **Figure S8**. The majority of EV charging takes place overnight during TOD 1, slightly decreases during TOD 2 – 4, rises to a small peak in TOD 5, and then lowers to a constant baseline for the remainder of the day. Light-rail electricity demand has two small peaks during peak hours (TOD 3 and TOD 7). It is relatively constant throughout the rest of the except for the long overnight period (TOD 1) where demand is high due to the late night and early morning light-rail trips that take place.

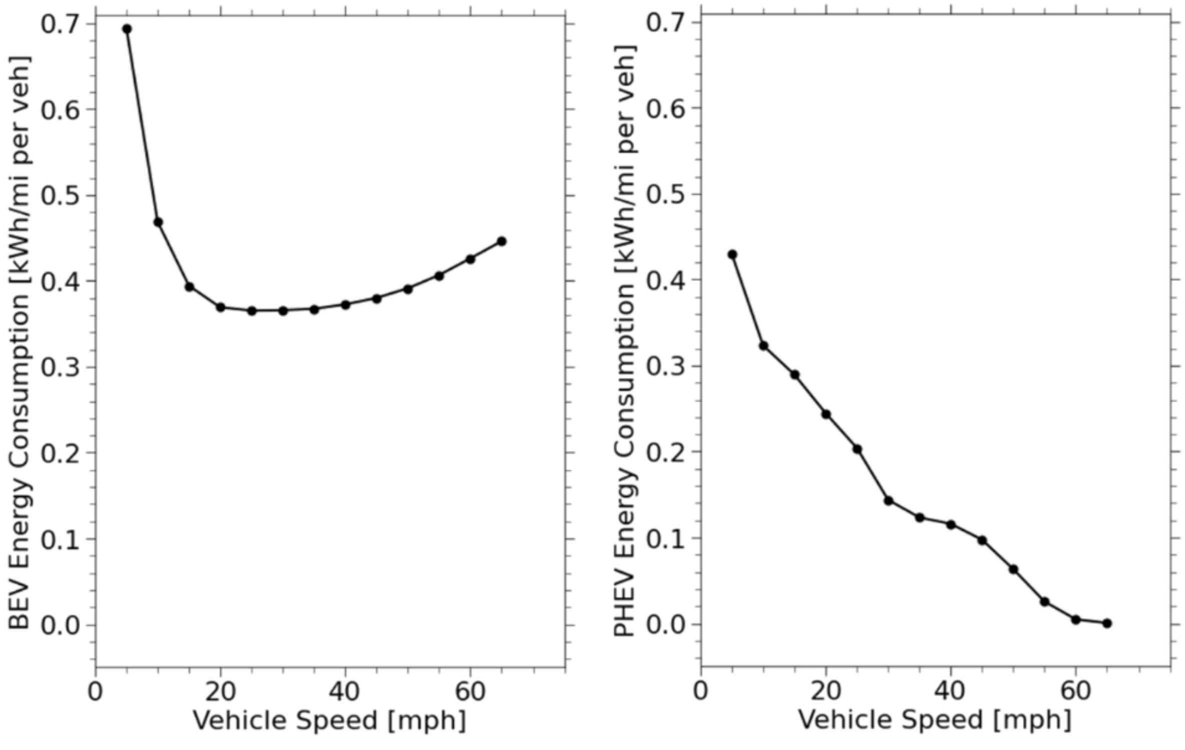

Figure S7: Electric energy consumption [kWh/mi] for battery electric vehicles (BEVs) and plug-in hybrid electric vehicles (PHEV) disaggregated by vehicle speed [mph].

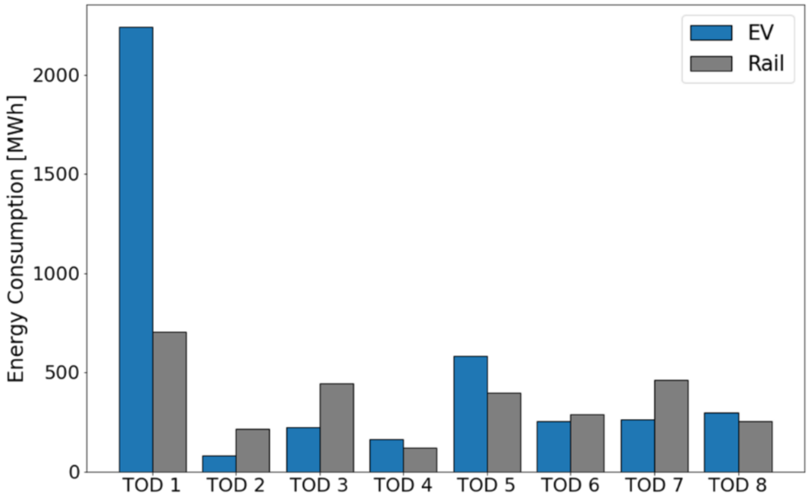

Figure S8: Electric energy demand [MWh] due to electric vehicle (EV) charging and light-rail use disaggregated by time-of-day (TOD) period. (TOD 1: The ten-hour late evening-early morning off-peak period (8:00 p.m. to 6:00 a.m.); TOD 2: The shoulder hour preceding the AM peak hour (6:00 to 7:00 a.m.); TOD 3: The AM peak two hours (7:00 to 9:00 a.m.); TOD 4: The shoulder hour following the AM peak period (9:00 to 10:00 a.m.); TOD 5: A four-hour midday period (10:00 a.m. to 2:00 p.m.); TOD 6: The two-hour shoulder period preceding the PM peak period (2:00 to 4:00 p.m.); TOD 7: The PM peak two hours (4:00 to 6:00 p.m.); TOD 8: The two-hour shoulder period following the PM peak period (6:00 to 8:00 p.m.)).

Exposure damages are quantified using concentration-response functions[7,8] in addition to the value of statistical life (VSL) metric[9]. Two different sets of concentration-response functions are used. The first follows the relationship developed in Nasari et al.[10] and Burnett et al.[11] and is shown in **(5)**. The second concentration-response function follows Krewski et al.[12] and Lepeule et al.[13] and is shown in **(6)**. Excess mortality rates in the different exposure zones are then calculated using **(7)** and transformed into monetary damages using **(8)**.

$$\mathrm{HR}(C_m) = \exp\left(\frac{\gamma \times \ln(C_m + 1)}{1 + \exp\left(\frac{-C_m - \delta}{\lambda}\right)}\right) \quad (\mathbf{5})$$

$$\mathrm{HR}(C_m) = \exp(\phi C_m) \quad (\mathbf{6})$$

$$\Delta\mathrm{Mortality_m} = \sum_{m \in M} (\mathrm{HR}(C_m) - 1) I_m Pop_m \quad (\mathbf{7})$$

$$\$\mathrm{Damages_m} = \Delta\mathrm{Mortality_m} \times \mathrm{VSL} \quad (\mathbf{8})$$

where:

$\mathrm{HR}(C_m)$: hazard ratio of mortality incidence at $PM_{2.5}$ concentration (C_m) in exposure zone m

$\gamma,\ \delta,\ \lambda,\ \phi$: empirically determined calibration constants [$\gamma = 0.0478, \delta = 6.94, \lambda = 3.37, \phi = \left(\frac{\ln(1.06)}{10}; \frac{\ln(1.078)}{10}; \frac{\ln(1.14)}{10}\right)$]

I_m: underlying incident rate in exposure zone m

Pop_m: population numbers in exposure zone m

VSL: U.S. EPA recommended value of statistical life adjusted to 2019 $ (~$10M/death)

1.4 $PM_{2.5}$ Exposure Reduction Strategies

1.4.1 Future Vehicle Fleet

Here we assume the accelerated adoption of future vehicle fleet mixes into the current CMA vehicle fleet mix. This strategy reduces overall vehicle fleet emission rates since future fleet mixes include a higher penetration of AFVs as well as high efficiency (i.e., lower emission) vehicles given that there will be a higher proportion of newer model year vehicles, with older model years being abandoned with time. Projections of the potential future fleet mixes were obtained from EMFAC.[14] Three different scenarios are modeled assuming fleet mixes for the years 2030, 2040, and 2050. It is assumed that they are adopted while holding all other present day baseline conditions, meaning that flow assignments for the UET will remain unchanged. EMFAC assumes that 9%, 11%, and 12% and 4%,6%, and 7% of future LDV and MDV vehicle-

miles traveled will be powered by electricity for the 2030, 2040, and 2050 fleets, respectively. Additionally, 8%, 36%, and 51% (LT) 8%; 32%, and 44% (MT); and 4%, 12%, and 14% (HT) of future truck vehicle-miles traveled will be powered by electricity for the 2030, 2040, and 2050 fleets, respectively. The additional electricity generation due to charging is also accounted for and shown in **Figure S9** for the three vehicle fleet years. The $PM_{2.5}$ exposure resulting from the additional electricity generation is quantified using the exposure-based OPF developed in Bin Thaneya and Horvath.[15] The OPF model is run assuming both current grid conditions with the present EGU network as well as a future grid scenario corresponding to each of the three vehicle fleet years. The future grid scenarios assume a higher adoption of renewable generation which is described in detail in Bin Thaneya and Horvath.[15]

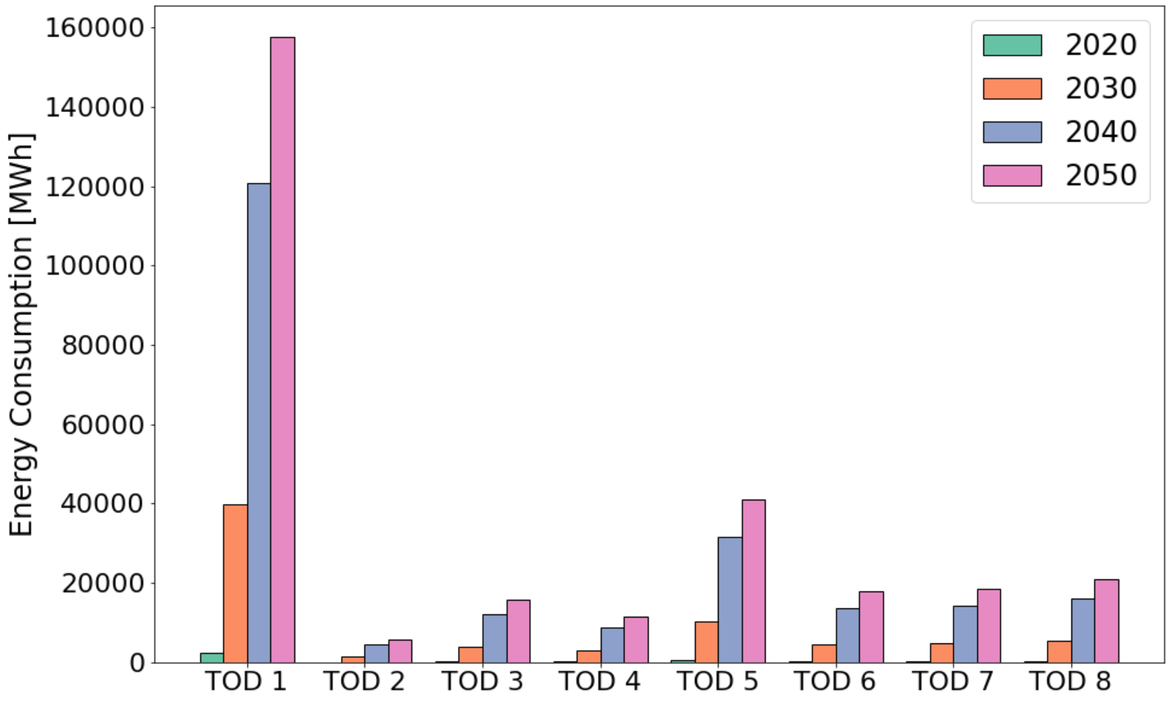

Figure S9: Projected electric energy demand [MWh] due to electric vehicle (EV) charging and light-rail use disaggregated by time-of-day (TOD) period assuming a 2030, 2040, and 2050 vehicle fleet. (TOD 1: The ten-hour late evening-early morning off-peak period (8:00 p.m. to

6:00 a.m.); TOD 2: The shoulder hour preceding the AM peak hour (6:00 to 7:00 a.m.); TOD 3: The AM peak two hours (7:00 to 9:00 a.m.); TOD 4: The shoulder hour following the AM peak period (9:00 to 10:00 a.m.); TOD 5: A four-hour midday period (10:00 a.m. to 2:00 p.m.); TOD 6: The two-hour shoulder period preceding the PM peak period (2:00 to 4:00 p.m.); TOD 7: The PM peak two hours (4:00 to 6:00 p.m.); TOD 8: The two-hour shoulder period following the PM peak period (6:00 to 8:00 p.m.)).

1.4.2 Personal Exposure Reduction Through Filtration

The second strategy assesses $PM_{2.5}$ personal exposure reduction achieved through the use of high-efficiency particulate arrestance (HEPA) filtration within households. Maestas et al.[16] showed that the use of commercially available filtration devices can reduce personal $PM_{2.5}$ exposure by 53% and 31% when using high-efficiency (HE: true-HEPA) and low-efficiency (LE: HEPA-type) filters, respectively. Based on baseline UET exposure results, census tracts that lie within the highest percentile of $PM_{2.5}$ concentrations and intake were identified. Due to how large the exposure domain is, the analysis was limited to census tracts that lie within the CMA since that is where most of the exposure damages are concentrated within. Two scenarios are run where it is assumed that each household within those census tracts would be provided with either an LE or HE filter in each case. Exposure reductions are applied to census tracts in the top 50th, 75th, and 90th percentile of damages (shown in **Figure S10**) to quantify how much reductions can be achieved when each level is targeted. The total capital costs required for purchasing the filtration devices and filters were also calculated for all the scenarios. We follow the assumption made in the Maestas et al.[16] in which two portable air filters are placed within each household (one in the bedroom and one in the main living space of each resident). We then use American Community Survey data[17] to estimate the number of residence units found within each of the higher exposure census tracts, and assume that two air filters are supplied to each of those housing units. We then calculate the total capital costs required to purchase the air filters[18,19] and the commercially-available personal air filtration units[20] for each scenario..

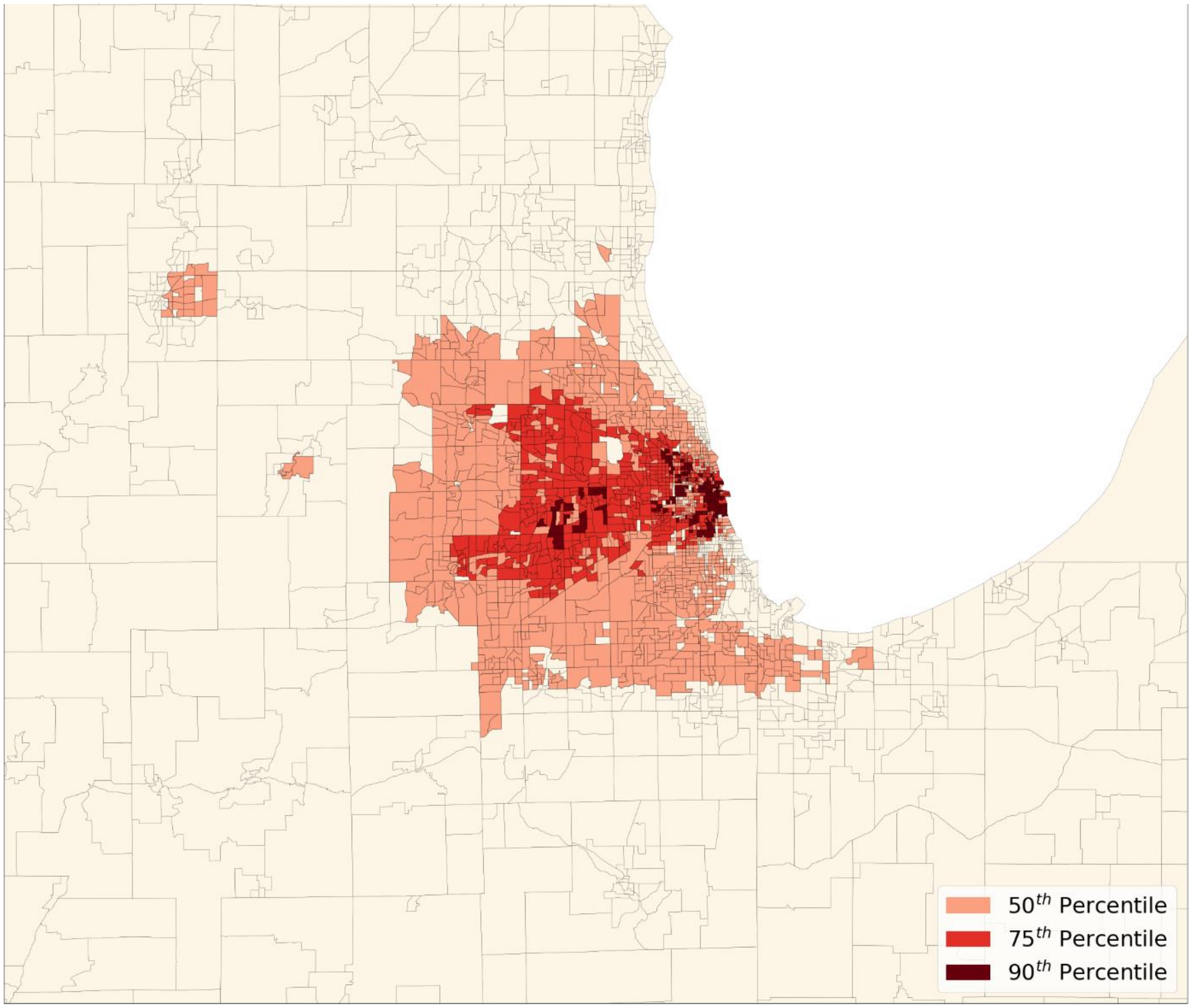

Figure S10: Census tracts showing census tracts that lie within the 90th, 75th, and 50th percentile of damages within baseline conditions.

1.4.3 Higher Public Transportation Use

The third strategy involves changes in the baseline mode split share, where an increase in public transportation for daily trips is assumed using both bus and light-rail transport. A 5%, 10%, 20%, and 40% increase in public transportation use is analyzed, replacing individual LDV vehicle trips for all cases. Each additional bus or rail trip that is added to the network is assumed to replace a certain equivalent amount of LDV trips based on bus and rail ridership reports from the public transport authorities.[21–23] **Figure S11** shows the different bus and light-rail routes that are located

within the network as well as the O-D centroids where passenger trips begin and terminate. First, the increase in the amount of bus and light-rail trips is calculated, followed by the number of trips that the increase would replace. It is assumed that any given LDV trip whose O-D centroids lie within a 0.5-mile radius from a bus or light-rail station is eligible to be replaced with a bus or transit trip. This effectively reduces the number of LDV in the O-D matrices that are input in the TA. The TA is then run with the modified O-D matrices to quantify the reduction in congestion and travel-time within the network. The reduction in LDV emissions as well as increase in bus emissions and EGU emissions due to the increase in electricity generation from the additional rail trips are also quantified.

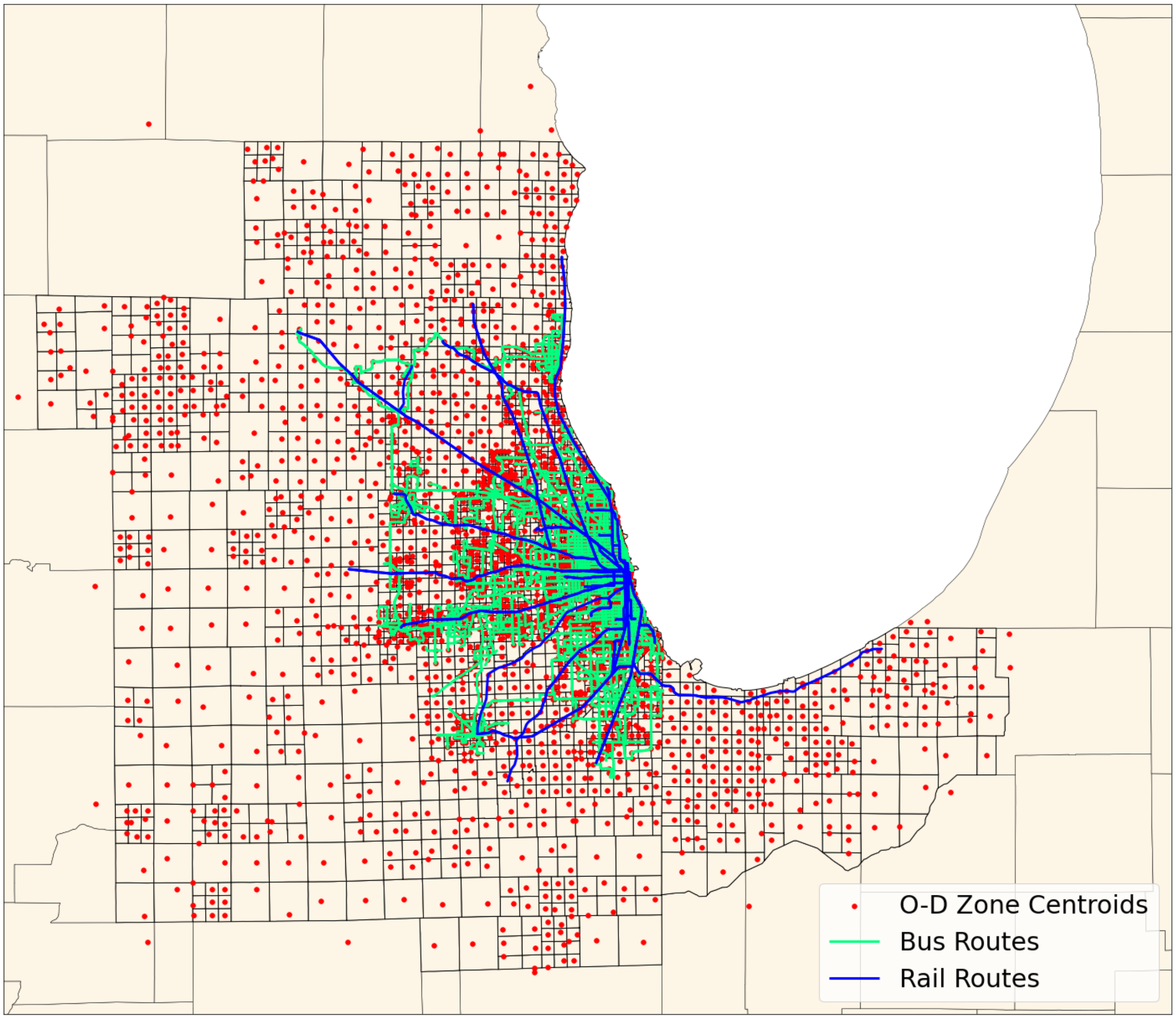

Figure S11: Map showing bus and light-rail public transit routes as well as O-D zone centroids within the transportation network.

1.4.4 Exposure-Based Truck Routing

The final two strategies involve applying a modified version of the SOI. The base SOI optimization assumes that all vehicle trips can be controlled and routes vehicles in a manner that minimizes $PM_{2.5}$ exposure. Applying vehicle routing at such a level may not be entirely feasible for personal vehicles, especially since many would choose the route with the minimum travel-time. However, targeting truck-based trips with SOI based routing control is more achievable, especially through policy mechanisms aimed at reducing the overall pollution impacts of trucks.

UET exposure results showed that trucks were responsible for about half of all exposure damages, thus mitigating their damages contributions could be effective in reducing overall impacts. Furthermore, it is found that the highest SOI reductions are achieved when congestion conditions are low, allowing more opportunity to reroute traffic to low *iF* links without any overload that can lead to high emissions. The lowest congestion conditions occurred during TOD 1 (the ten-hour late evening-early morning off-peak period (8:00 p.m. to 6:00 a.m.)). Thus, this strategy assumes that truck-based trips are moved from other TODs to TOD 1, such that 75% of all truck-based trips take place during this timeframe where they follow an SOI based routing principle.

1.4.5 Bi-objective Optimization of Travel Time and $PM_{2.5}$ Exposure

Baseline results showed that a large trade-off exists between the UET and SOI. The SOI achieves reduction in $PM_{2.5}$ exposure by rerouting vehicle flow to low *iF* links in the less populated CMA outskirts, but in doing so, it incurs a large amount of added travel time for network users since there is no consideration for travel time within the formulation. However, the UET and SOI objective functions can be combined to generate a set of trade-off optimal solutions known as pareto-optimal solutions. This new formulation can be solved using a weighted-sum method, where both objective functions are merged into a single formulation by multiplying each by a weight, forming a convex combination of objectives.[24–27] Weights are parametrically varied to obtain a pareto front. Since a large magnitude difference exists between the travel costs and exposure damages, each objective is normalized by the true intervals of their variation over the Pareto optimal set. To derive the bi-objective formulation, the UET and SOI objective functions from Bin Thaneya et al.[2] are defined in **(9.1)** and **(9.2)** as follows:

$$T(\mathbf{x}) = \min_{\mathbf{x},\mathbf{h}} \sum_{(i,j)\in A} \int_0^{x_{ij}} t_{ij}(\boldsymbol{x})d\boldsymbol{x} \qquad \mathbf{(9.1)}$$

$$C(\mathbf{x}) = \min_{\mathbf{x},\mathbf{h}} \sum_{p\in P} \sum_{m\in M} \sum_{(i,j)\in A} c_{pm-ij}(\boldsymbol{x_{ij}}) \qquad \mathbf{(9.2)}$$

The bi-objective optimization model is defined in **(10)** as follows:

$$\min_{\mathbf{x},\mathbf{h}} \; w\bar{T}(\mathbf{x}) + (1-w)\bar{C}(\mathbf{x}) \qquad \mathbf{(10)}$$

where $w \in [0,1]$ represents the weighting factor, and $\bar{T}(\mathbf{x})$ and $\bar{C}(\mathbf{x})$ represent the two normalized UET and SOI objective functions, respectively. The normalized objective functions are defined in **(11.1)** and **(11.2)** as follows:

$$\bar{T}(\mathbf{x}) = \frac{T(\mathbf{x}) - T^U(\mathbf{x}^{*\mathbf{UET}})}{T^N(\mathbf{x}^{*\mathbf{SOI}}) - T^U(\mathbf{x}^{*\mathbf{UET}})} \qquad \mathbf{(11.1)}$$

$$\bar{C}(\mathbf{x}) = \frac{C(\mathbf{x}) - C^U(\mathbf{x}^{*\mathbf{SOI}})}{C^N(\mathbf{x}^{*\mathbf{UET}}) - C^U(\mathbf{x}^{*\mathbf{SOI}})} \qquad \mathbf{(11.2)}$$

where $T^U(\mathbf{x}^{*\mathbf{UET}})$ and $C^U(\mathbf{x}^{*\mathbf{SOI}})$ represent the utopia points for the UET and SOI normalized objective functions, respectively, which yield the lower bounds of the pareto Optimal set. $T^N(\mathbf{x}^{*\mathbf{SOI}})$ and $C^N(\mathbf{x}^{*\mathbf{UET}})$ represent the nadir points of the UET and SOI normalized objective functions, respectively, which provide the upper bounds for the Pareto optimal set. $\mathbf{x}^{*\mathbf{UET}}$ and $\mathbf{x}^{*\mathbf{SOI}}$ represent the optimal flows for the UET and SOI when individually run, respectively. Each objective function is bounded by 0 and 1 post-normalization, which provides an equal magnitude to each objective. Using a bi-objective approach can help reduce flow on the most damaging links without incurring excessively large travel times. An additional issue with the SOI is identifying mechanism that can shift vehicles to shift from a UET based assignment to an SOI based one. Historically, toll-setting on high congestion roads has been formulated as a behavioral modeling mechanism to reduce flow on certain links during peak hours. A similar first-best

pricing scheme can be implemented where road tolls can be set that reflect the external exposure cost generated by each traveler.[28] The exposure tolls set on each roadway aim to internalize the exposure externality of travel, which allows travelers to consider minimizing their induced exposure costs in addition to their travel time costs when choosing their O-D route. Tolls for links are derived by combining the link *iF*s with the vehicle emission rates and the linearized exposure damages factor ($\boldsymbol{\nu}$) [$ per kgPM$_{2.5}$ inhaled], which is derived in Bin Thaneya and Horvath.[15] Exposure based tolls penalize travel on high exposure inducing links while still balancing travel time.

2. Supplementary Results

2.1 UET and SOI Network Flow, Travel Time, and Delay Results

Vehicle delay is a metric that can be used to measure network congestion. It is quantified by taking the difference between actual travel time and free-flow travel time on network links. **Figure S12(a)** plots vehicle delay hours for the UET and SOI assignments. Overall, the SOI leads to higher overall delay for all TODs. The level of delay intensifies during peak hours (i.e., TOD 3 and TOD 7) where the magnitude of delay in the SOI assignment can be 2x – 3x that of the UET. In terms of systemwide delay, the UET leads to a total of 1.6 million vehicle delay hours per day, while the SOI assignment leads to 6.4 million vehicle delay hours per day (+300%). An alternate measure of network congestion is the flow-to-capacity ratio of network links. The distribution of flow-to-capacity ratios is plotted in box plots in **Figure S12(b)** in the SI. The SOI generally shows a higher distribution of flow-to-capacity ratios for all TODs, where all percentiles as well as mean ratios are higher than that of the UET. The highest flow-to-capacity ratios are again observed during peak hours (TOD 3 and TOD 7), signifying high

congestion during those periods, as expected. The lowest congestion is observed during the overnight period (TOD 1).

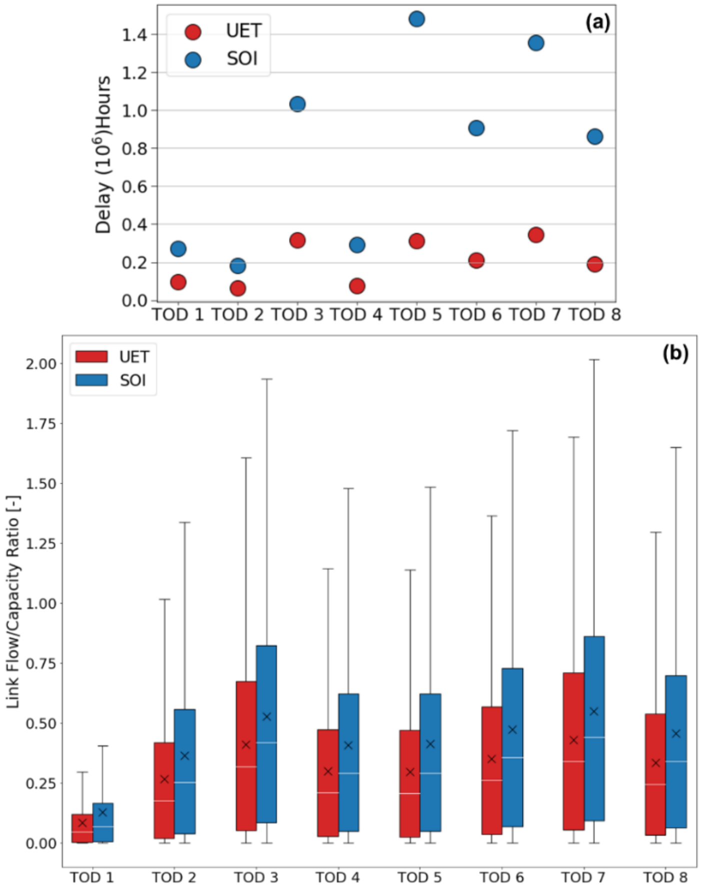

Figure S12: (a) User-equilibrium for time (UET) and system optimal for intake (SOI) delay hours disaggregated by time-of-day (TOD) period. (b) UET and SOI box-plot link flow-to-capacity ratio distributions for the UET and SOI disaggregated by TOD period. Black crosses and white lines show mean and median flow-to-capacity ratios, respectively. (TOD 1: The ten-hour late evening-early morning off-peak period (8:00 p.m. to 6:00 a.m.); TOD 2: The shoulder hour preceding the AM peak hour (6:00 to 7:00 a.m.); TOD 3: The AM peak two hours (7:00 to 9:00 a.m.); TOD 4: The shoulder hour following the AM peak period (9:00 to 10:00 a.m.); TOD 5: A four-hour midday period (10:00 a.m. to 2:00 p.m.); TOD 6: The two-hour shoulder period

preceding the PM peak period (2:00 to 4:00 p.m.); TOD 7: The PM peak two hours (4:00 to 6:00 p.m.); TOD 8: The two-hour shoulder period following the PM peak period (6:00 to 8:00 p.m.)). The increase in network congestion and delay caused by the SOI assignment is largely due to differences in network flow between both assignments. The SOI assignment overloads links with low *iF* in an attempt to reduce overall $PM_{2.5}$ exposure, without considering the increase of delay on those links. This is also due to the SOI relying heavily on using arterial roadways with low *iF*s as opposed to freeways located in higher population density tracts. The general differences in vehicle flow between both assignments show that the SOI moves flow away from the high *iF* links located in the higher population density Chicago urban center and onto the low *iF* links on the CMA outskirts. The rerouting trends do not vary much between off-peak and peak hours as shown in **Figures S13 – S20**, which plot links with increased vehicle flow in each of the UET (**subplot (a)**) and SOI (**subplot (b)**) relative to the other assignment for all TODs. They show that the SOI assignment reroutes a large amount of flow away from freeways /expressways in high population density census tracts. Only 9% of all travel time in the SOI assignment occurs on freeways and expressways, while 64% of all travel time occurs on local arterial roadways. High utilization of local roadways also leads to a large increase in idle time in the SOI assignment, which is 65% higher than idle time in UET assignment. Another factor that leads to the large increase in travel time in the SOI assignment is the major rerouting that occurs away from the inter-city roadways and onto the CMA outskirt links as shown in **Figures S13 – S20 (rerouting subplots)**.

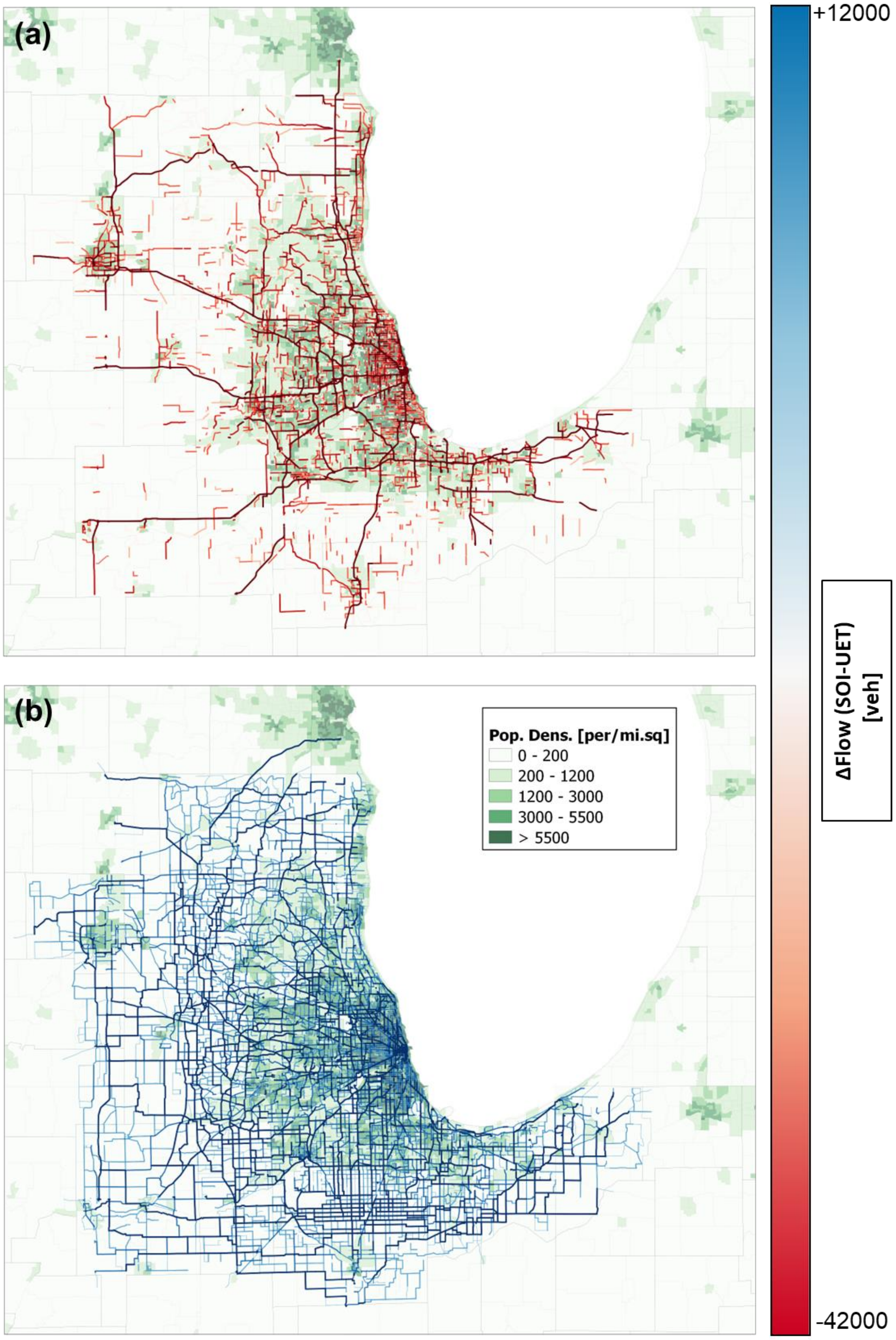

Figure S13: (a) Network map for off-peak TOD 1 showing links that have increased vehicle flow in the in the user equilibrium for time (UET) assignment relative to the system optimal for intake (SOI) assignment, whereas the opposite is shown in (b).

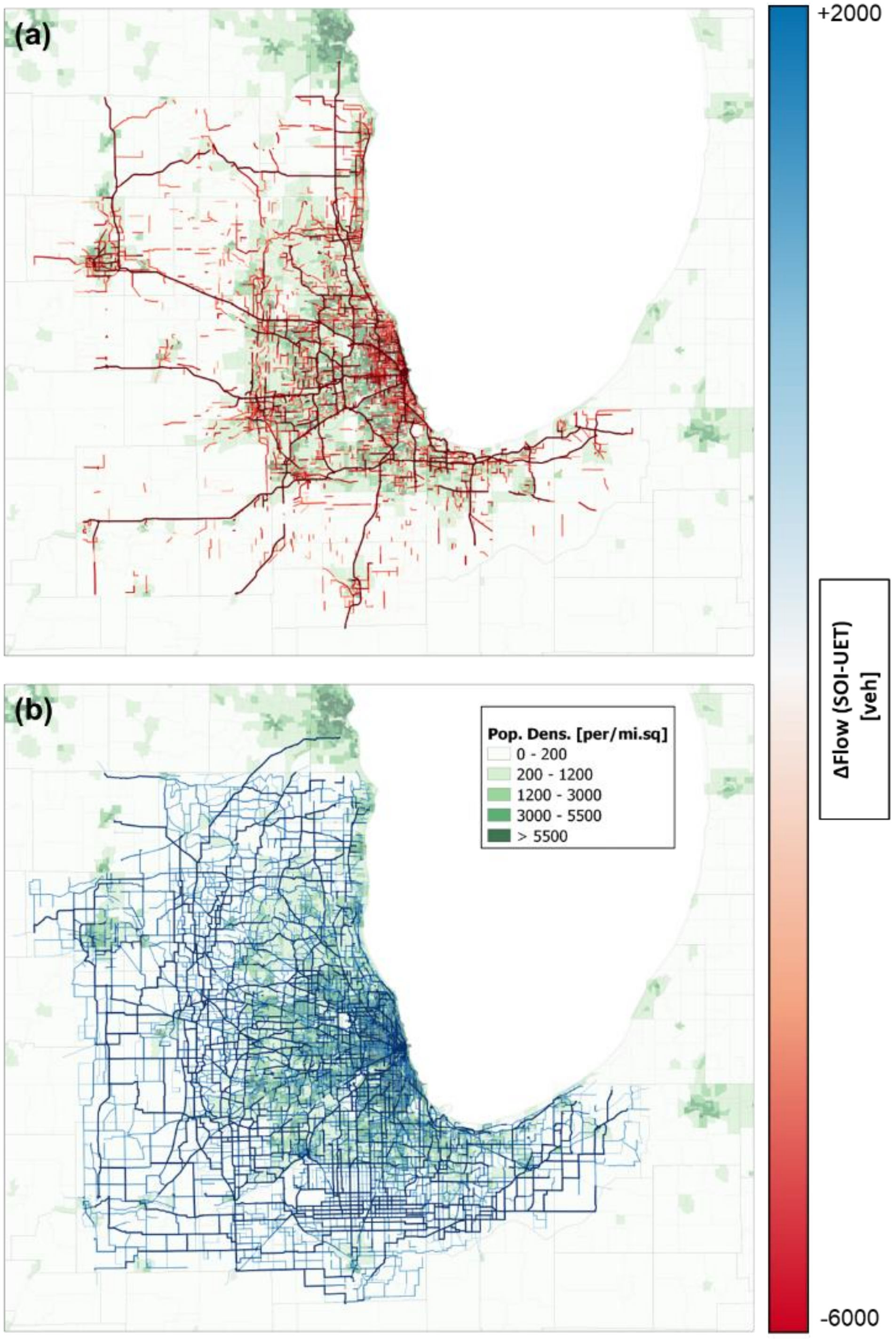

Figure S14: (a) Network map for peak TOD 2 showing links that have increased vehicle flow in the in the user equilibrium for time (UET) assignment relative to the system optimal for intake (SOI) assignment, whereas the opposite is shown in (b).

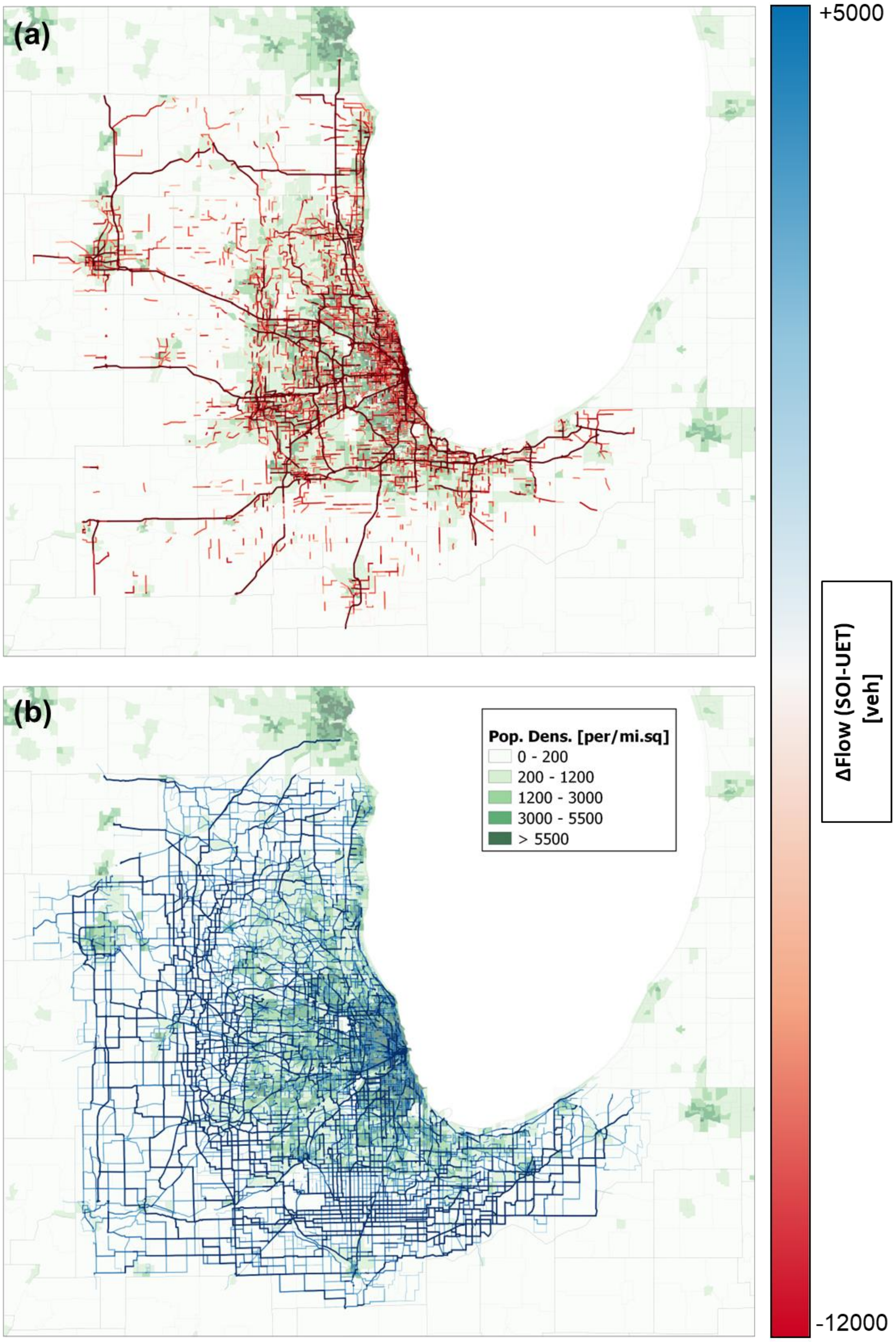

Figure S15: (a) Network map for peak TOD 3 showing links that have increased vehicle flow in the in the user equilibrium for time (UET) assignment relative to the system optimal for intake (SOI) assignment, whereas the opposite is shown in (b).

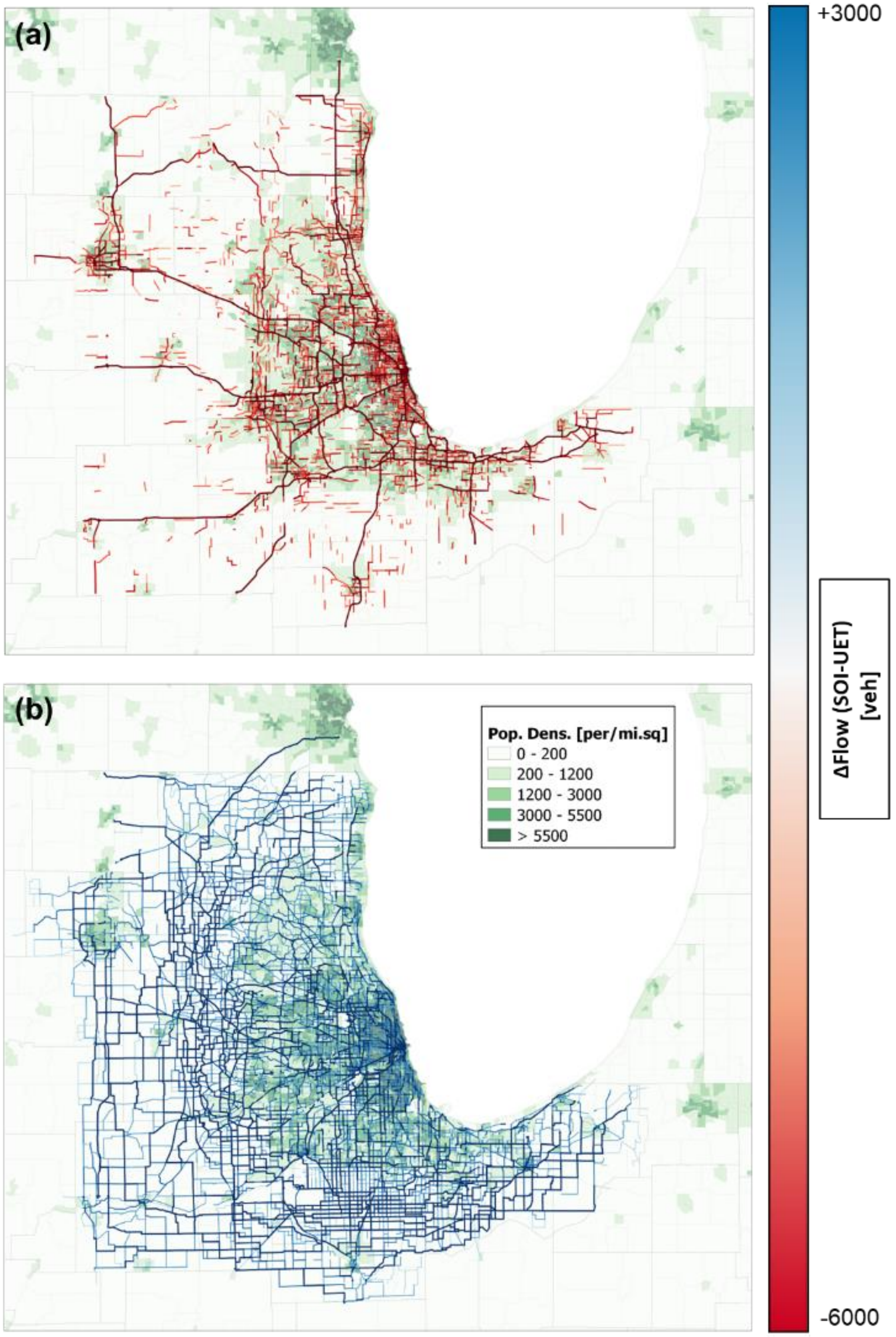

Figure S16: (a) Network map for peak TOD 4 showing links that have increased vehicle flow in the in the user equilibrium for time (UET) assignment relative to the system optimal for intake (SOI) assignment, whereas the opposite is shown in (b).

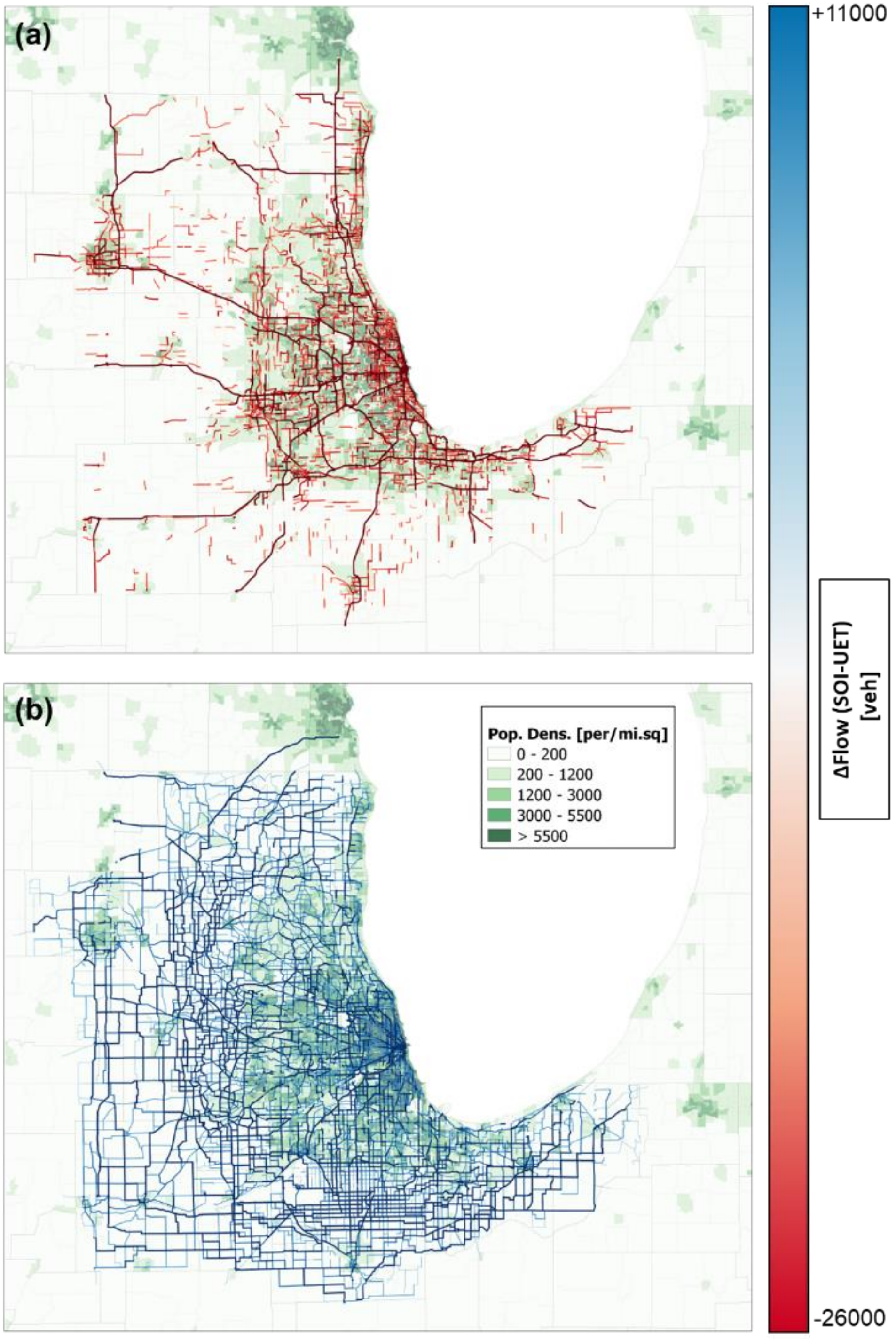

Figure S17: (a) Network map for peak TOD 5 showing links that have increased vehicle flow in the in the user equilibrium for time (UET) assignment relative to the system optimal for intake (SOI) assignment, whereas the opposite is shown in (b).

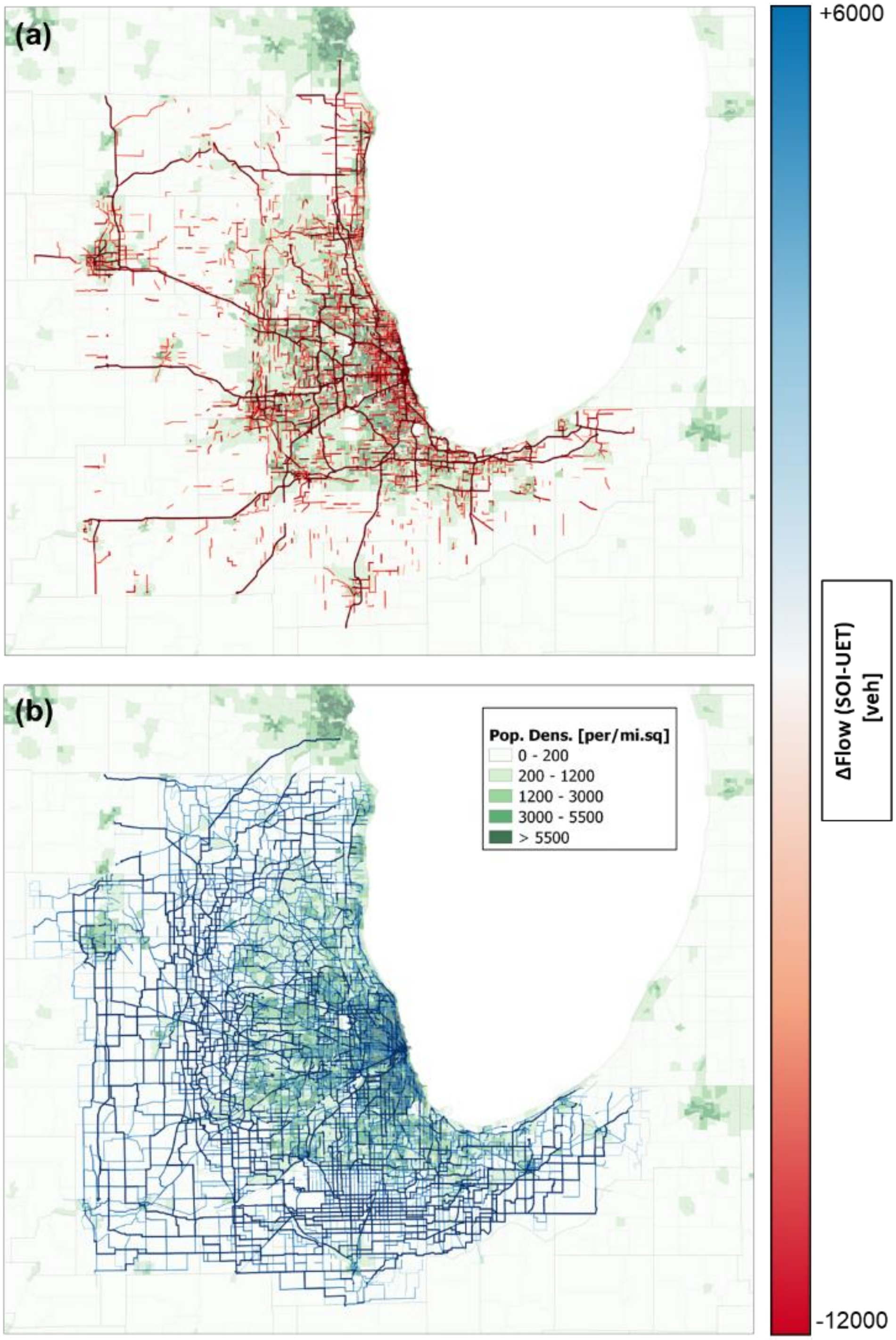

Figure S18: (a) Network map for peak TOD 6 showing links that have increased vehicle flow in the in the user equilibrium for time (UET) assignment relative to the system optimal for intake (SOI) assignment, whereas the opposite is shown in (b).

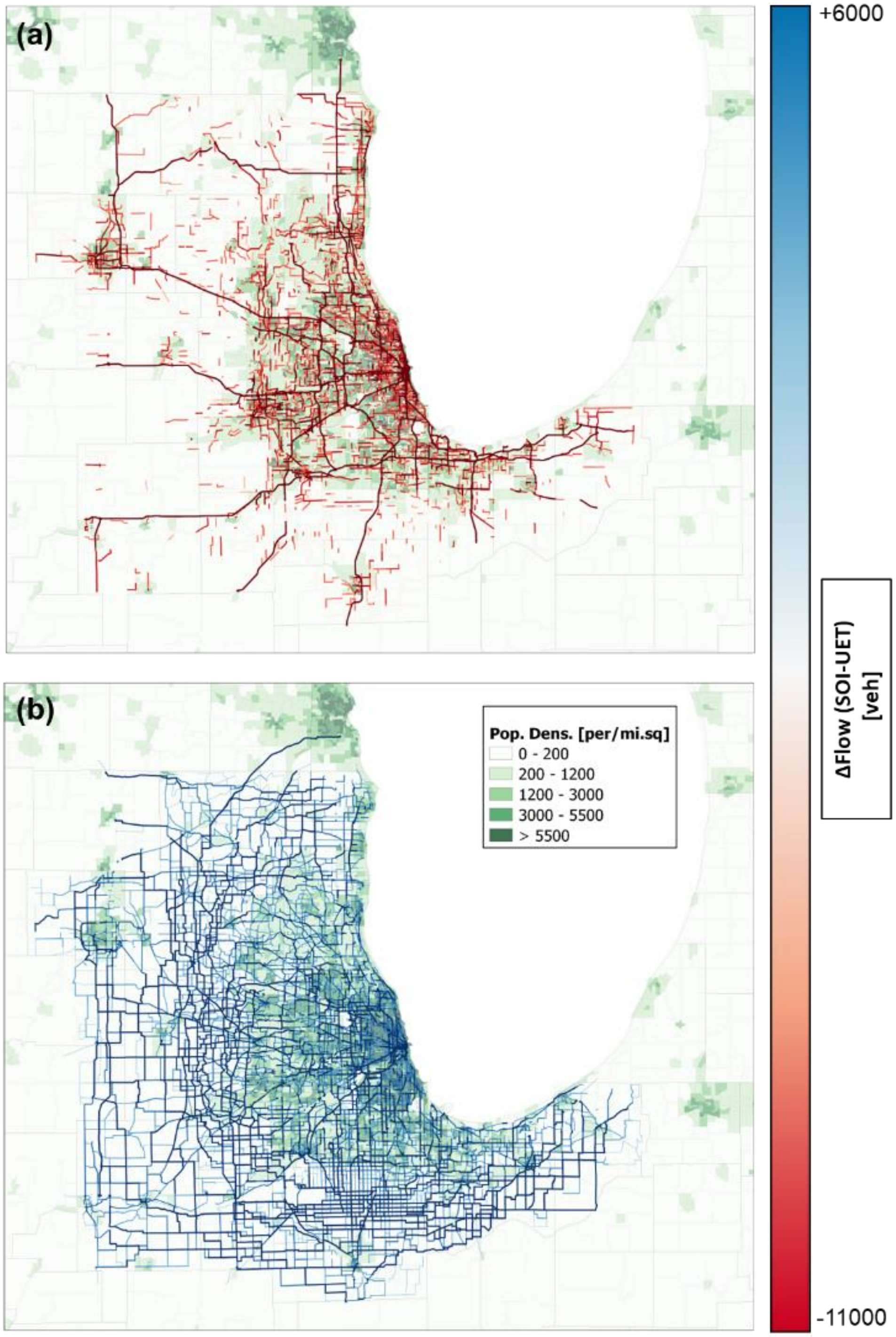

Figure S19: (a) Network map for peak TOD 7 showing links that have increased vehicle flow in the in the user equilibrium for time (UET) assignment relative to the system optimal for intake (SOI) assignment, whereas the opposite is shown in (b).

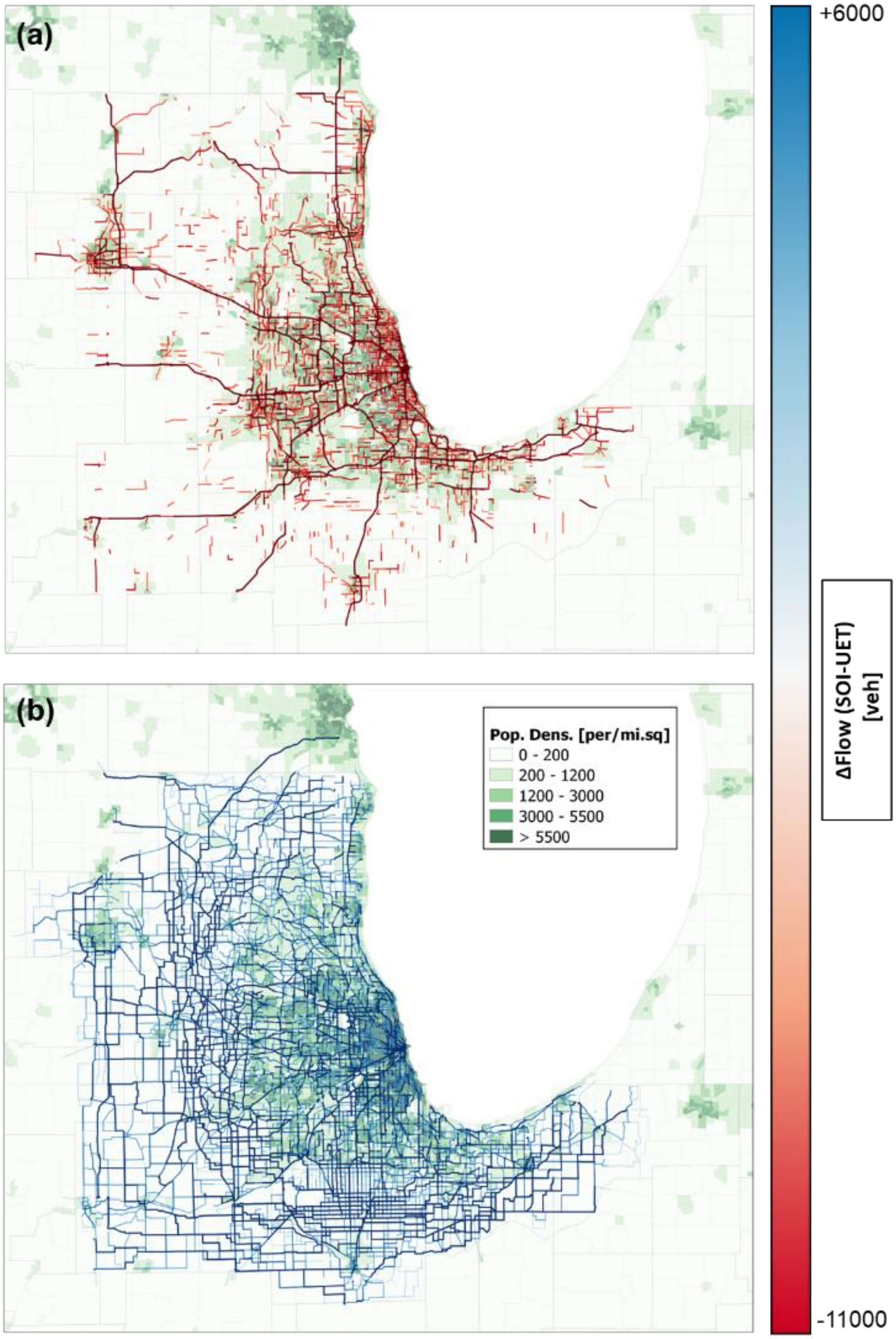

Figure S20: (a) Network map for peak TOD 8 showing links that have increased vehicle flow in the in the user equilibrium for time (UET) assignment relative to the system optimal for intake (SOI) assignment, whereas the opposite is shown in (b).

2.2 UET and SOI Emission and Exposure Results

Figure S21 plots vehicle-based emissions for the UET and SOI disaggregated by type of roadways the emissions take place on. The relative difference in emissions is also shown in **Figure S21**. The overall increase in emissions ranges between 2% − 15% for NO_x, primary $PM_{2.5}$, SO_x, and NH_3. The increase in emissions is larger for VOCs (20% − 40%). This is because VOCs are emitted at a higher magnitude during idling, and the SOI assignment utilizes roadways that end with intersections at a higher proportion relative to the UET, meaning that VOCs emissions due to idling will be larger. The distribution of emissions occurring on local roadways or freeways/expressways is similar to what was observed for travel time, where the majority of SOI emissions take place on signalized arterial roadways, whereas the UET has a relatively even split between signalized roadways and freeways/expressways.

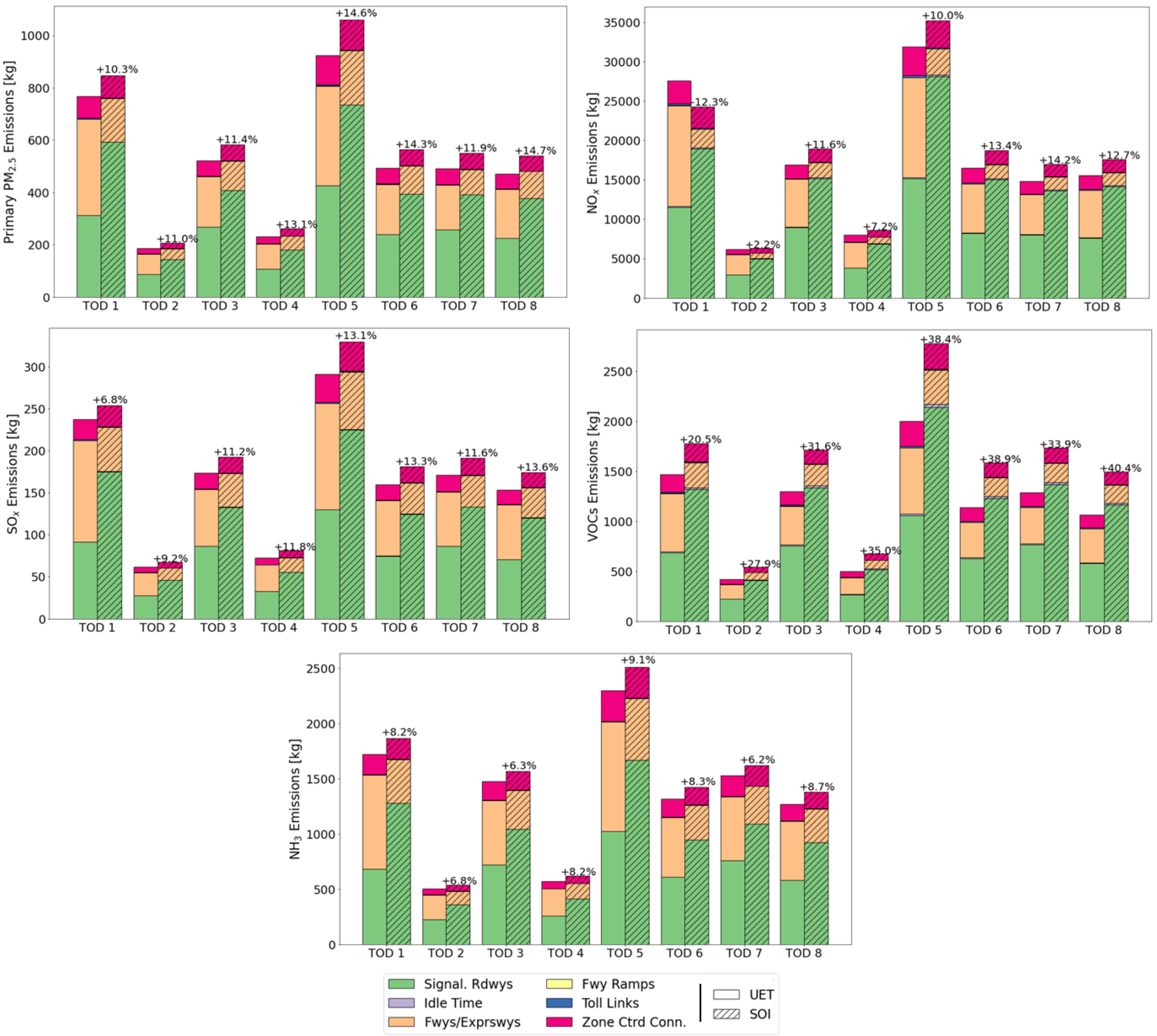

Figure S21: User-equilibrium for time (UET) and system optimal for intake (SOI) passenger emissions [kg] for primary $PM_{2.5}$, NO_x, SO_x, VOCs, and NH_3 disaggregated by time-of-day (TOD) period and roadways utilized. (LDV: Light-duty vehicles; MDV: Medium-duty vehicles; LT: Light-duty trucks; MT: Medium-duty trucks; HT: Heavy-duty trucks). (TOD 1: The ten-hour late evening-early morning off-peak period (8:00 p.m. to 6:00 a.m.); TOD 2: The shoulder hour preceding the AM peak hour (6:00 to 7:00 a.m.); TOD 3: The AM peak two hours (7:00 to 9:00 a.m.); TOD 4: The shoulder hour following the AM peak period (9:00 to 10:00 a.m.); TOD 5: A four-hour midday period (10:00 a.m. to 2:00 p.m.); TOD 6: The two-hour shoulder period

preceding the PM peak period (2:00 to 4:00 p.m.); TOD 7: The PM peak two hours (4:00 to 6:00 p.m.); TOD 8: The two-hour shoulder period following the PM peak period (6:00 to 8:00 p.m.)).

Figure S22 plots vehicle-based emissions for the UET and SOI disaggregated by vehicle type. EGU-based emissions due to EV charging and light-rail use are also included. Even with the inclusion of EGU based emissions, the SOI assignment leads to higher emissions relative to the UET. LDVs and MDVs emit 35% and 5% of primary $PM_{2.5}$ mass in both the UET and SOI assignments, respectively. Trucks are attributed with 43% of primary $PM_{2.5}$ emissions, while buses lead to less than 1% of emissions. EGUs lead to a noticeable increase in primary $PM_{2.5}$ emissions, especially in TOD 1 where most of EV charging takes place. Overall, EGUs account for about 15% of primary $PM_{2.5}$ emissions in both assignments. EGUs represent a smaller portion of NO_x emissions, where they account for about 2%. This is due to the large magnitude of NO_x emissions resulting from trucks, which emit the majority of NO_x emissions in both assignments (70%). This is followed by LDVs, which emit about 20% of all NO_x emissions, with the remainder split between MDVs (4%) and buses (1%). About 50% of all VOCs emissions are due to LDVs, with 40% attributable MDVs and trucks. The remaining small proportion of emissions are due to public transport and EV charging. The largest increase in emissions from the inclusion of EGUs comes from SO_x emissions. This is due to the Illinois electricity mix still having some reliance on coal-based generation. Approximately 66% of all SO_x emissions are due to EGUs, with the remaining 33% coming from vehicle emissions. The largest source of NH_3 - emissions are LDVs, with the remaining 20% being split evenly between MDVs and trucks. Buses and EGUs do not make up a large proportion of NH_3 emissions.

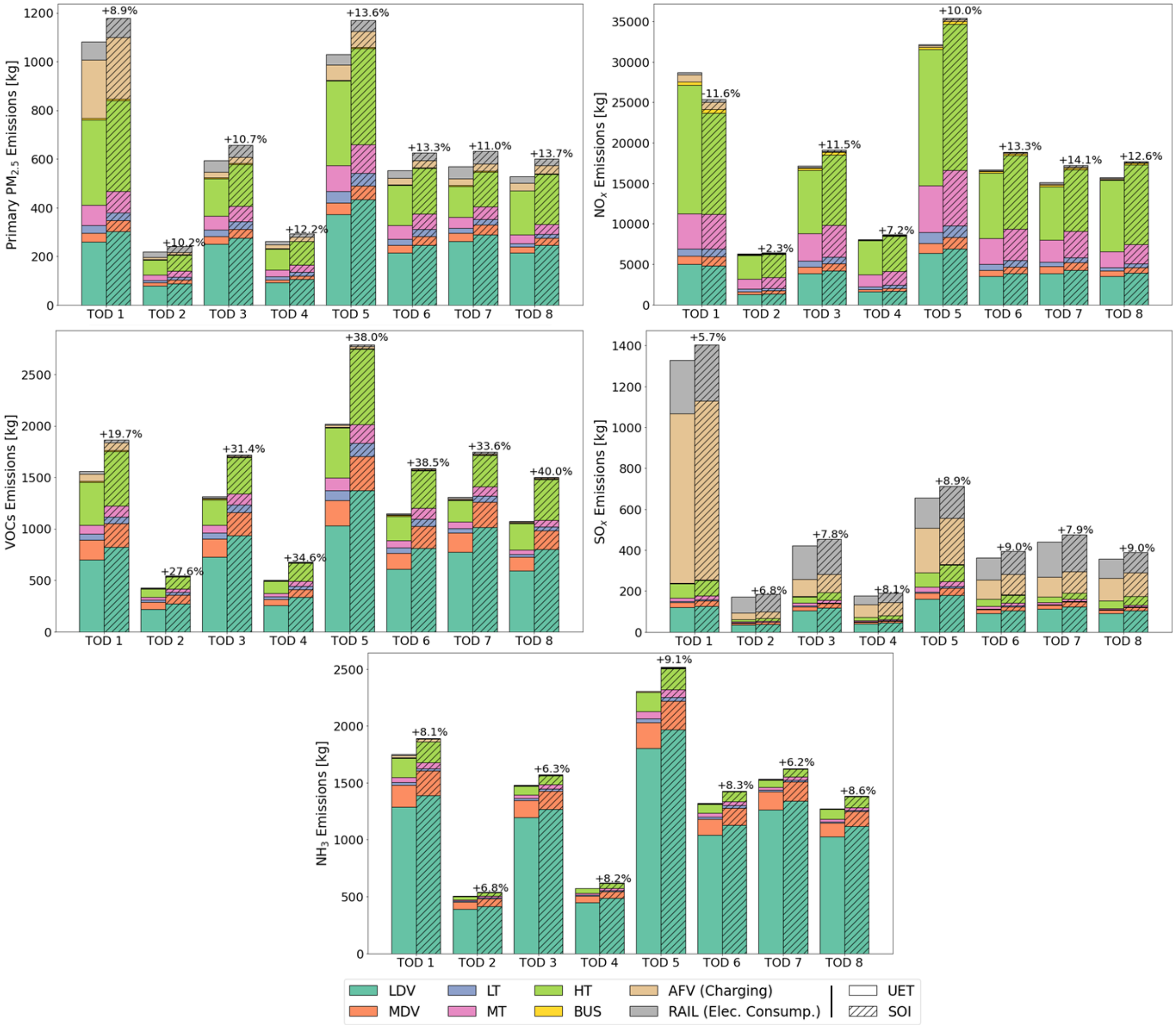

Figure S22: User-equilibrium for time (UET) and system optimal for intake (SOI) passenger emissions [kg] for primary $PM_{2.5}$, NO_x, SO_x, VOCs, and NH_3 disaggregated by time-of-day (TOD) period and vehicle user class. (LDV: Light-duty vehicles; MDV: Medium-duty vehicles; LT: Light-duty trucks; MT: Medium-duty trucks; HT: Heavy-duty trucks). (TOD 1: The ten-hour late evening-early morning off-peak period (8:00 p.m. to 6:00 a.m.); TOD 2: The shoulder hour preceding the AM peak hour (6:00 to 7:00 a.m.); TOD 3: The AM peak two hours (7:00 to 9:00 a.m.); TOD 4: The shoulder hour following the AM peak period (9:00 to 10:00 a.m.); TOD 5: A four-hour midday period (10:00 a.m. to 2:00 p.m.); TOD 6: The two-hour shoulder period

preceding the PM peak period (2:00 to 4:00 p.m.); TOD 7: The PM peak two hours (4:00 to 6:00 p.m.); TOD 8: The two-hour shoulder period following the PM peak period (6:00 to 8:00 p.m.)).

The rerouting principles of both assignments can be seen more clearly by analyzing the relationship between vehicle flow and emissions for both assignments. Scatter plots in **Figures S23-S30** show the difference in flow and emissions in the 1st and 3rd rows of each figure, respectively, between the two assignments as a function of link *iF* by subtracting UET values from SOI ones. The difference in flows and emissions on links with similar *iF*s from are then grouped and summed (shown in the 2nd and 4th rows) to better capture the general aggregate relationship between those metrics and link *iF*s relative to the two assignments. The high positive peaks for both flows and emissions show the large amount of flow and emissions that are being shifted onto low *iF* links, whereas the negative peaks show how the SOI moves emissions and flow away from higher *iF* links. The emissions and flows occurring on high *iF* links in the SOI are due to travel demand still needing to be met in the network and traveling on those links (which are mostly located in the Chicago urban center) is required since there are no alternatives.

The notable difference between peak and off-peak hours is that the relative magnitude of emission reductions is not as high during peak periods. Despite the SOI shifting a large amount of flow onto low *iF* links as shown by the grouped flow plots in row 2, the emission reductions are not as large, especially for NO_x and VOCs. This could be attributed to the fact that the SOI does lead to a larger increase in emissions during peak periods due to higher congestion as shown in **Figures S21** and **S22**. The higher congestion levels and number of trips taking place during peak hours can limit the options the SOI has for rerouting, this leading to higher congestion emissions. The high idle emissions in the SOI can also explain why the relative

magnitude of VOCs emission do not reduce as much. This further signifies how high congestion, in addition to having a high concentration of trips requiring travel through the Chicago urban center, limits the SOI from achieving higher exposure reduction.

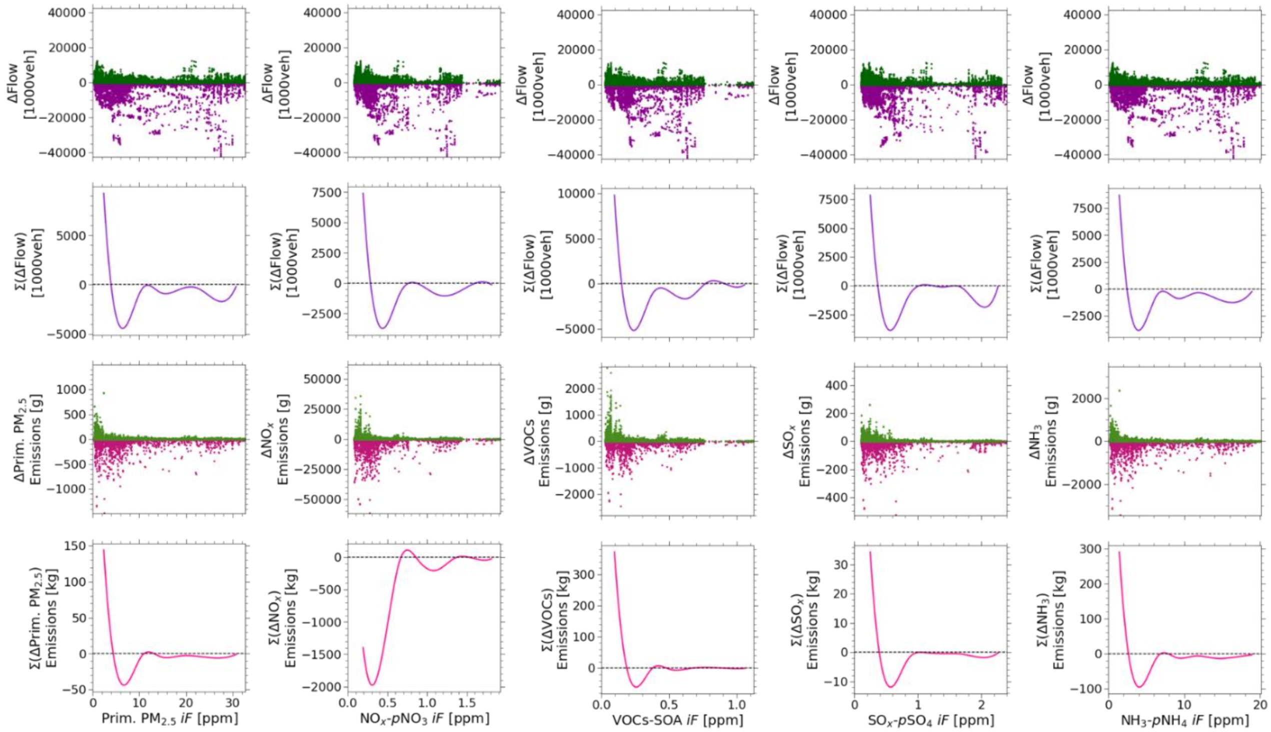

Figure S23: (1st Row) Scatter plots showing the relationship between difference in vehicle flow on every link within the network and the intake fraction (*iF*) of the link between the user equilibrium for time (UET) assignment and the system optimal for intake (SOI) assignments in TOD 1 for the five $PM_{2.5}$ pollutant species considered: Primary $PM_{2.5}$, pNO_3, SOA, pSO_4, and pNH_4. The difference is taken by subtracting UET flows from SOI flows (positive points show the increased flows in the SOI while the negative points show the reduce flows in the UET). (2nd Row) The difference in flows on links with similar *iF*s from the 1st row are aggregated and summed to show the general relationship between the difference in flows and the link *iF*s. (3rd & 4th Rows) Scatter and aggregated line plots that are similar to the top two rows, but they plot the difference in emissions between the five pollutants considered and iF for each link.

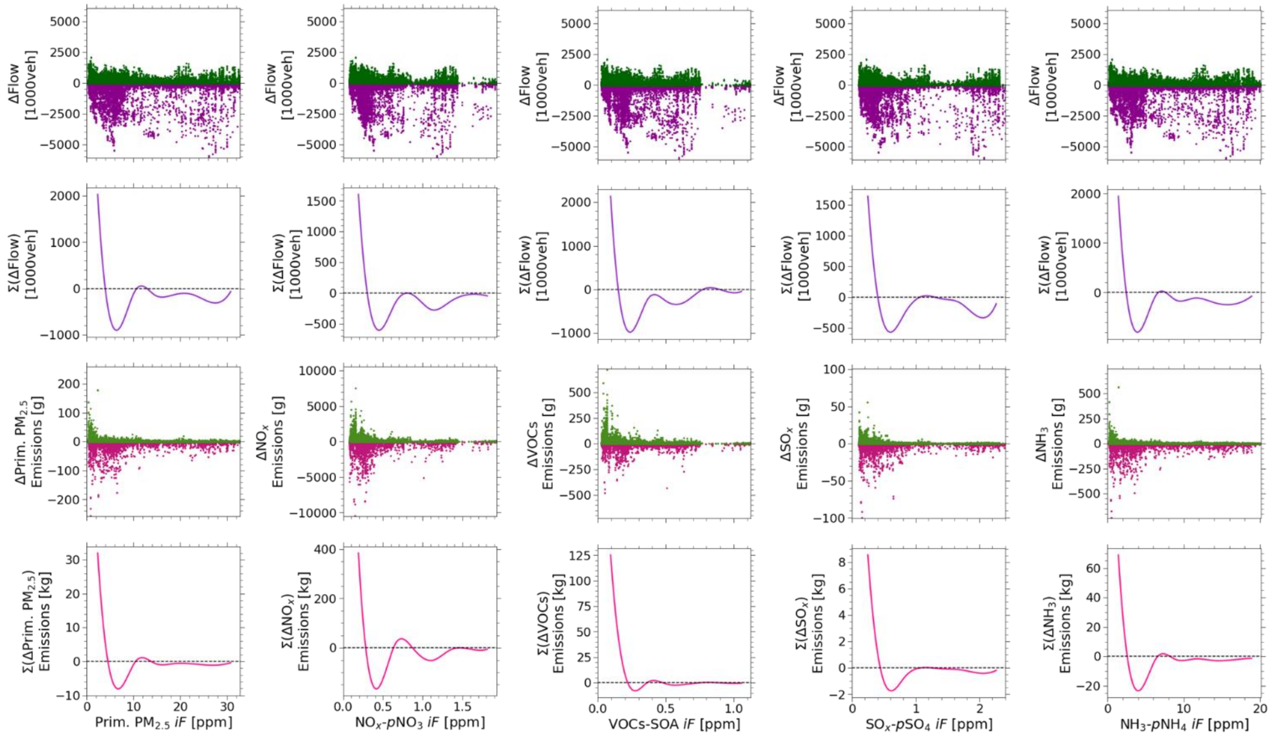

Figure S24: (1st Row) Scatter plots showing the relationship between difference in vehicle flow on every link within the network and the intake fraction (*iF*) of the link between the user equilibrium for time (UET) assignment and the system optimal for intake (SOI) assignments in TOD 2 for the five $PM_{2.5}$ pollutant species considered: Primary $PM_{2.5}$, pNO_3, SOA, pSO_4, and pNH_4. The difference is taken by subtracting UET flows from SOI flows (positive points show the increased flows in the SOI while the negative points show the reduce flows in the UET). (2nd Row) The difference in flows on links with similar *iF*s from the 1st row are aggregated and summed to show the general relationship between the difference in flows and the link *iF*s. (3rd & 4th Rows) Scatter and aggregated line plots that are similar to the top two rows, but they plot the difference in emissions between the five pollutants considered and *iF* for each link.

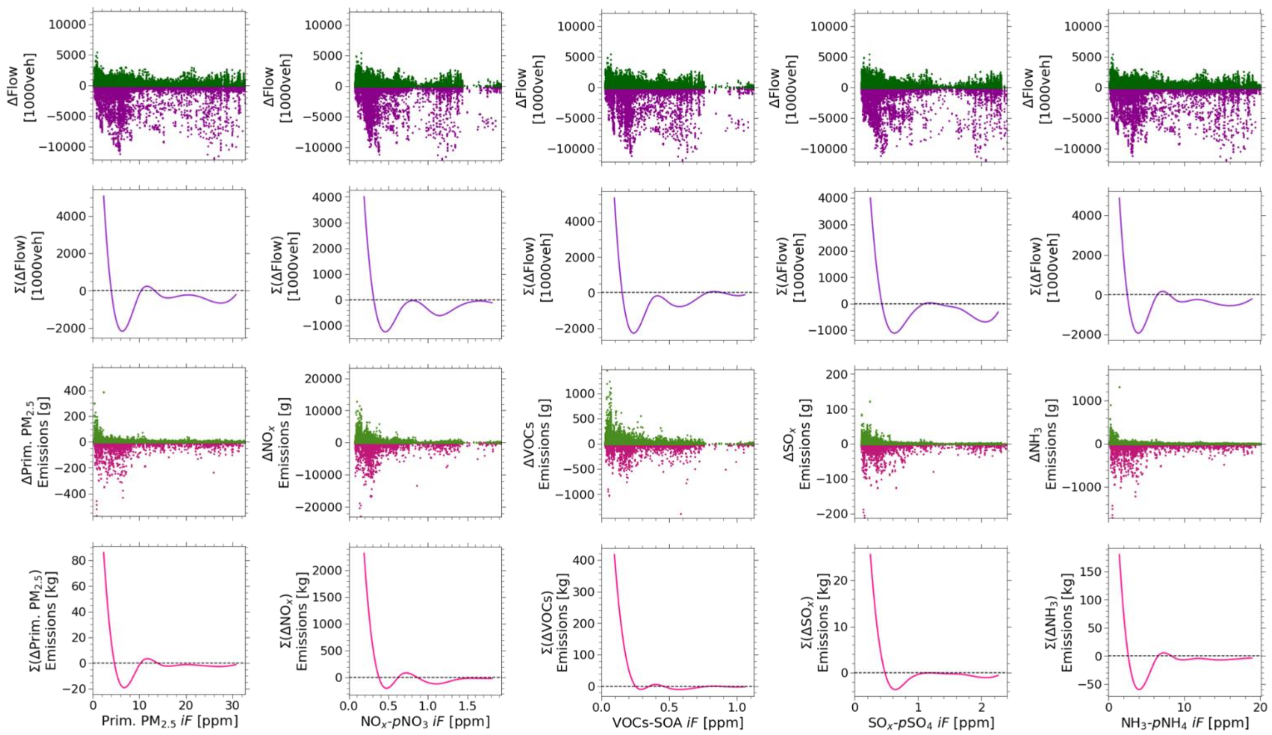

Figure S25: (1st Row) Scatter plots showing the relationship between difference in vehicle flow on every link within the network and the intake fraction (*iF*) of the link between the user equilibrium for time (UET) assignment and the system optimal for intake (SOI) assignments in TOD 3 for the five $PM_{2.5}$ pollutant species considered: Primary $PM_{2.5}$, *p*NO_3, SOA, *p*SO_4, and *p*NH_4. The difference is taken by subtracting UET flows from SOI flows (positive points show the increased flows in the SOI while the negative points show the reduce flows in the UET). (2nd Row) The difference in flows on links with similar *iF*s from the 1st row are aggregated and summed to show the general relationship between the difference in flows and the link *iF*s. (3rd & 4th Rows) Scatter and aggregated line plots that are similar to the top two rows, but they plot the difference in emissions between the five pollutants considered and *iF* for each link.

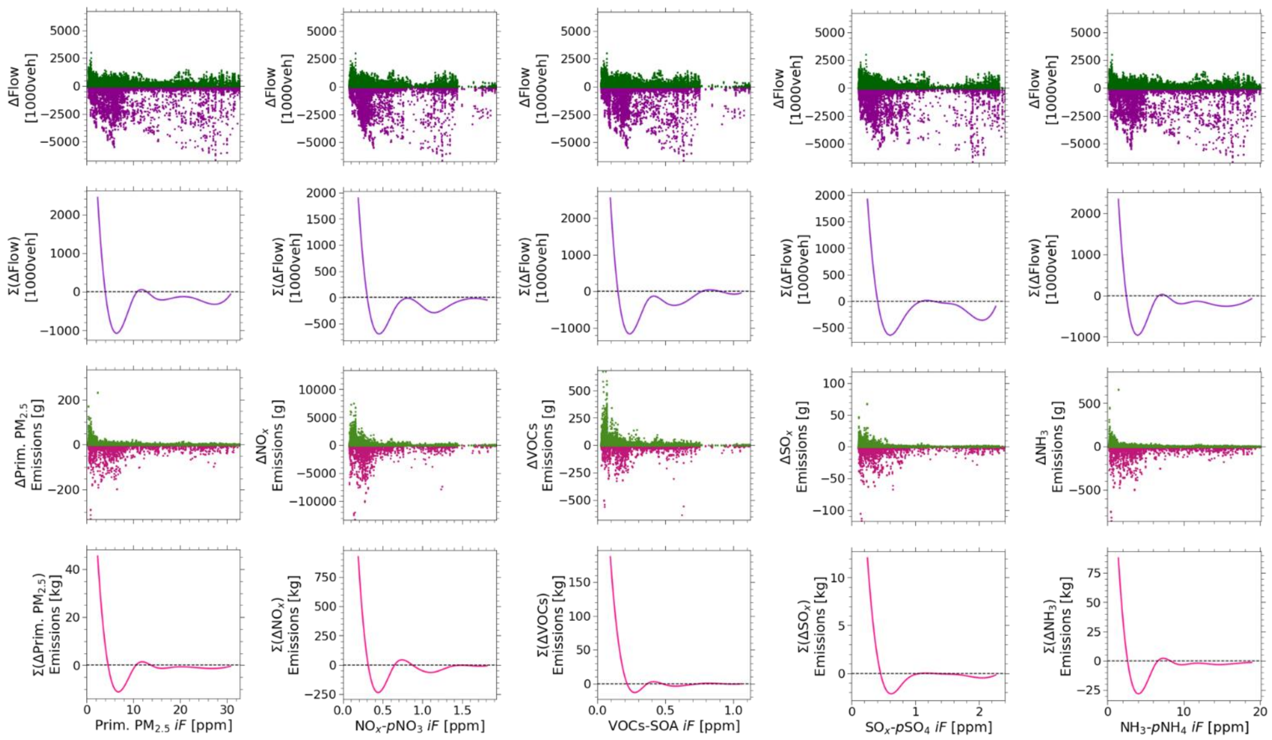

Figure S26: (1st Row) Scatter plots showing the relationship between difference in vehicle flow on every link within the network and the intake fraction (*iF*) of the link between the user equilibrium for time (UET) assignment and the system optimal for intake (SOI) assignments in TOD 4 for the five $PM_{2.5}$ pollutant species considered: Primary $PM_{2.5}$, pNO_3, SOA, pSO_4, and pNH_4. The difference is taken by subtracting UET flows from SOI flows (positive points show the increased flows in the SOI while the negative points show the reduce flows in the UET). (2nd Row) The difference in flows on links with similar *iF*s from the 1st row are aggregated and summed to show the general relationship between the difference in flows and the link *iF*s. (3rd & 4th Rows) Scatter and aggregated line plots that are similar to the top two rows, but they plot the difference in emissions between the five pollutants considered and *iF* for each link.

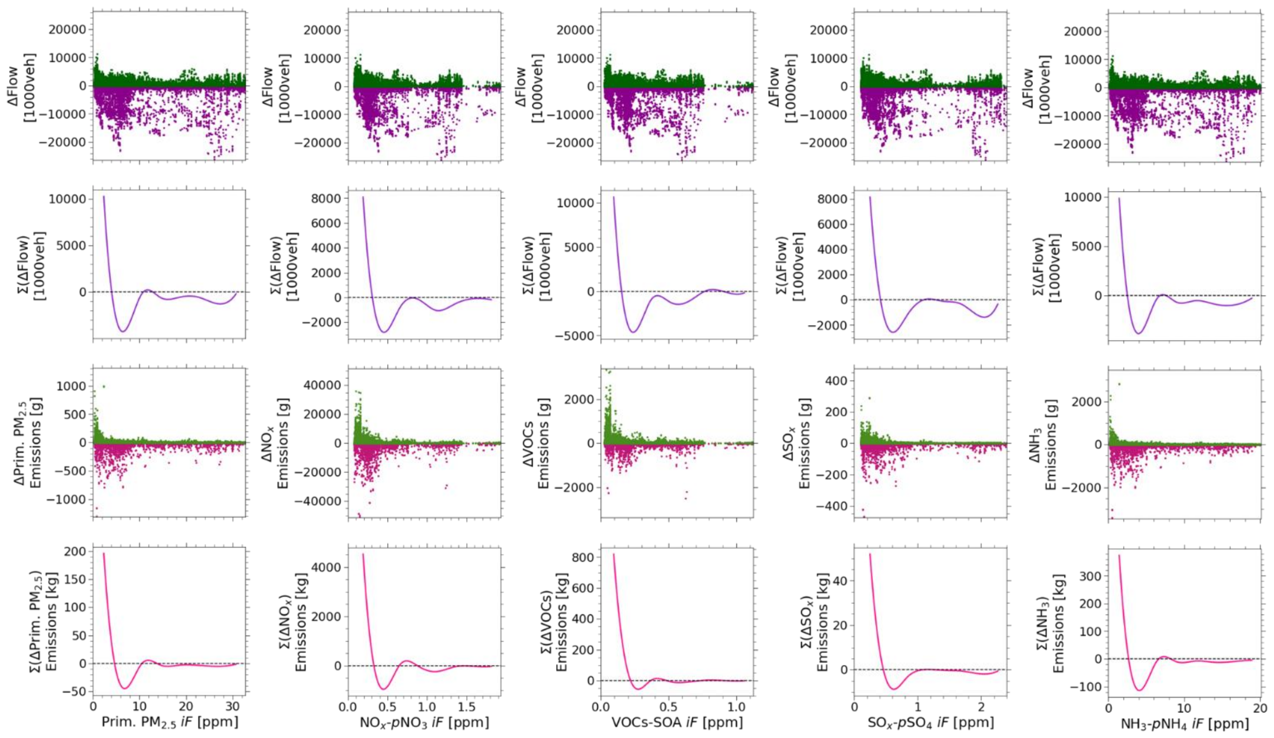

Figure S27: (1st Row) Scatter plots showing the relationship between difference in vehicle flow on every link within the network and the intake fraction (*iF*) of the link between the user equilibrium for time (UET) assignment and the system optimal for intake (SOI) assignments in TOD 5 for the five $PM_{2.5}$ pollutant species considered: Primary $PM_{2.5}$, *p*NO_3, SOA, *p*SO_4, and *p*NH_4. The difference is taken by subtracting UET flows from SOI flows (positive points show the increased flows in the SOI while the negative points show the reduce flows in the UET). (2nd Row) The difference in flows on links with similar *iF*s from the 1st row are aggregated and summed to show the general relationship between the difference in flows and the link *iF*s. (3rd & 4th Rows) Scatter and aggregated line plots that are similar to the top two rows, but they plot the difference in emissions between the five pollutants considered and *iF* for each link.

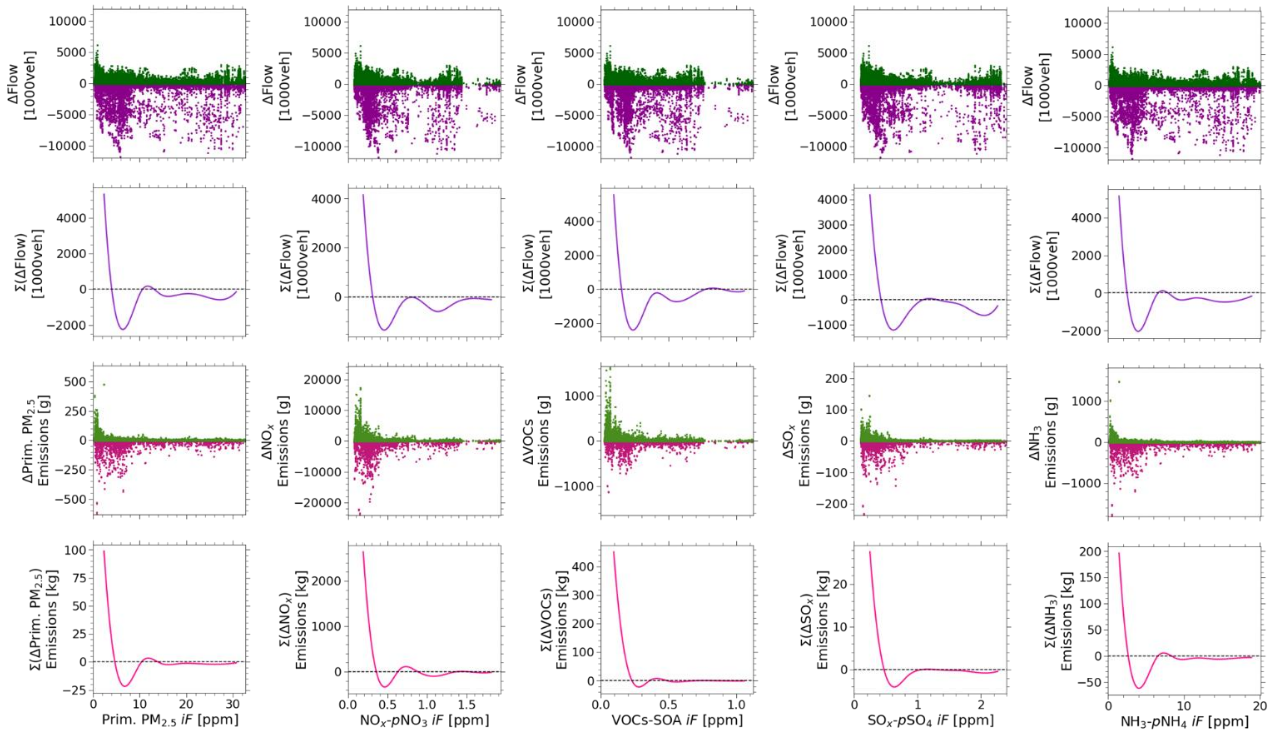

Figure S28: (1st Row) Scatter plots showing the relationship between difference in vehicle flow on every link within the network and the intake fraction (*iF*) of the link between the user equilibrium for time (UET) assignment and the system optimal for intake (SOI) assignments in TOD 6 for the five $PM_{2.5}$ pollutant species considered: Primary $PM_{2.5}$, pNO_3, SOA, pSO_4, and pNH_4. The difference is taken by subtracting UET flows from SOI flows (positive points show the increased flows in the SOI while the negative points show the reduce flows in the UET). (2nd Row) The difference in flows on links with similar *iF*s from the 1st row are aggregated and summed to show the general relationship between the difference in flows and the link *iF*s. (3rd & 4th Rows) Scatter and aggregated line plots that are similar to the top two rows, but they plot the difference in emissions between the five pollutants considered and *iF* for each link.

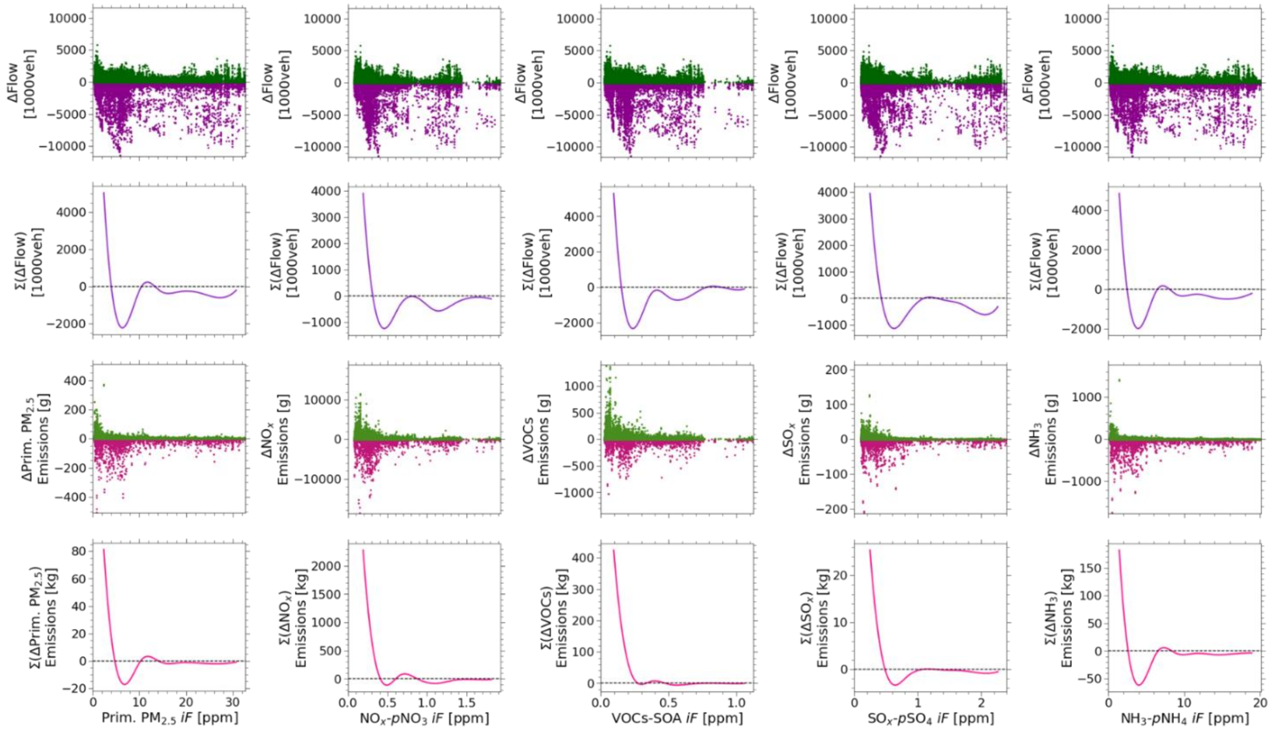

Figure S29: (1st Row) Scatter plots showing the relationship between difference in vehicle flow on every link within the network and the intake fraction (*iF*) of the link between the user equilibrium for time (UET) assignment and the system optimal for intake (SOI) assignments in TOD 7 for the five $PM_{2.5}$ pollutant species considered: Primary $PM_{2.5}$, pNO_3, SOA, pSO_4, and pNH_4. The difference is taken by subtracting UET flows from SOI flows (positive points show the increased flows in the SOI while the negative points show the reduce flows in the UET). (2nd Row) The difference in flows on links with similar *iF*s from the 1st row are aggregated and summed to show the general relationship between the difference in flows and the link *iF*s. (3rd & 4th Rows) Scatter and aggregated line plots that are similar to the top two rows, but they plot the difference in emissions between the five pollutants considered and iF for each link.

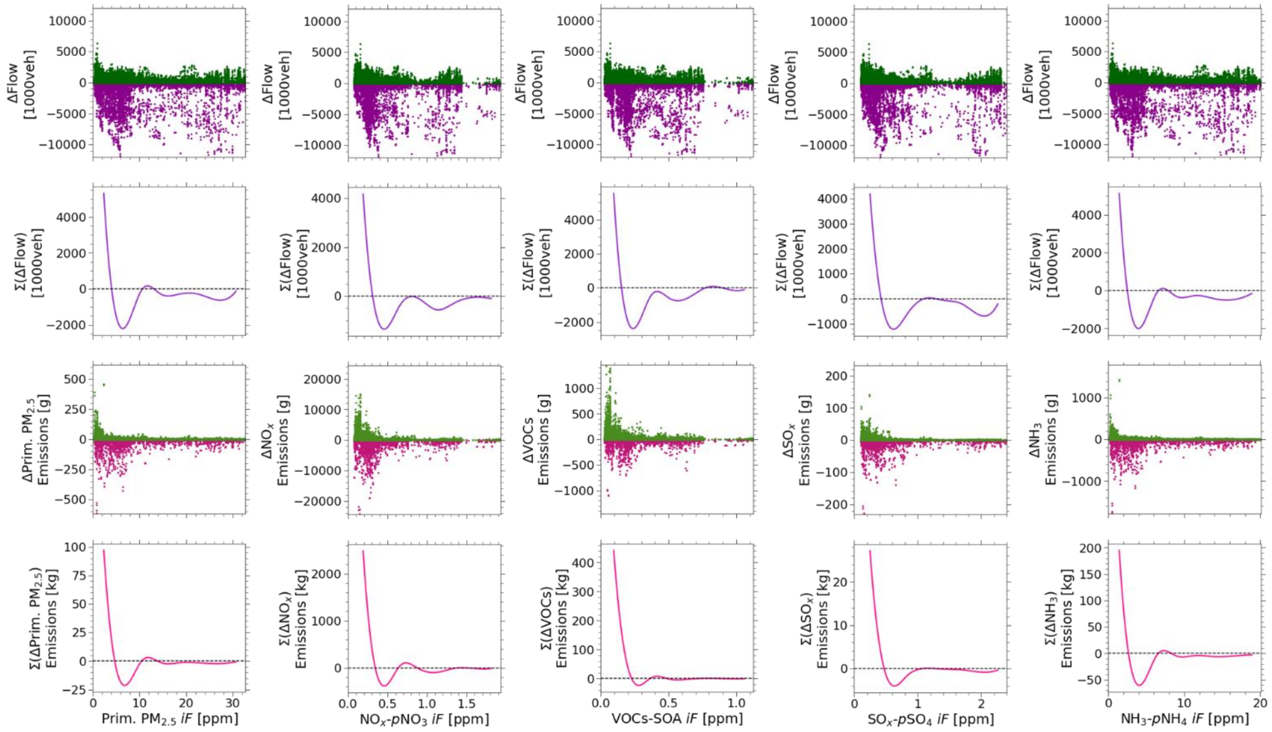

Figure S30: (1st Row) Scatter plots showing the relationship between difference in vehicle flow on every link within the network and the intake fraction (*iF*) of the link between the user equilibrium for time (UET) assignment and the system optimal for intake (SOI) assignments in TOD 8 for the five $PM_{2.5}$ pollutant species considered: Primary $PM_{2.5}$, pNO_3, SOA, pSO_4, and pNH_4. The difference is taken by subtracting UET flows from SOI flows (positive points show the increased flows in the SOI while the negative points show the reduce flows in the UET). (2nd Row) The difference in flows on links with similar *iF*s from the 1st row are aggregated and summed to show the general relationship between the difference in flows and the link *iF*s. (3rd & 4th Rows) Scatter and aggregated line plots that are similar to the top two rows, but they plot the difference in emissions between the five pollutants considered and *iF* for each link.

2.3 Exposure by $PM_{2.5}$ Species

In terms of the $PM_{2.5}$ intake attributable to each of the five $PM_{2.5}$ species for both assignments, NO_x yields the highest contributions at approximately 42%. This is followed by primary $PM_{2.5}$ at about 32%, while NH_3, VOCs, and SO_x contribute to about 22%, 2%, and <1%, respectively. The high proportion of intake due to primary $PM_{2.5}$ is expected given that this form of $PM_{2.5}$ leads to exposures soon after it is emitted from vehicles in addition to the proximity of roadways to people (i.e., high *iF*). Primary $PM_{2.5}$ intake due to brake ware and tire wear are about 25% and 50% for LDVs and MDVs but are about 60% and 5% for trucks, respectively. The remainder is due to primary $PM_{2.5}$ tailpipe emissions. High exposure to secondary $PM_{2.5}$ from NO_x emissions can be attributed to the large amount of NO_x emitted from on-road vehicles (especially trucks) despite having lower *iF*s given that it is a secondary pollutant. Intake from EGU emissions only makes up 1% − 2% of all intake for NO_x, primary $PM_{2.5}$, NH_3, and VOCs, but about half of intake from SO_x due to the high SO_x emissions from coal-based EGUs. However, intake from SO_x emissions is generally small for the baseline electricity use case.

2.4 Emissions and $PM_{2.5}$ Exposure Results for All Strategies

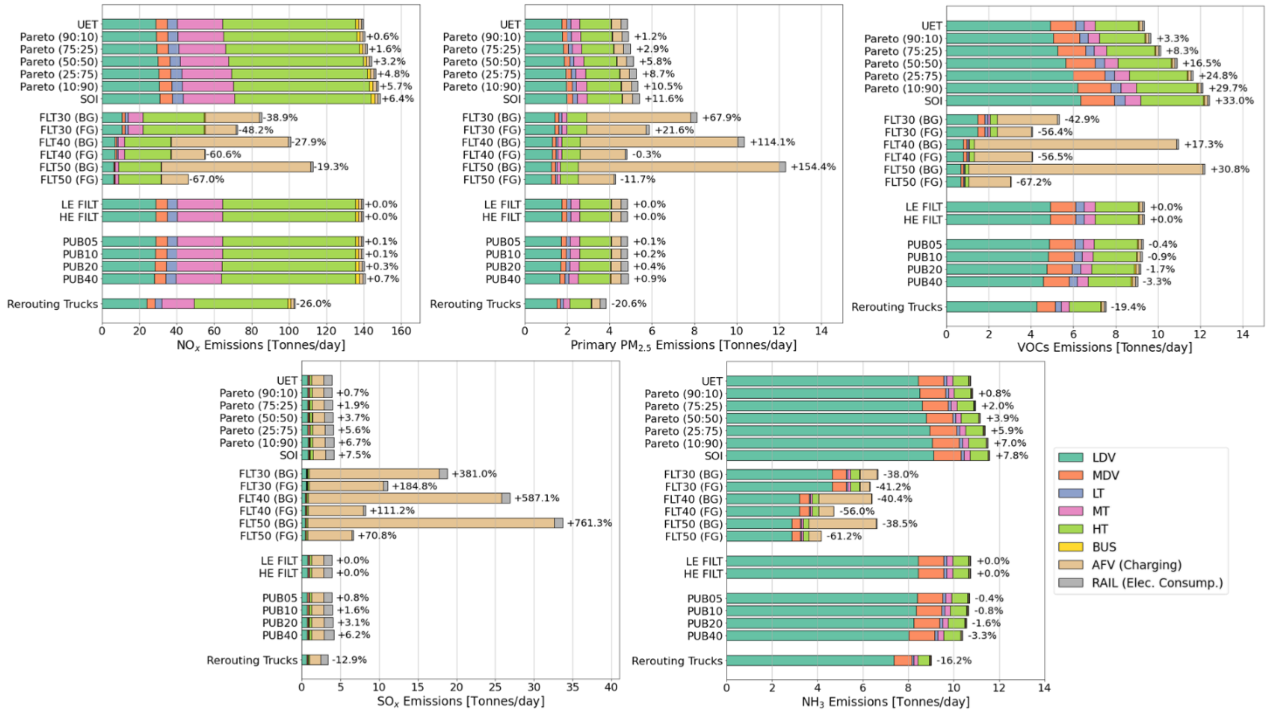

Figure S31: NO_x, primary $PM_{2.5}$, VOCs, SO_x, and NH_3 emissions [tonnes/day] disaggregated by vehicle user class for different strategies and scenarios assessed. (LDV: Light-duty vehicles; MDV: Medium-duty vehicles; LT: Light-duty trucks; MT: Medium-duty trucks; HT: Heavy-duty trucks).

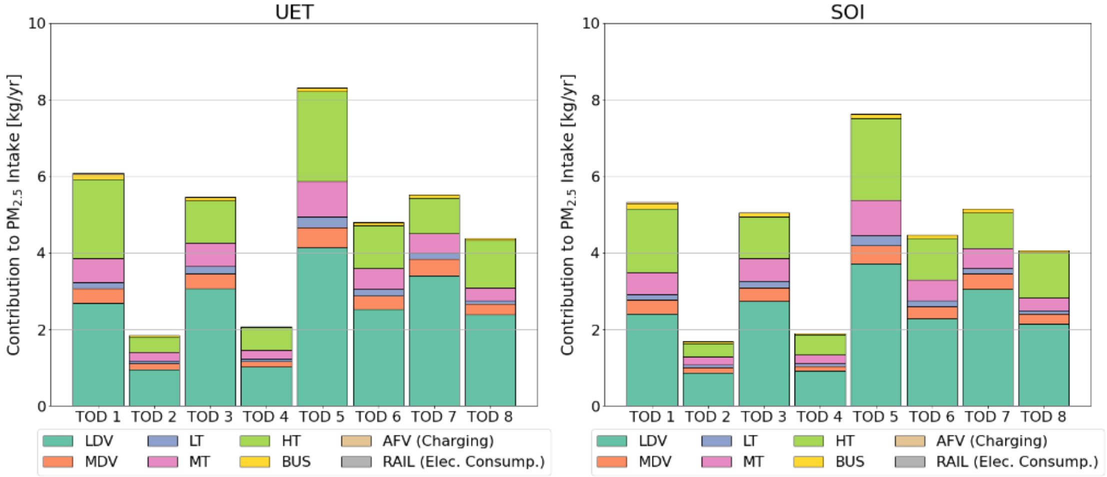

Figure S32: $PM_{2.5}$ intake contribution disaggregated by time-of-day (TOD) period and vehicle user class type for the user-equilibrium for time (UET) and system optimal for intake (SOI) scenarios. (LDV: Light-duty vehicles; MDV: Medium-duty vehicles; LT: Light-duty trucks; MT: Medium-duty trucks; HT: Heavy-duty trucks). (TOD 1: The ten-hour late evening-early morning off-peak period (8:00 p.m. to 6:00 a.m.); TOD 2: The shoulder hour preceding the AM peak hour (6:00 to 7:00 a.m.); TOD 3: The AM peak two hours (7:00 to 9:00 a.m.); TOD 4: The shoulder hour following the AM peak period (9:00 to 10:00 a.m.); TOD 5: A four-hour midday period (10:00 a.m. to 2:00 p.m.); TOD 6: The two-hour shoulder period preceding the PM peak period (2:00 to 4:00 p.m.); TOD 7: The PM peak two hours (4:00 to 6:00 p.m.); TOD 8: The two-hour shoulder period following the PM peak period (6:00 to 8:00 p.m.)).

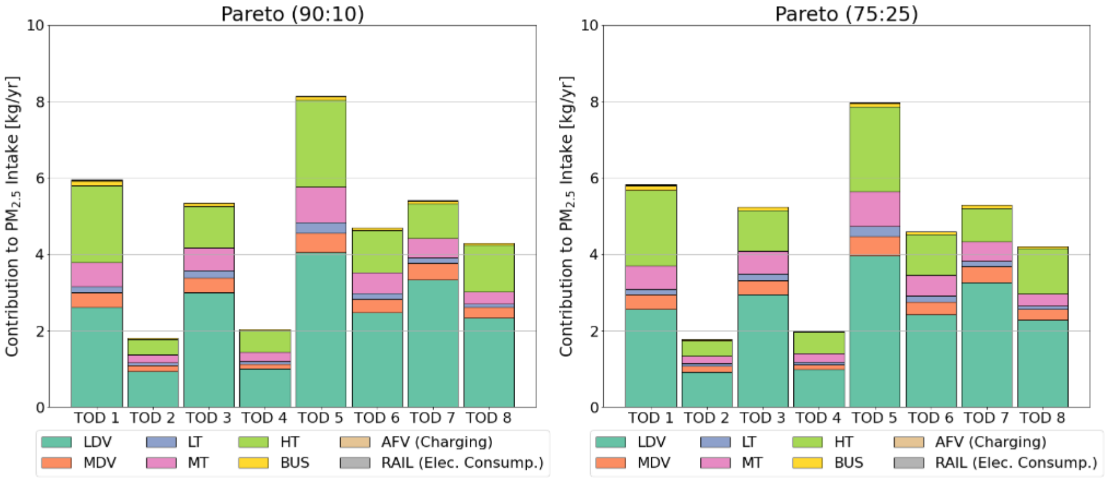

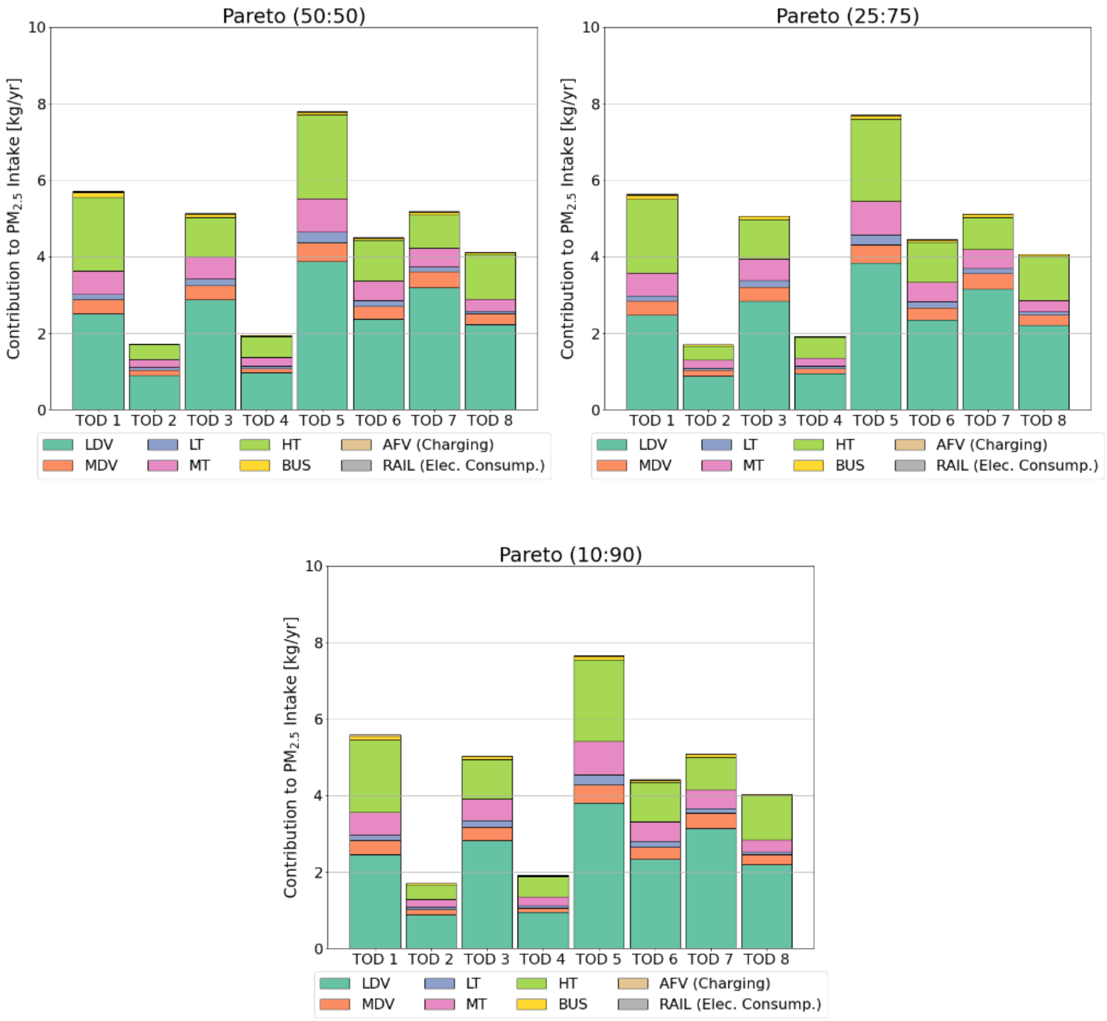

Figure S33: $PM_{2.5}$ intake contribution disaggregated by time-of-day period (TOD) and vehicle user class type for the bi-objective optimization scenarios. (LDV: Light-duty vehicles; MDV: Medium-duty vehicles; LT: Light-duty trucks; MT: Medium-duty trucks; HT: Heavy-duty trucks). (TOD 1: The ten-hour late evening-early morning off-peak period (8:00 p.m. to 6:00 a.m.); TOD 2: The shoulder hour preceding the AM peak hour (6:00 to 7:00 a.m.); TOD 3: The AM peak two hours (7:00 to 9:00 a.m.); TOD 4: The shoulder hour following the AM peak period (9:00 to 10:00 a.m.); TOD 5: A four-hour midday period (10:00 a.m. to 2:00 p.m.); TOD 6: The two-hour shoulder period preceding the PM peak period (2:00 to 4:00 p.m.); TOD 7: The

PM peak two hours (4:00 to 6:00 p.m.); TOD 8: The two-hour shoulder period following the PM peak period (6:00 to 8:00 p.m.)).

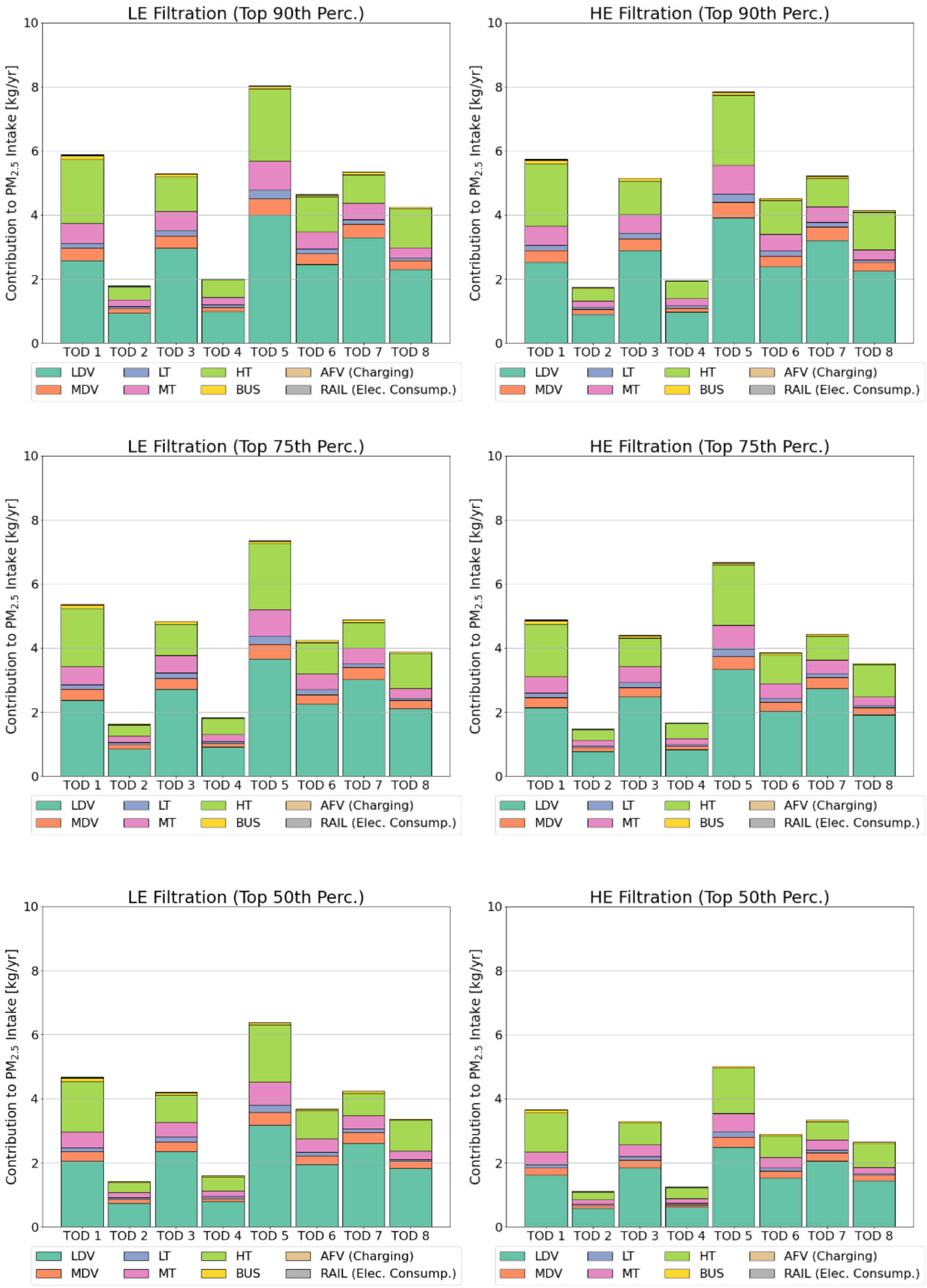

Figure S34: $PM_{2.5}$ intake contribution disaggregated by time-of-day (TOD) period and vehicle user class type for the particle filtration scenarios. (LDV: Light-duty vehicles; MDV: Medium-duty vehicles; LT: Light-duty trucks; MT: Medium-duty trucks; HT: Heavy-duty trucks). (TOD 1: The ten-hour late evening-early morning off-peak period (8:00 p.m. to 6:00 a.m.); TOD 2: The shoulder hour preceding the AM peak hour (6:00 to 7:00 a.m.); TOD 3: The AM peak two hours (7:00 to 9:00 a.m.); TOD 4: The shoulder hour following the AM peak period (9:00 to 10:00 a.m.); TOD 5: A four-hour midday period (10:00 a.m. to 2:00 p.m.); TOD 6: The two-hour shoulder period preceding the PM peak period (2:00 to 4:00 p.m.); TOD 7: The PM peak two hours (4:00 to 6:00 p.m.); TOD 8: The two-hour shoulder period following the PM peak period (6:00 to 8:00 p.m.)).

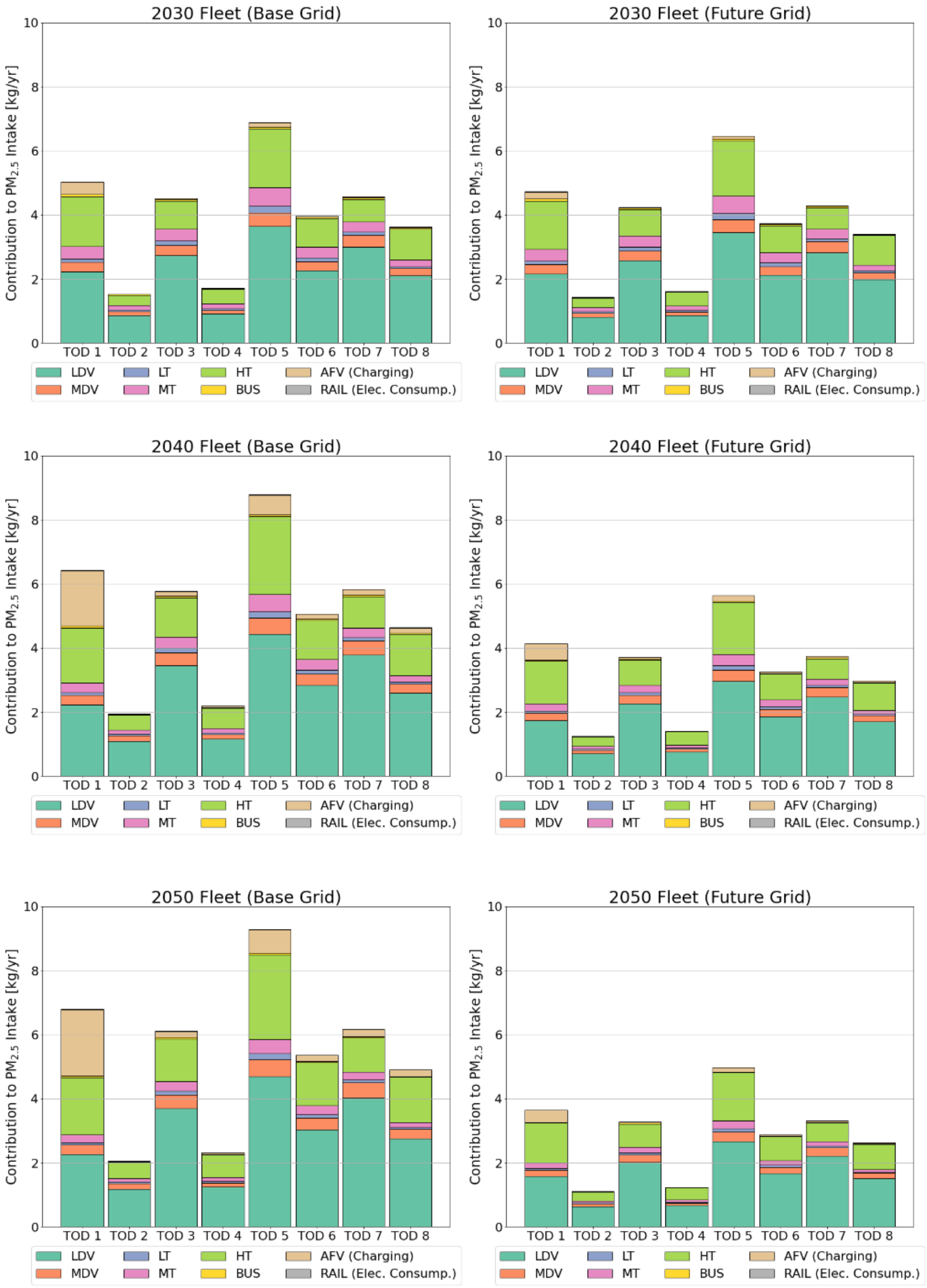

Figure S35: $PM_{2.5}$ intake contribution disaggregated by time-of-day period (TOD) and vehicle user class type for the particle filtration scenarios. (LDV: Light-duty vehicles; MDV: Medium-

duty vehicles; LT: Light-duty trucks; MT: Medium-duty trucks; HT: Heavy-duty trucks). (TOD 1: The ten-hour late evening-early morning off-peak period (8:00 p.m. to 6:00 a.m.); TOD 2: The shoulder hour preceding the AM peak hour (6:00 to 7:00 a.m.); TOD 3: The AM peak two hours (7:00 to 9:00 a.m.); TOD 4: The shoulder hour following the AM peak period (9:00 to 10:00 a.m.); TOD 5: A four-hour midday period (10:00 a.m. to 2:00 p.m.); TOD 6: The two-hour shoulder period preceding the PM peak period (2:00 to 4:00 p.m.); TOD 7: The PM peak two hours (4:00 to 6:00 p.m.); TOD 8: The two-hour shoulder period following the PM peak period (6:00 to 8:00 p.m.)).

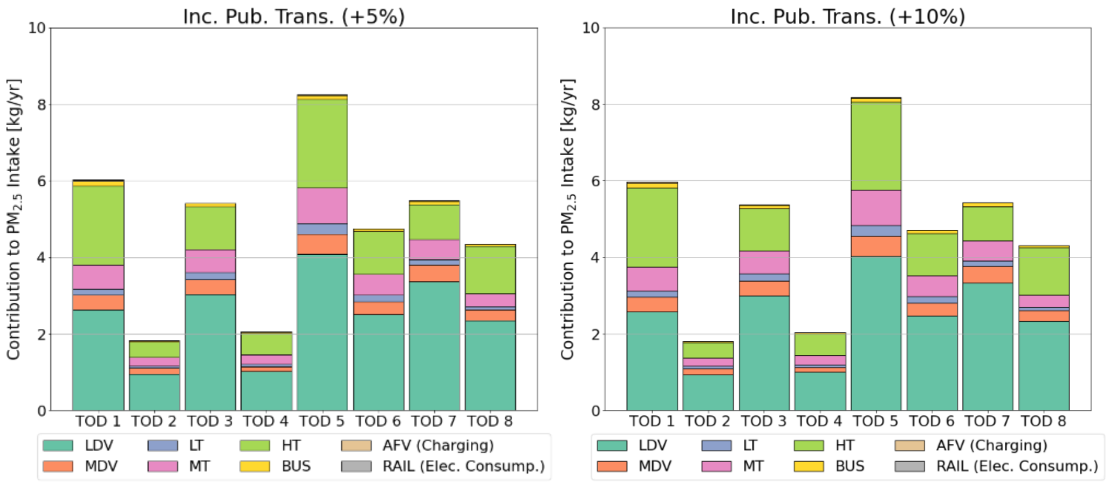

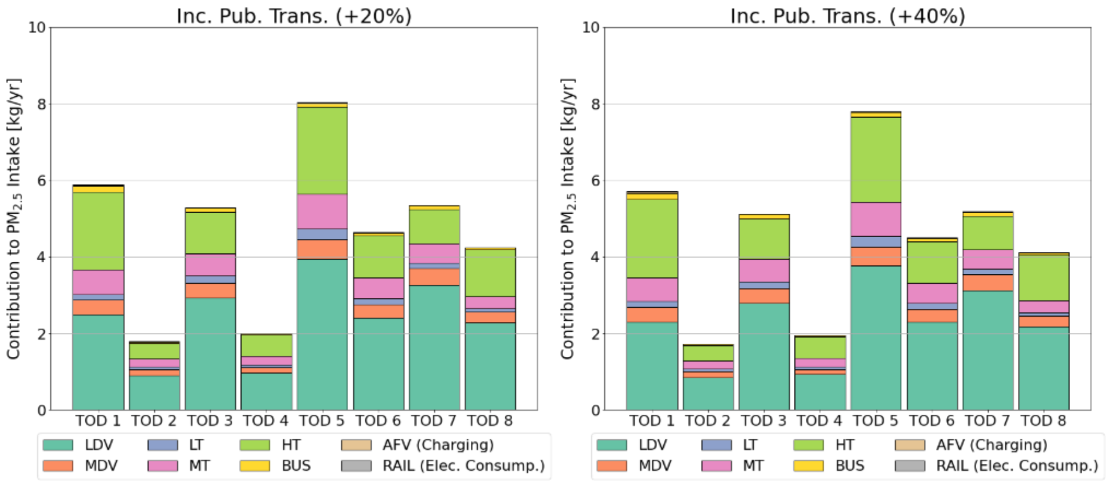

Figure S36: $PM_{2.5}$ intake contribution disaggregated by time-of-day (TOD) period and vehicle user class type for the public transportation scenarios. (LDV: Light-duty vehicles; MDV: Medium-duty vehicles; LT: Light-duty trucks; MT: Medium-duty trucks; HT: Heavy-duty trucks). (TOD 1: The ten-hour late evening-early morning off-peak period (8:00 p.m. to 6:00 a.m.); TOD 2: The shoulder hour preceding the AM peak hour (6:00 to 7:00 a.m.); TOD 3: The AM peak two hours (7:00 to 9:00 a.m.); TOD 4: The shoulder hour following the AM peak period (9:00 to 10:00 a.m.); TOD 5: A four-hour midday period (10:00 a.m. to 2:00 p.m.); TOD 6: The two-hour shoulder period preceding the PM peak period (2:00 to 4:00 p.m.); TOD 7: The PM peak two hours (4:00 to 6:00 p.m.); TOD 8: The two-hour shoulder period following the PM peak period (6:00 to 8:00 p.m.)).

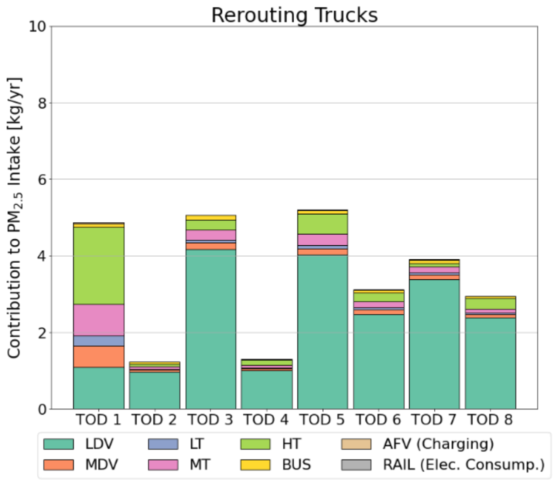

Figure S37: $PM_{2.5}$ intake contribution disaggregated by time-of-day (TOD) period and vehicle user class type for the truck rerouting scenario. (LDV: Light-duty vehicles; MDV: Medium-duty vehicles; LT: Light-duty trucks; MT: Medium-duty trucks; HT: Heavy-duty trucks). (TOD 1: The ten-hour late evening-early morning off-peak period (8:00 p.m. to 6:00 a.m.); TOD 2: The shoulder hour preceding the AM peak hour (6:00 to 7:00 a.m.); TOD 3: The AM peak two hours (7:00 to 9:00 a.m.); TOD 4: The shoulder hour following the AM peak period (9:00 to 10:00 a.m.); TOD 5: A four-hour midday period (10:00 a.m. to 2:00 p.m.); TOD 6: The two-hour shoulder period preceding the PM peak period (2:00 to 4:00 p.m.); TOD 7: The PM peak two hours (4:00 to 6:00 p.m.); TOD 8: The two-hour shoulder period following the PM peak period (6:00 to 8:00 p.m.)).

2.5 Exposure-Based Tolls

As mentioned, to achieve bi-objective based routing for travel time and exposure, one can establish road tolls as a means to help shift traffic away from high *iF* links to reduce exposure for the most impacted populations. The relative weighting given to the SOI can be used to determine the magnitude of the tolls as well as the links in which they are applied. **Figures S38 – S42** show

how tolls can be applied to different vehicle categories assuming the full exposure externality is to be internalized by the vehicle users. Tolls increase for higher polluting vehicle categories and are highest for heavy-duty trucks since they cause more damages per unit of emissions. Any links that lie within the bottom 75th of toll costs are assumed to carry no tolls. Furthermore, two sets of toll-pricing levels were estimated, one which assumes low-traffic conditions and the other assumes congestion levels are high. This is because the level of congestion affects the amount of emissions per distance travelled, and high congestion conditions contribute to higher emissions. Therefore, higher toll prices are set when congestion levels are high. The highest tolls are set for heavy-duty trucks. Heavy-duty truck tolls range from $0.22 – $4.0 during peak hours but can be as high as $1.4 – $30 during congestion periods. The largest concentrations of high-price toll links are located within the Chicago urban center due to the high *iF* values of those links. High tolls are also set on network freeways/expressways. The remaining tolls are scattered around the network limits, specifically where some larger cities are found such as Rockford in the northwest and Milwaukee in the north. **Figures S38 – S42** show that there is a correlation between the links which have no tolls and the links in which much of the traffic flow was rerouted to in the SOI in **Figures S13 – S20**. Conversely, much of the high toll links were the ones which the SOI rerouted traffic flow away from.

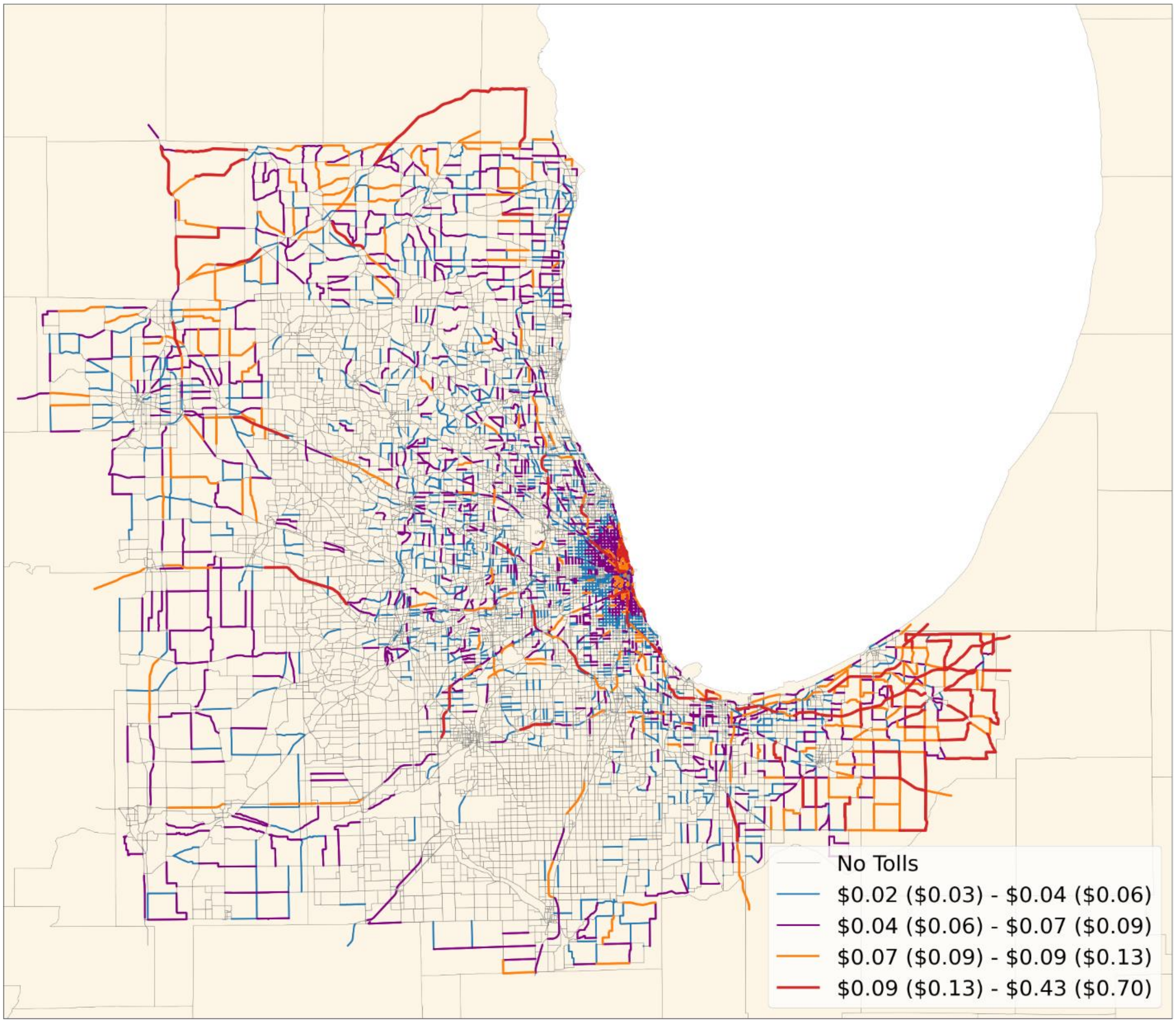

Figure S38: Exposure-based road tolls [$] for based on link *iF*s and light-duty vehicle emissions. Values not in parentheses are tolls set for off-peak hour periods, while values in parentheses show tolls for peak hour periods.

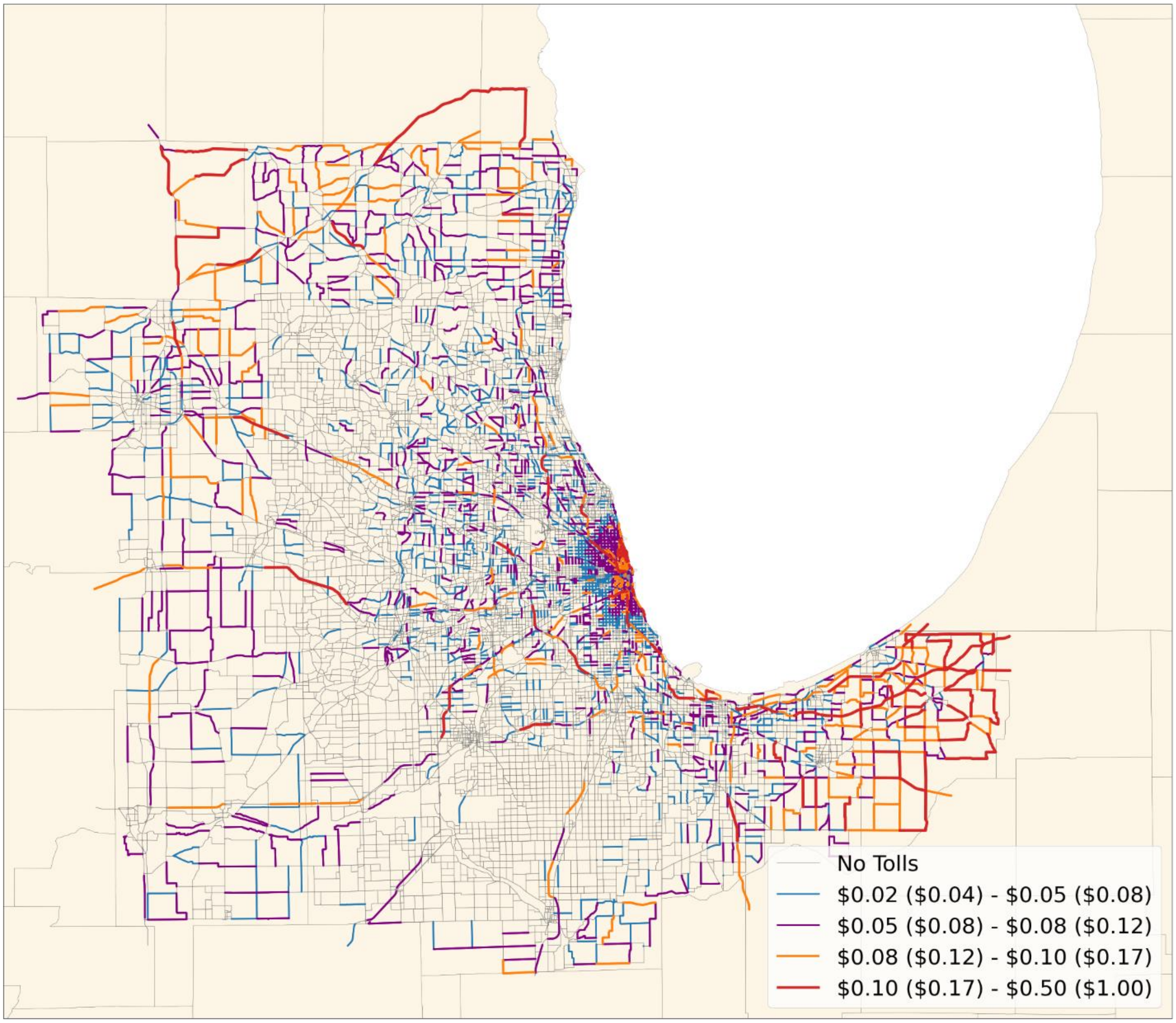

Figure S39: Exposure-based road tolls [$] for based on link *iF*s and medium-duty vehicle emissions. Values not in parentheses are tolls set for off-peak hour periods, while values in parentheses show tolls for peak hour periods.

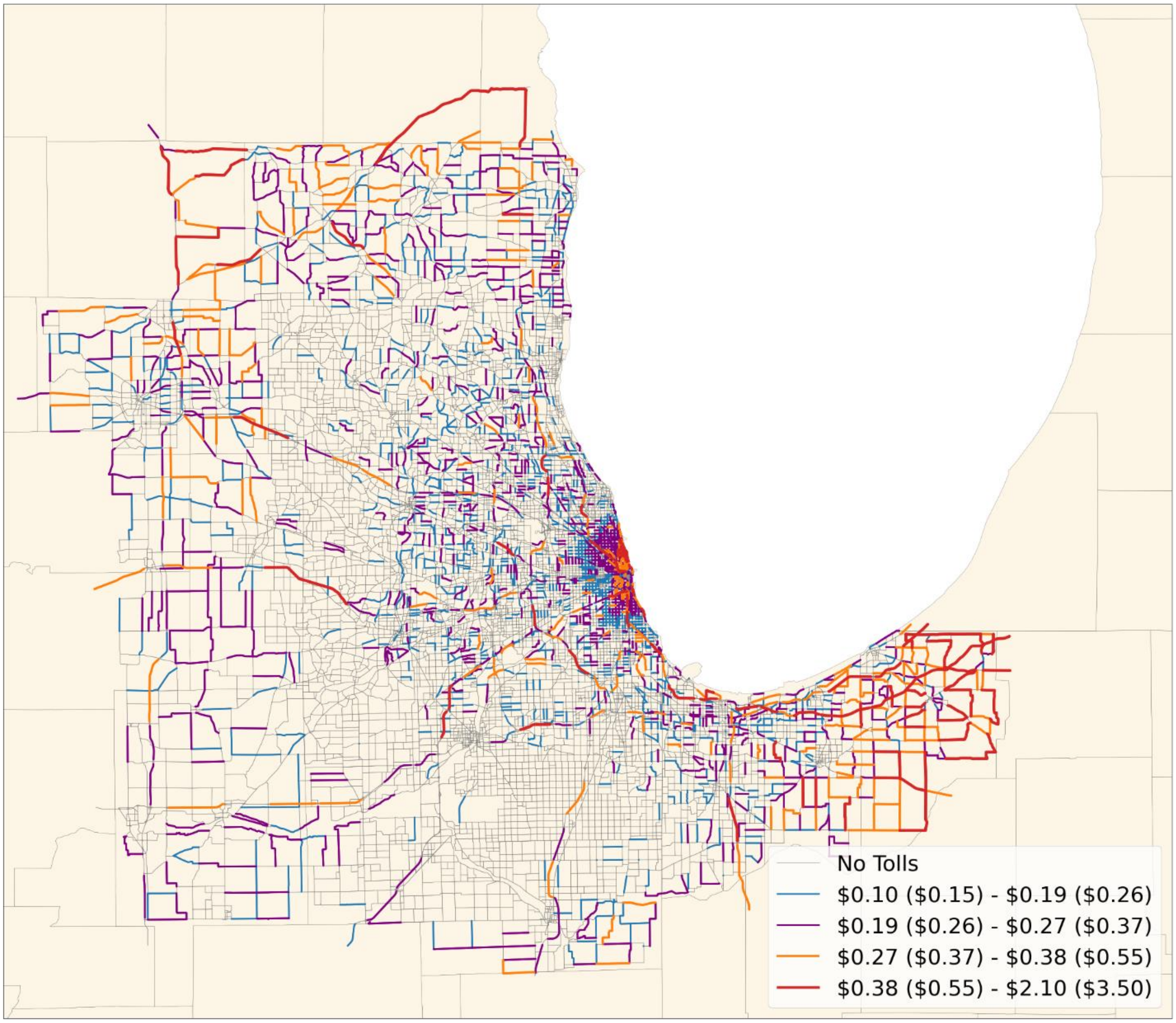

Figure S40: Exposure-based road tolls [$] for based on link *iF*s and light-duty truck emissions. Values not in parentheses are tolls set for off-peak hour periods, while values in parentheses show tolls for peak hour periods.

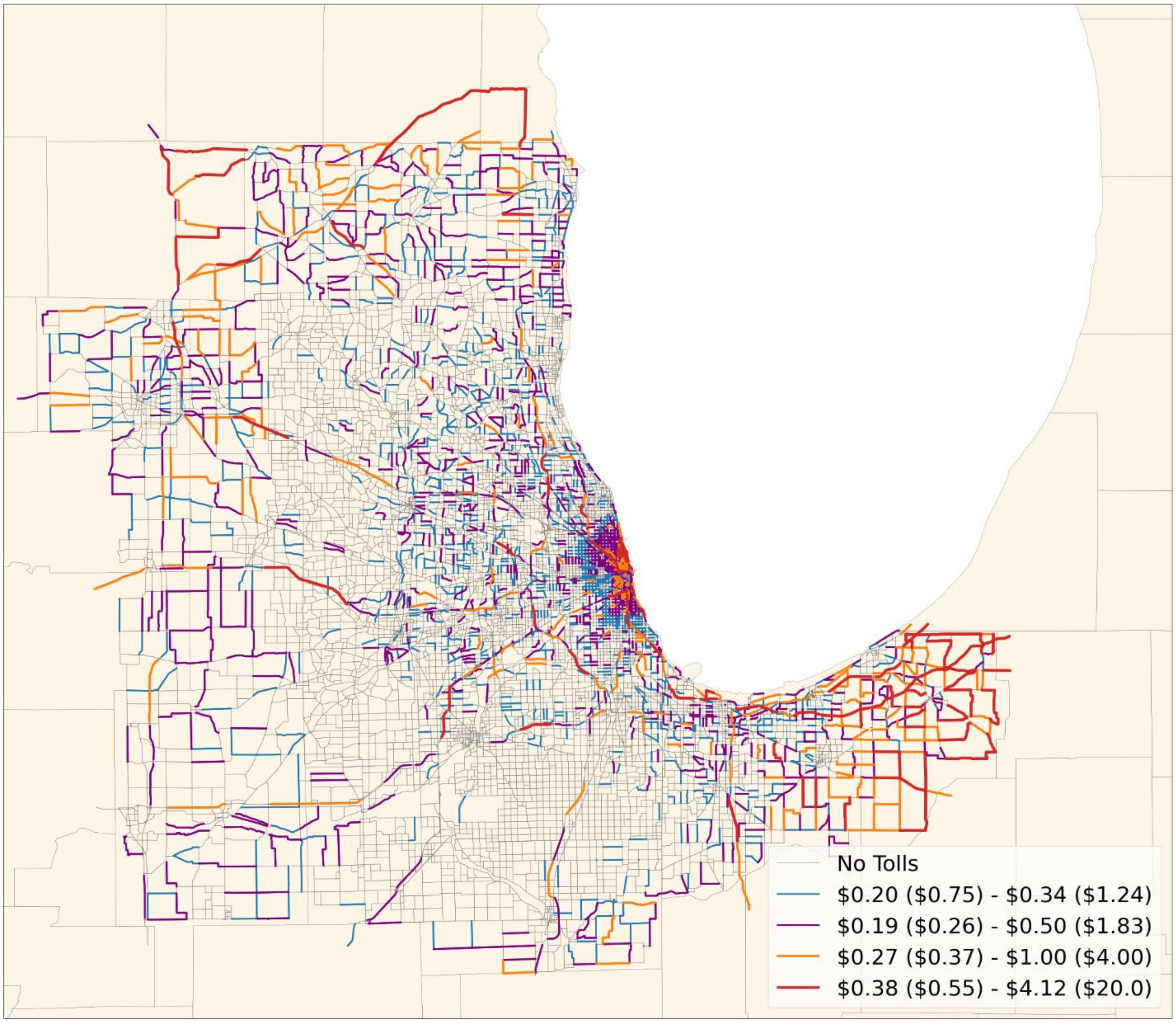

Figure S41: Exposure-based road tolls [$] for based on link *iF*s and medium-duty truck emissions. Values not in parentheses are tolls set for off-peak hour periods, while values in parentheses show tolls for peak hour periods.

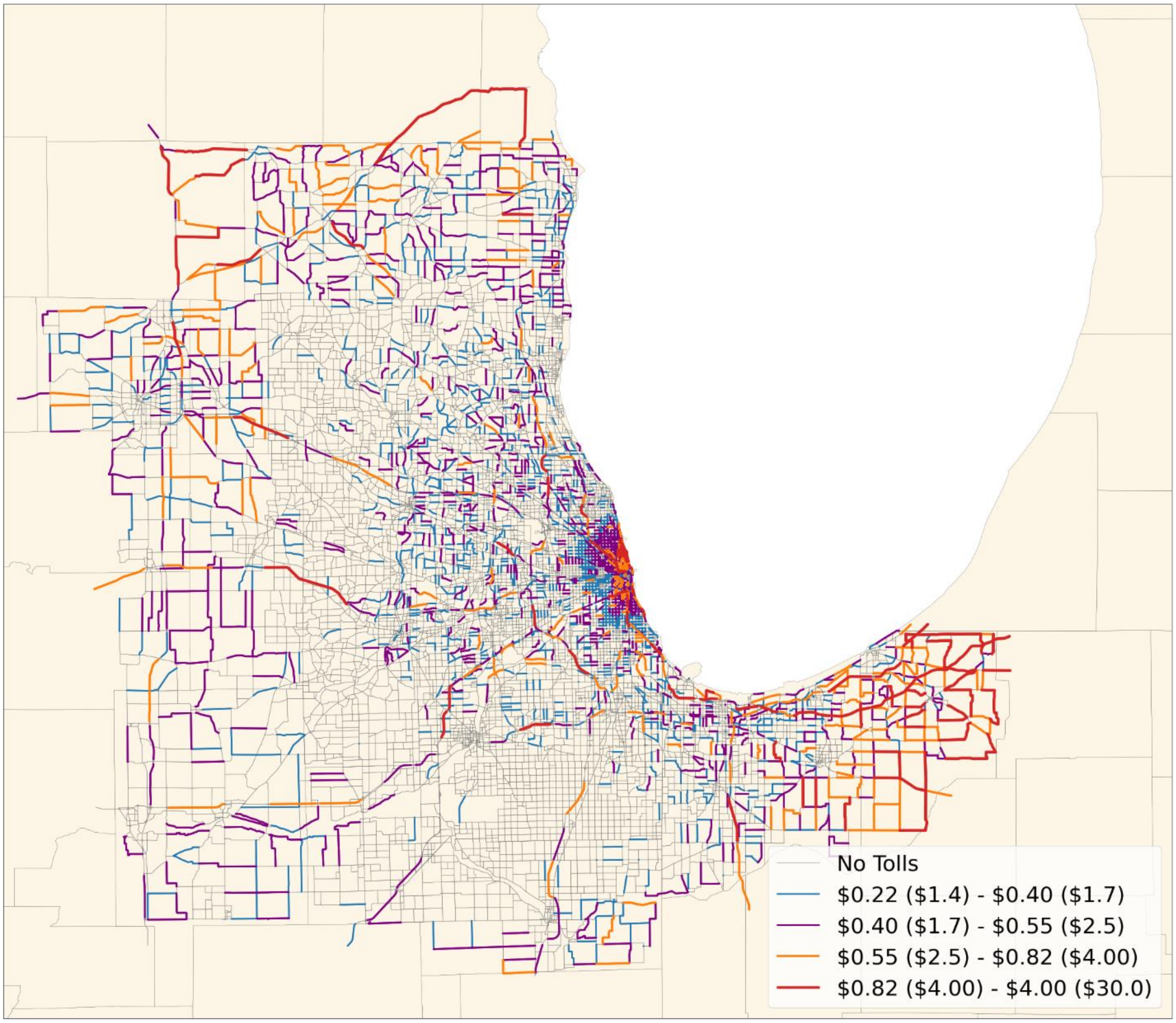

Figure S42: Exposure-based road tolls [$] for based on link *iF*s and heavy-duty truck emissions. Values not in parentheses are tolls set for off-peak hour periods, while values in parentheses show tolls for peak hour periods.

3. Result Data Table

Table S4 provides summary results for all strategies. It should be noted that the total exposure results from the baseline run here are smaller than the ones that were obtained in Bin Thaneya et al.[2] This is attributed to the difference in assumptions in the trip demand data used. The trip demand data used in Bin Thaneya et al.[2] is limited in that it did not provide trip data disaggregated by TOD period. Due to the lack of data, it was assumed that the total amount of trips that were provided were taking place hourly, which is equivalent to the number of trips that were taking place during peak traffic periods in this network on average. The network used was also smaller than the one obtained from CMAP. Thus, the large number of trips assuming to be taking place on an hourly basis led to the large magnitude of exposure that was quantified.

Population-weighted concentrations in **Table S3** are calculated using **(12)**.

$$\text{Pop Wtd Conc.}_{\text{m}} = \frac{\sum_{m \in M} C_m Pop_m}{\sum_{m \in M} Pop_m} \quad \mathbf{(12)}$$

where:

C_m: $PM_{2.5}$ concentration in exposure zone m

Pop_m: population numbers in exposure zone m

Table S4 TA exposure and travel time results of the baseline run and exposure mitigation strategies. Values in parentheses show % differences relative to the baseline (UET) run. Exposure results are shown for the entire exposure domain.

Description	Population Annual Intake [kg$PM_{2.5}$/y]	Population Weighted Concentration [μg/m^3]	Exposure Damages [$B/y]	Total Travel Time [10^9hours/y]	Total Travel Costs [$B/y]	Total Costs [$B/y]
**User-Equilibrium for Time (UET)**	38.5	0.0925	3.66 - 8.26	2.99	53.3	57.0 - 61.6
**Pareto (UET 90 : SOI 10)**	37.7 (-2.08%)	0.0906 (-2.05%)	3.59 - 8.10 (-1.97% - -1.91%)	3.08 (+3.01%)	54.9 (+3.01%)	58.5 - 63.0 (+2.34% - +2.69%)
**Pareto (UET 75 : SOI 25)**	36.9 (-4.16%)	0.0887 (-4.11%)	3.51 - 7.92 (-4.20% - -4.10%)	3.24 (+8.36%)	57.8 (+8.36%)	61.3 - 65.7 (+6.76% - +7.64%)

**Pareto (UET 50 : SOI 50)**	36.1 (-6.23%)	0.0867 (-6.27%)	3.44 - 7.75 (-6.29% - -6.01%)	3.55 (+18.7%)	63.3 (+18.7%)	66.7 - 71.1 (+15.4% - +17.2%)
**Pareto (UET 25 : SOI 75)**	35.7 (-7.27%)	0.0857 (-7.35%)	3.40 - 7.67 (-7.28% - -7.10%)	3.97 (+32.8%)	70.8 (+32.8%)	74.2 - 78.5 (+27.5% - +30.3%)
**Pareto (UET 10 : SOI 90)**	35.4 (-8.05%)	0.0851 (-8.00%)	3.37 - 7.59 (-8.27% - -7.92%)	4.47 (+49.5%)	79.7 (+49.5%)	83.1 - 87.3 (+41.8% - +45.8%)
**System Optimal for Intake (SOI)**	35.3 (-8.31%)	0.0848 (-8.32%)	3.36 - 7.58 (-8.23% - -8.20%)	4.96 (+65.9%)	88.2 (+65.9%)	91.6 - 95.8 (+55.6% - +60.7%)
**2030 Fleet (Base Grid)**	31.9 (-17.1%)	0.0766 (-17.2%)	3.09 - 6.97 (-15.7% - -15.6%)	2.99 (0.00%)	2.99 (0.00%)	56.4 - 60.3 (-2.17% - -1.07%)
**2030 Fleet (Future Grid)**	29.9 (-22.3%)	0.0719 (-22.3%)	2.88 - 6.49 (-21.4% - -21.3%)	2.99 (0.00%)	2.99 (0.00%)	56.2 - 59.8 (-2.96% - -1.44%)
**2040 Fleet (Base Grid)**	40.7 (+5.71%)	0.0978 (+5.72%)	4.24 - 9.56 (+15.7% - 15.8%)	2.99 (0.00%)	2.99 (0.00%)	57.5 - 62.9 (+0.945% - +2.06%)
**2040 Fleet (Future Grid)**	26.1 (-32.2%)	0.0629 (-32.0%)	2.59 - 5.83 (-29.4% - -29.2%)	2.99 (0.00%)	2.99 (0.00%)	55.9 - 59.1 (-4.03% - -1.95%)
**2050 Fleet (Base Grid)**	43.0 (+11.7%)	0.103 (+11.4%)	4.54 - 10.2 (+23.5% - +24.0%)	2.99 (0.00%)	2.99 (0.00%)	57.8 - 63.5 (+1.47% - +3.11%)
**2050 Fleet (Future Grid)**	23.0 (-40.3%)	0.0554 (-40.1%)	2.27 - 5.10 (-38.3% - -38.0%)	2.99 (0.00%)	2.99 (0.00%)	55.6 - 58.4 (-5.23% - -2.51%)
**Particle Filtration (LE 90th Perc.)**	37.2 (-3.38%)	0.0895 (-3.24%)	3.54 - 7.98 (-3.39% - -3.28%)	2.99 (0.00%)	2.99 (0.00%)	56.8 - 61.3 (-0.519% - -0.281%)
**Particle Filtration (HE 90th Perc.)**	36.3 (-5.71%)	0.0873 (-5.62%)	3.46 - 7.79 (-5.69% - -5.46%)	2.99 (0.00%)	2.99 (0.00%)	56.8 - 61.1 (-0.828% - -0.421%)
**Particle Filtration (LE 75th Perc.)**	34.1 (-11.4%)	0.0819 (-11.5%)	3.25 - 7.33 (-11.3% - -11.2%)	2.99 (0.00%)	2.99 (0.00%)	56.6 - 60.6 (-1.57% - -0.789%)
**Particle Filtration (HE 75th Perc.)**	30.9 (-19.7%)	0.0743 (-19.7%)	2.96 - 6.67 (-19.2% - -19.1%)	2.99 (0.00%)	2.99 (0.00%)	56.3 - 60.0 (-2.65% - -1.30%)
**Particle Filtration (LE 50th Perc.)**	29.6 (-23.1%)	0.0711 (-23.1%)	2.84 - 6.40 (-22.5% - -22.4%)	2.99 (0.00%)	2.99 (0.00%)	56.1 - 59.7 (-3.08% - -1.51%)
**Particle Filtration (HE 50th Perc.)**	23.2 (-39.7%)	0.0558 (-39.7%)	2.26 - 5.09 (-38.4% - -38.3%)	2.99 (0.00%)	2.99 (0.00%)	55.6 - 58.4 (-5.21% - -2.53%)
**Increase Public Transport Trips (+5%)**	38.1 (-1.04%)	0.0917 (-0.865%)	3.63 - 8.18 (-0.969% - -0.820%)	2.96 (-1.00%)	52.78 (-1.00%)	56.9 - 61.5 (-0.195% - -0.123%)
**Increase Public Transport Trips (+10%)**	37.8 (-1.82%)	0.0909 (-1.73%)	3.60 - 8.12 (-1.69% - -1.64%)	2.94 (-1.67%)	52.4 (-1.67%)	56.9 - 61.4 (-0.292% - -0.175%)

**Increase Public Transport Trips (+20%)**	37.2 (-3.38%)	0.0895 (-3.24%)	3.54 - 7.98 (-3.39% - -3.28%)	2.90 (-3.01%)	51.7 (-3.01%)	56.8 - 61.3 (-0.520% - -0.281%)
**Increase Public Transport Trips (+40%)**	36.1 (-6.23%)	0.0867 (-6.27%)	3.44 - 7.74 (-6.30% - -6.01%)	2.81 (-6.02%)	50.1 (-6.02%)	56.7 - 61.0 (-0.909% - -0.456%)
**Rerouting Trucks**	29.0 (-24.7%)	0.0695 (-24.9%)	2.76 - 6.22 (-24.7% - -24.6%)	2.53 (-15.4%)	45.1 (-15.4%)	56.1 - 59.5 (-3.38% - -1.65%)

4. References

(1) Chicago Metropolitan Agency for Planning. *On To 2050 Travel Demand Model Documentation*; Chicago Metropolitan Agency for Planning, 2018. https://www.cmap.illinois.gov/documents/10180/911391/FINAL+Travel+Demand+Model+Documentation+Appendix.pdf/f3b1322c-2e60-2513-720f-38ee68b799d1.

(2) Bin Thaneya, A.; Apte, J. S.; Horvath, A. A Human Exposure-Based Traffic Assignment Model for Minimizing Fine Particulate Matter (PM$\less$sub$\greater$2.5$\less$/Sub$\greater$) Intake from on-Road Vehicle Emissions. *Environ. Res. Lett.* **2022**, *17* (7), 074034. https://doi.org/10.1088/1748-9326/ac78f6.

(3) Webster, F. V.; Cobbe, B. M. *Traffic Signals*; H.M. Stationery Office, 1966.

(4) United States Department of Transportation. Revised Departmental Guidance on Valuation of Travel Time in Economic Analysis. **2016**. https://www.transportation.gov/office-policy/transportation-policy/revised-departmental-guidance-valuation-travel-time-economic.

(5) Tal, G.; Raghavan, S. S.; Karanam, V. C.; Favetti, M. P.; Sutton, K. M.; Lee, J. H.; Nitta, C.; Chakraborty, D.; Nicholas, M.; Turrentine, T. Advanced Plug-in Electric Vehicle Travel and Charging Behavior Final Report. *California Air Resources Board Contract* **2020**, 12–319.

(6) Chester, M.; Horvath, A. Environmental Assessment of Passenger Transportation Should Include Infrastructure and Supply Chains. *Environ. Res. Lett.* **2009**, *4* (2), 024008. https://doi.org/10.1088/1748-9326/4/2/024008.

(7) Tessum, C. W.; Apte, J. S.; Goodkind, A. L.; Muller, N. Z.; Mullins, K. A.; Paolella, D. A.; Polasky, S.; Springer, N. P.; Thakrar, S. K.; Marshall, J. D.; Hill, J. D. Inequity in Consumption of Goods and Services Adds to Racial–Ethnic Disparities in Air Pollution Exposure. *Proceedings of the National Academy of Sciences* **2019**, *116* (13), 6001–6006. https://doi.org/10.1073/pnas.1818859116.

(8) Apte, J. S.; Marshall, J. D.; Cohen, A. J.; Brauer, M. Addressing Global Mortality from Ambient PM2.5. *Environ. Sci. Technol.* **2015**, *49* (13), 8057–8066. https://doi.org/10.1021/acs.est.5b01236.

(9) U.S. Environmental Protection Agency. Mortality Risk Valuation. **2022**. https://www.epa.gov/environmental-economics/mortality-risk-valuation.

(10) Nasari, M. M.; Szyszkowicz, M.; Chen, H.; Crouse, D.; Turner, M. C.; Jerrett, M.; Pope, C. A.; Hubbell, B.; Fann, N.; Cohen, A.; Gapstur, S. M.; Diver, W. R.; Stieb, D.; Forouzanfar, M. H.; Kim, S.-Y.; Olives, C.; Krewski, D.; Burnett, R. T. A Class of Non-Linear Exposure-Response Models Suitable for Health Impact Assessment Applicable to Large Cohort Studies of Ambient Air Pollution. *Air Qual Atmos Health* **2016**, *9* (8), 961–972. https://doi.org/10.1007/s11869-016-0398-z.

(11) Burnett, R.; Chen, H.; Szyszkowicz, M.; Fann, N.; Hubbell, B.; Pope, C. A.; Apte, J. S.; Brauer, M.; Cohen, A.; Weichenthal, S.; Coggins, J.; Di, Q.; Brunekreef, B.; Frostad, J.; Lim, S. S.; Kan, H.; Walker, K. D.; Thurston, G. D.; Hayes, R. B.; Lim, C. C.; Turner, M. C.; Jerrett, M.; Krewski, D.; Gapstur, S. M.; Diver, W. R.; Ostro, B.; Goldberg, D.; Crouse, D. L.; Martin, R. V.; Peters, P.; Pinault, L.; Tjepkema, M.; van Donkelaar, A.; Villeneuve, P. J.; Miller, A. B.; Yin, P.; Zhou, M.; Wang, L.; Janssen, N. A. H.; Marra, M.; Atkinson, R. W.; Tsang, H.; Quoc Thach, T.; Cannon, J. B.; Allen, R. T.; Hart, J. E.; Laden, F.; Cesaroni, G.; Forastiere, F.; Weinmayr, G.; Jaensch, A.; Nagel, G.; Concin, H.; Spadaro, J.

V. Global Estimates of Mortality Associated with Long-Term Exposure to Outdoor Fine Particulate Matter. *Proc Natl Acad Sci U S A* **2018**, *115* (38), 9592–9597. https://doi.org/10.1073/pnas.1803222115.
(12) Krewski, D.; Jerrett, M.; Burnett, R. T.; Ma, R.; Hughes, E.; Shi, Y.; Turner, M. C.; Pope, C. A.; Thurston, G.; Calle, E. E.; Thun, M. J.; Beckerman, B.; DeLuca, P.; Finkelstein, N.; Ito, K.; Moore, D. K.; Newbold, K. B.; Ramsay, T.; Ross, Z.; Shin, H.; Tempalski, B. Extended Follow-up and Spatial Analysis of the American Cancer Society Study Linking Particulate Air Pollution and Mortality. *Res Rep Health Eff Inst* **2009**, No. 140, 5–114; discussion 115-136.
(13) Lepeule, J.; Laden, F.; Dockery, D.; Schwartz, J. Chronic Exposure to Fine Particles and Mortality: An Extended Follow-up of the Harvard Six Cities Study from 1974 to 2009. *Environmental Health Perspectives* **2012**, *120* (7), 965–970. https://doi.org/10.1289/ehp.1104660.
(14) Air Resources Board. Mobile Source Emission Inventory - EMFAC2021 Database. **2021**. https://arb.ca.gov/emfac/.
(15) Bin Thaneya, A.; Horvath, A. Exploring Regional Fine Particulate Matter (PM2.5) Exposure Reduction Pathways Using an Optimal Power Flow Model: The Case of the Illinois Power Grid. *Environ. Sci. Technol.* **2023**. https://doi.org/10.1021/acs.est.2c08698.
(16) Maestas, M. M.; Brook, R. D.; Ziemba, R. A.; Li, F.; Crane, R. C.; Klaver, Z. M.; Bard, R. L.; Spino, C. A.; Adar, S. D.; Morishita, M. Reduction of Personal PM2.5 Exposure via Indoor Air Filtration Systems in Detroit: An Intervention Study. *J Expo Sci Environ Epidemiol* **2019**, *29* (4), 484–490. https://doi.org/10.1038/s41370-018-0085-2.
(17) United States Census Bureau. American Community Survey 5-Year Estimates (Washington, DC: US Department of Commerce). **2019**. https://doi.org/www.census.gov/programssurveys/acs/.
(18) Holmes. *Holmes HAPF300AHD Aer1 True HEPA Allergen Remover Air Purifier Filter*; 2023. https://www.holmesproducts.com/filters/air-purifier-filters/holmes-hapf300ahd-aer1-true-hepa-allergen-remover-air-purifier-filter-2-pack/SAP_2097796.html (accessed 2023-10-12).
(19) Holmes. *Holmes HEPA Type Filter HAPF30D-U2, Filter A*; 2023. https://camelcamelcamel.com/product/B000065DKD (accessed 2023-10-12).
(20) Holmes. *Holmes Aer1 Tower HEPA Air Purifier with Air Ionizer and Visipure Filter Viewing Window, Small Room Air Cleaner & Allergen Remover - Black (HAP9425B-TU)*; 2023. https://www.holmesproducts.com/air-purifiers/tower/holmes-aer1-tower-hepa-air-purifier-with-air-ionizer-and-visipure-filter-viewing-window-small-room-air-cleaner-allergen-remover---black--hap9425b-tu/SAP_2098632.html (accessed 2023-10-12).
(21) Metra. Annual and Monthly Ridership | Metra. **2022**. https://metra.com/annual-and-monthly-ridership.
(22) Pace. Pace Annual Reports | Pace Suburban Bus. **2022**. https://www.pacebus.com/pace-annual-reports.
(23) Chicago Transit Authority. Ridership Reports - Performance. *CTA* **2022**. https://www.transitchicago.com/ridership/.
(24) Kavvada, I.; Moura, S.; Horvath, A. Aligning Sustainability and Regional Earthquake Hazard Mitigation Planning: Integrating Greenhouse Gas Emissions and Vertical Equity. *Environ. Res.: Infrastruct. Sustain.* **2022**, *2* (4), 045013. https://doi.org/10.1088/2634-4505/aca9f3.

(25) Arora, J. S. 17 - Multiobjective Optimum Design Concepts and Methods. In *Introduction to Optimum Design (Second Edition)*; Arora, J. S., Ed.; Academic Press: San Diego, 2004; pp 543–563. https://doi.org/10.1016/B978-012064155-0/50017-3.
(26) Kim, I. Y.; de Weck, O. L. Adaptive Weighted-Sum Method for Bi-Objective Optimization: Pareto Front Generation. *Struct Multidisc Optim* **2005**, *29* (2), 149–158. https://doi.org/10.1007/s00158-004-0465-1.
(27) Grodzevich, O.; Romanko, O. *Normalization and Other Topics in Multi-Objective Optimization*; 2006.
(28) Yin, Y.; Lawphongpanich, S. Internalizing Emission Externality on Road Networks. *Transportation Research Part D: Transport and Environment* **2006**, *11* (4), 292–301. https://doi.org/10.1016/j.trd.2006.05.003.
